# A photoperiodic time measurement served by the biphasic expression of *Cryptochrome1ab* in the zebrafish eye

**DOI:** 10.1038/s41598-020-61877-4

**Published:** 2020-03-19

**Authors:** Keiko Okano, Yuya Saratani, Ayumi Tamasawa, Yosuke Shoji, Riko Toda, Toshiyuki Okano

**Affiliations:** Department of Electrical Engineering and Bioscience, Graduate School of Sciences and Engineering, Waseda University, TWIns, Wakamatsucho 2-2, Shinjuku-Ku, Tokyo 162-8480 Japan

**Keywords:** Circadian rhythms, Circadian mechanisms

## Abstract

The zebrafish (*Danio rerio*) is a model species that is used to study the circadian clock. It possesses light-entrainable circadian clocks in both central and peripheral tissues, and its core circadian factor cryptochromes (CRYs) have diverged significantly during evolution. In order to elucidate the functional diversity and involvement of CRYs in photoperiodic mechanisms, we investigated the daily expression profiles of six *Cry* transcripts in central (brain and eye) and peripheral (fin, skin and muscle) tissues. The *zCry* genes exhibited gene-specific diurnal conserved variations, and were divided into morning and evening groups. Notably, *zCry1ab* exhibited biphasic expression profiles in the eye, with peaks in the morning and evening. Comparing ocular *zCry1ab* expression in different photoperiods (18L:6D, 14L:10D, 10L:14D and 6L:18D) revealed that *zCry1ab* expression duration changed depending on the photoperiod: it increased at midnight and peaked before lights off. *zCry1ab* expression in constant light or dark after entrainment under long- or short-day conditions suggested that the evening clock and photic input pathway are involved in photoperiod-dependent *zCry1ab* expression. Laser microdissection followed by qRT-PCR analysis showed that the evening peak of *zCry1ab* was likely ascribed to visual photoreceptors. These results suggest the presence of an eye-specific photoperiodic time measurement served by *zCry1ab*.

## Introduction

The circadian clock consists of three parts: the input pathway(s), the oscillation system and the output pathway(s). Light is the most important signal that activates the input pathway to synchronize the oscillator with daily external cycles. The oscillator generates time signals to control physiological circadian functions through the output pathway. Internal time signals are integrated with the environmental light signal to measure day length, which triggers seasonal photoperiodic responses in many organisms living in temperate zones^1^.

The zebrafish (*Danio rerio*) circadian clock is one of the most studied among fish species, and is reported to exist in not only the central tissues but also the embryo, larva, peripheral tissues and cultured cells (refs. ^2–11^ in Table 1, refs. ^12–15^). These zebrafish circadian clocks are directly light-entrainable and this feature is also seen in the other fish cells^16^ in contrast to the mammalian circadian clocks that are not light-entrainable except for those in the eye.

**Table 1:** Summary of cryptochrome expressions in zebrafish tissues and cell lines.

Gene	Tissue/cell	Max^#^	Acrophase	Light cycle	Method^$,&^	Ref.
*zCry1aa*	Eye	ZT1	—	14L10D	RPA	^2^
	Eye	ZT23-ZT11*	—	14L10D	qRT-PCR	^3^
	Eye	ZT1*	ZT4.8	14L10D	qRT-PCR	this work
	Brain	ZT9	—	12L12D	qRT-PCR	^4^
	Brain	ZT7*	ZT5.9	14L10D	qRT-PCR	this work
	Skin	ZT3	ZT5.04	14L10D	qRT-PCR	^5^
	Skin	CT3	CT4.11	DD	qRT-PCR	^5^
	Skin	ZT7*	ZT5.5	14L10D	qRT-PCR	this work
	Fin	ZT7*	ZT5.1	14L10D	qRT-PCR	this work
	Muscle	ZT7*	ZT6.9	14L10D	qRT-PCR	this work
	Liver	ZT3	—	12L12D	qRT-PCR	^4^
	Larva	ZT4	—	12L12D	qRT-PCR	^6^
	PAC-2	—	—	12L12D	BLA	^7^
*zCry1ab*	Eye	ZT4	—	L12D12	RPA	^2^
	Eye	ZT23-ZT15*	—	14L10D	qRT-PCR	^3^
	Eye	ZT1*	—	14L10D	qRT-PCR	this work
	Brain	ZT1*	ZT3.3	14L10D	qRT-PCR	this work
	Skin	ZT1*	ZT4.1	14L10D	qRT-PCR	this work
	Fin	ZT1*	ZT3.4	14L10D	qRT-PCR	this work
	Muscle	ZT1*	ZT5.3	14L10D	qRT-PCR	this work
	Larva	—	CT6.46	14L10D/DD	Microarray	^8^
	Larva	ZT4	—	14L10D	qRT-PCR	^6^
	ZEM-2S	ZT21-ZT12*	—	12L12D	qRT-PCR	^9^
	ZEM-2S	—	—	DD	qRT-PCR	^9^
	ZEM-2S	ZT0*, ZT12*	—	12L12D	qRT-PCR	^10^
	ZEM-2S	—	—	DD	qRT-PCR	^10^
*zCry1ba*	Eye	ZT13-ZT15	—	14L10D	RPA	^2^
	Eye	CT13	—	DD	RPA	^2^
	Eye	CT13	—	LL	RPA	^2^
	Eye	ZT7-ZT19*	—	14L10D	qRT-PCR	^3^
	Eye	ZT15*	ZT13.1	14L10D	qRT-PCR	this work
	Brain	ZT15*	ZT11.4	14L10D	qRT-PCR	this work
	Skin	ZT15*	ZT13.0	14L10D	qRT-PCR	this work
	Fin	ZT15*	ZT11.3	14L10D	qRT-PCR	this work
	Muscle	ZT15*	ZT12.0	14L10D	qRT-PCR	this work
	Larva	ZT12-ZT16	CT13.33	14L10D/DD	Microarray	^8^
	Larva	ZT9-ZT15*	—	12L12D	qRT-PCR	^11^
	Larva	ZT12	—	12L12D	qRT-PCR	^6^
*zCry1bb*	Eye	ZT13-ZT15	—	14L10D	RPA	^2^
	Eye	CT9-CT13	—	LL	RPA	^2^
	Eye	CT13	—	DD	RPA	^2^
	Eye	ZT7-ZT15*	—	14L10D	qRT-PCR	^3^
	Eye	ZT15*	ZT11.9	14L10D	qRT-PCR	this work
	Brain	ZT15*	ZT11.9	14L10D	qRT-PCR	this work
	Skin	ZT15*	ZT13.4	14L10D	qRT-PCR	this work
	Fin	ZT15*	ZT12.0	14L10D	qRT-PCR	this work
	Muscle	ZT15*	ZT11.9	14L10D	qRT-PCR	this work
	Larva	—	CT14.08	14L10D/DD	Microarray	^8^
	Larva	ZT12	—	12L12D	qRT-PCR	^6^
*zCry2*	Eye	ZT23-ZT1	—	14L10D	RPA	^2^
	Eye	CT23	—	DD	RPA	^2^
	Eye	—	—	LL	RPA	^2^
	Eye	ZT19-ZT7*	—	14L10D	qRT-PCR	^3^
	Eye	ZT1*	ZT0.5	14L10D	qRT-PCR	this work
	Brain	ZT1*	ZT1.1	14L10D	qRT-PCR	this work
	Skin	ZT1*	ZT0.9	14L10D	qRT-PCR	this work
	Fin	ZT1*	ZT1.3	14L10D	qRT-PCR	this work
	Muscle	ZT1*	ZT1.4	14L10D	qRT-PCR	this work
	Larva	—	CT2.17	14L10D/DD	Microarray	^8^
	Larva	ZT0-ZT4	—	12L12D	qRT-PCR	^6^
*zCry4*	Eye	ZT9	—	14L10D	RPA	^2^
	Eye	ZT3-ZT15*	—	14L10D	qRT-PCR	^3^
	Eye	ZT15*	ZT11.7	14L10D	qRT-PCR	this work
	Brain	ZT15*	ZT12.1	14L10D	qRT-PCR	this work
	Skin	ZT15*	ZT12.5	14L10D	qRT-PCR	this work
	Fin	ZT15*	ZT12.5	14L10D	qRT-PCR	this work
	Muscle	ZT7*	ZT8.2	14L10D	qRT-PCR	this work
	Larva	—	CT14.29	14L10D/DD	Microarray	^8^
	Larva	ZT12	—	12L12D	qRT-PCR	^6^

^#^Highest value among the data; *Statistically significant; —, Not determined;

^$^RPA, RNase protection assay; ^&^BLA, Bioluminescence assay.

Among vertebrate core clock components such as period (PER), CLOCK, BMAL and cryptochrome (CRY) proteins, CRYs have both a wide evolutionary background and divergent molecular functions^6^. CRYs are structurally classified into several groups, and together form a large protein family with the photo repair enzymes, photolyases (PHRs). Among CRY/PHR family proteins, animal-type CRYs (CRY1 and CRY2) play a central role in the circadian clock oscillator. Animal-type CRY1 and CRY2 function as transcriptional repressors in the core loop of the circadian clock oscillation system^17,18^, while fruit fly CRY (dCRY) and other non-mammalian CRYs serve as blue light photoreceptor molecules using flavin adenine dinucleotide (FAD) as a chromophore^19^. In tropical fish, animal-type CRY2 (termed CRY3 in refs. ^20–22^) may function for the lunar timer^20–22^. In addition, CRY4 was found in the chicken pineal gland^23^ and retina^24^ and shown to absorb light to change its structure^25^. Biophysical analyses using recombinant CRY4 proteins indicated their chromophore FAD binding and photoreceptive functions^25,26^, and hence CRY4 is strongly suggested to be a photoreceptor and/or a light-driven magnetoreceptor^27^.

Six *Cry* gene paralogues have been reported in zebrafish: four animal-type *Cry1*s (*zCry1aa*, *zCry1ab*, *zCry1ba* and *zCry1bb*), one animal-type *Cry2* (*zCry2*; termed *zCry3* in ref. ^2^) and one *Cry4* (*zCry4*; termed *zCry3* in ref. ^6^)^2,6^. These genes have evolved through multiple gene duplication events during teleost evolution^6^, but their functional differences and redundancies are not fully understood. While the animal-type CRY2 (zCRY2) and zCRY4 have only weak or no transcriptional repression ability for the CLOCK:BMAL complex, the four animal-type CRY1s have strong transcriptional repression abilities^14^, so may not only be involved in the circadian clock but also in clock-associated functions, such as the photoperiodic response. Although daily *zCry* expression patterns have been investigated both *in vivo*^2–6,8,11^ and *in vitro*^7,9,10^, we comparatively reinvestigated the spatiotemporal expression profiles and light-responsiveness of *zCry*s to test the above hypothesis. We found that *zCry1ab* expression in the eye changes depending on the day length. Based on the *zCry1ab* expression profiles, we suggest a model of *zCry1ab* expression regulation in the retinal photoreceptors, in which the light and time information may encode the environmental photoperiod into transcriptional regulatory signals.

## Results

### Daily variations in *zCry* mRNA expression levels

**Figure 1:**
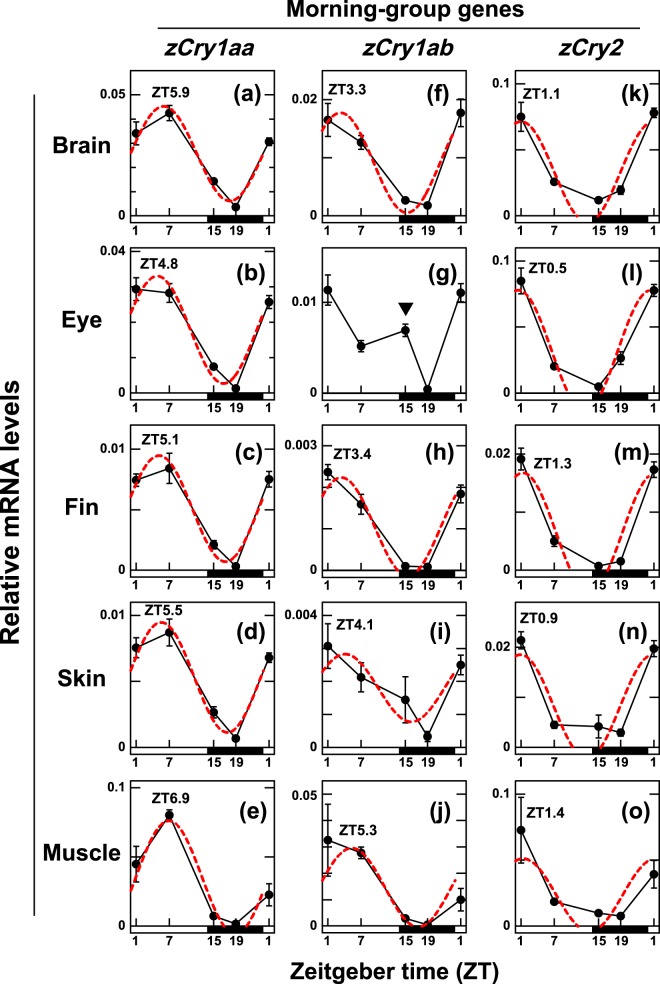
Daily profiles of *zCry1aa, zCry1ab* and *zCry2* mRNA expression levels in central and peripheral tissues under white light/dark cycles. Each tissue was collected at ZT1, ZT7, ZT15 or ZT19 from zebrafish (n = 4) entrained to white light/dark cycles (14L:10D). Each mRNA level was estimated using *zβ-actin* as a reference gene because it was the most stably expressed in the examined tissues among the control genes. Data were analyzed by one-way ANOVA (Supplementary Table S1) and Tukey-Kramer post-hoc tests (Supplementary Tables S2–S31). Error bars represent ± SE. Peak times of cosine-fitting to the data as calculated by CircWave1.4 (red dashed curves; Supplementary Table S32) are shown. A down-pointing triangle in panel g denotes the sustained expression of *zCry1ab* transcripts at ZT15 in the eye.

**Figure 2:**
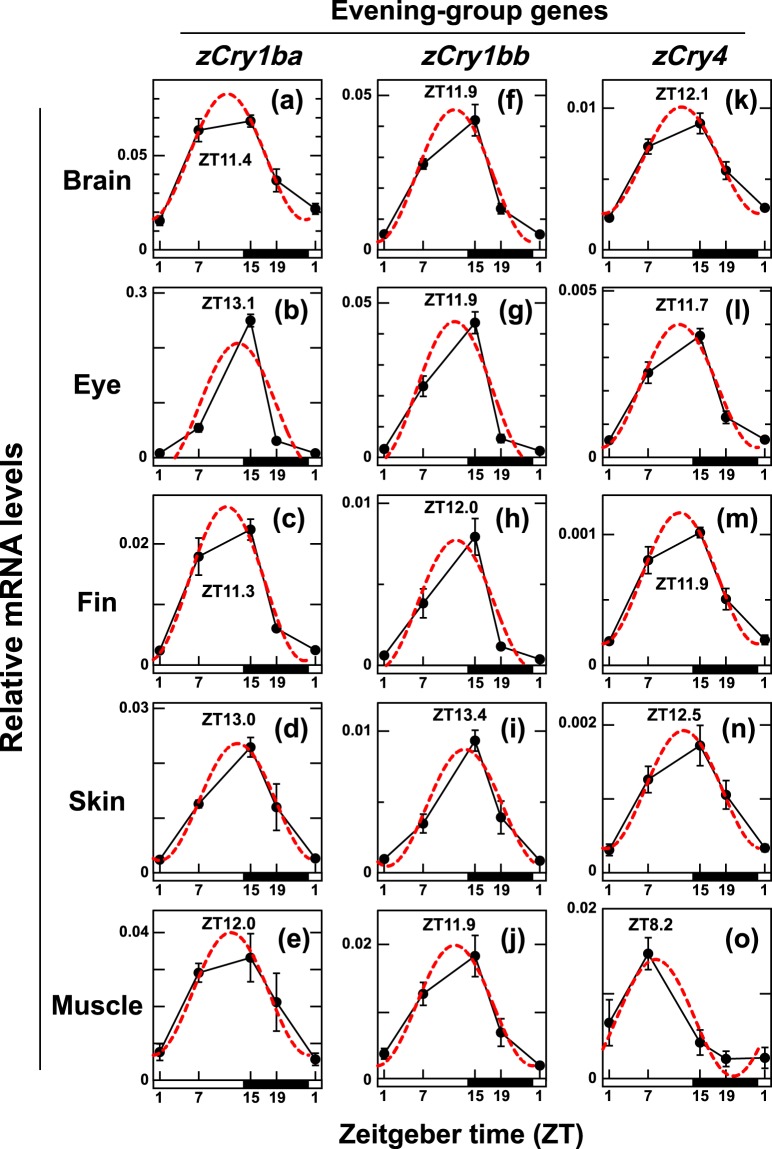
Daily profiles of *zCry1ba, zCry1bb* and *zCry4* mRNA expression levels in central and peripheral tissues under white light/dark cycles. The experimental conditions and data analyses were the same as those described in Fig. 1.

**Figure 3:**
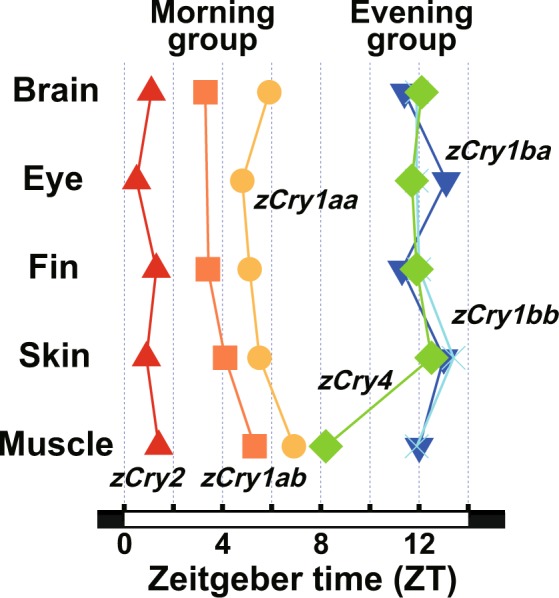
Maximum *Cry* expression in zebrafish tissues. Acrophases of *Cry* expression as estimated by cosinor analysis (Supplementary Table S32) are shown. Red up-pointing triangles, orange squares, yellow circles, green diamonds, dark-blue down-pointing triangles and light-blue crosses show estimated acrophases for *zCry2*, *zCry1ab*, *zCry1aa*, *zCry4*, *zCry1ba* and *zCry1bb*, respectively.

We examined the transcript levels of six *Cry* genes in the brain, eye, pectoral fins, skin and muscle at five time points (ZT1, ZT7, ZT15, ZT19 and ZT1) in a white light, long-day (LD; 14L:10D) cycle by qRT-PCR (Figs. 1 and 2, Supplementary Tables S1–S31), and their acrophases were estimated by cosinor-fitting analyses (Fig. 3, Supplementary Table S32).

In all tissues examined, *zCry* mRNA expression levels exhibited daily changes (*p* < 0.01, Supplementary Table S1), indicating that their expression was under the control of the circadian clock and/or environmental light. The expression patterns of every gene investigated were similar across the tissues (Figs. 1 and 2), except for a few cases (see below), and the z*Cry* genes were classified into two groups based on their daily expression patterns (Fig. 3, Supplementary Table S32): genes in the ‘morning group’ were *zCry1aa*, *zCry1ab* and *zCry2* (Fig. 1), the expression patterns of which were well-fitted to curves with peaks in the first half of the light period (ZT0.5-ZT6.9), while genes in the ‘evening group’ were *zCry1ba*, *zCry1bb* and *zCry4* (Fig. 2), which were well-fitted to curves with peaks at the start of the dark period (ZT11.3-ZT13.4), except for *zCry4* in the muscle (ZT8.2) (Figs. 2 and 3).

Interestingly, *zCry1ab* exhibited sustained expression at ZT15 in the eye (the down-pointing triangle in Fig. 1g; *p* < 0.01 vs. ZT19, Supplementary Table S8), suggesting possible biphasic expression, which was not observed in any other tissue examined (Fig. 1f,h–j).

### Circadian and photic regulation of *zCry* expression

**Figure 4:**
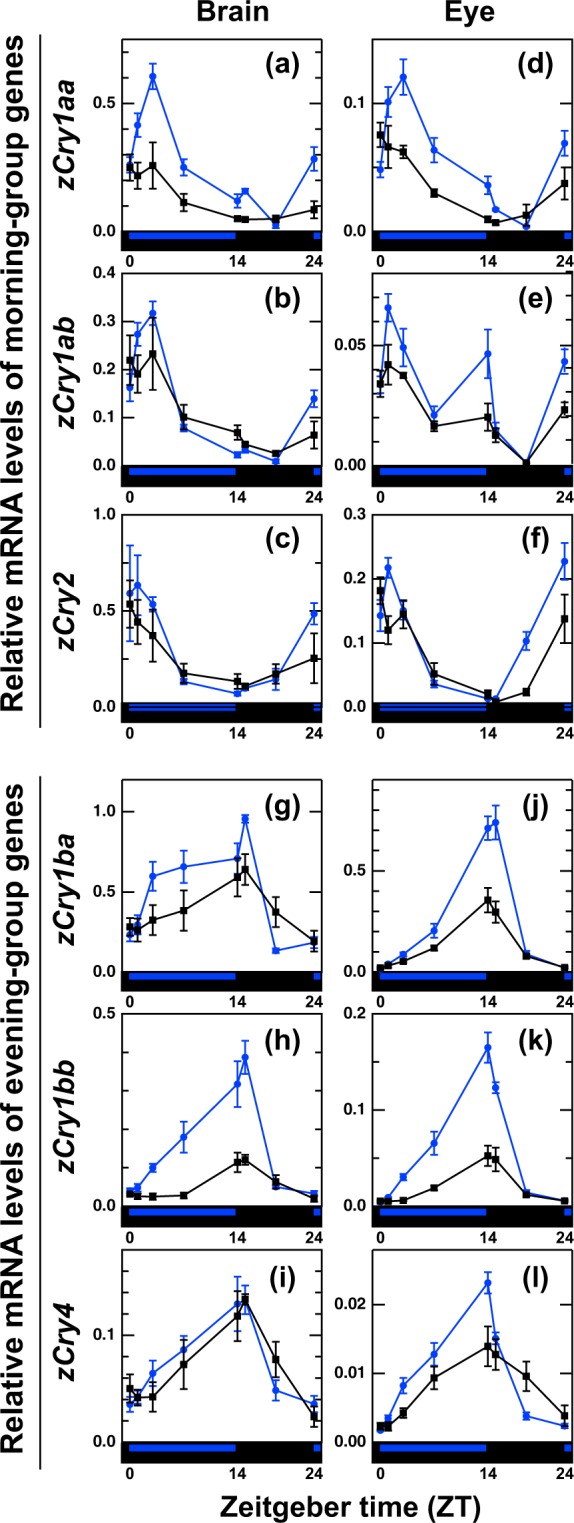
*zCry* mRNA levels under blue light/dark and dark conditions. Zebrafish were entrained to white light/dark cycles (14L:10D) and irradiated with blue (λ_max_ = 462 nm; λ_1/2_ = 453 nm and 573 nm; blue circles) light or kept in darkness (black squares) at 28 °C for 14 h from ZT0 (Supplementary Fig. S1). Light intensities were set at 5.586 × 10^14^ photons s^−1^ m^−2^ that corresponds to 240 μW cm^−2^. Tissues were collected from three (brain samples at ZT0 and ZT19, and eye samples at ZT7 and ZT19) or four (other samples) zebrafish at ZT0, ZT1, ZT3, ZT7, ZT14, ZT15, ZT19 or ZT0 (ZT24) (see Methods for details). Each mRNA level was estimated as a relative value to the geometric mean of mRNA levels of *zβ-actin*, *zGapdh* and *zEf1α* (arithmetic means of their CT values were used in the 2^−ΔΔCT^ method because they were stably expressed in the brain and eye). Data were analyzed by two-way ANOVA (Supplementary Table S33) and Tukey-Kramer post-hoc tests (Supplementary Tables S34–S57). Error bars represent ± SE.

We next investigated the expression patterns of *zCry*s under blue light or dark to further characterize their circadian and photic regulations (Fig. 4). Here we focused on the brain and eye, because (i) expression patterns were not substantially different across the tissues (Figs. 1 and 2) and (ii) the expression levels of every *Cry* were relatively high in these tissues.

Zebrafish were entrained to the 14L:10D cycle and then exposed to blue light (Supplementary Fig. S1) for 14 h from ZT0 or kept in the dark. To evaluate the temporal effects of changing the light conditions, we changed sampling points to include ‘lights on’ and the mid-points of the light and dark periods (ZT0, ZT1, ZT3, ZT7, ZT14, ZT15, ZT19 and ZT0).

In the eye, light effects were observed in all the examined *zCry* genes (Fig. 4d–f,j–l; interaction *p* < 0.05, Supplementary Table S33). The *zCry1aa* expression level under the blue light condition was higher at ZT3 than that under the dark condition (*p* < 0.01, Supplementary Table S35). Notably, the eye-specific biphasic expression of *zCry1ab* (Fig. 1g) was reproduced with peaks in both the morning and evening under the blue light condition (Fig. 4e). The *zCry1ab* expression level at the evening peak was significantly higher than that in the dark (blue light vs dark at ZT14; *p* < 0.05, Supplementary Table S37). Similarly, all the evening-group genes showed the light-dependent upregulation in the evening (ZT14, Fig. 4j–l; *p* < 0.01, Supplementary Tables S39, S41, and S45). Under the dark condition, all the morning- and evening-group *zCry* genes exhibited significant temporal variations with peaks in the morning and evening, respectively (Supplementary Tables S33, S35, S37, S39, S41, S43, and S45). These results suggested that both the morning and evening *zCry* gene expressions are controlled by the circadian clock and additionally regulated by the light signal in the eye.

In the brain, expressions of the four *zCry1* genes were affected by the light (Fig. 4a,b,g,h; interaction *p* < 0.05, Supplementary Tables S33, S34, S36, S38, and S40), but those of *zCry2* and *zCry4* were not (Fig. 4c,i; Supplementary Tables S33, S42, and S44). Light-dependent upregulations were observed in *zCry1aa* (Fig. 4a; *p* < 0.01, blue light vs dark at ZT3, Supplementary Table S34) and *zCry1bb* (Fig. 4h; *p* < 0.01 in the blue light vs dark at ZT7, ZT14, and ZT15, Supplementary Table S40), but not in the other *zCry* genes. Under the dark condition, no significant temporal variation was observed in *zCry1aa*, *zCry1ba*, and *zCry1bb*, although they showed weak variations (Fig. 4a,g,h; Supplementary Tables S34, S38, and S40).

### Photoperiodic control of the *zCry1ab* mRNA level

**Figure 5:**
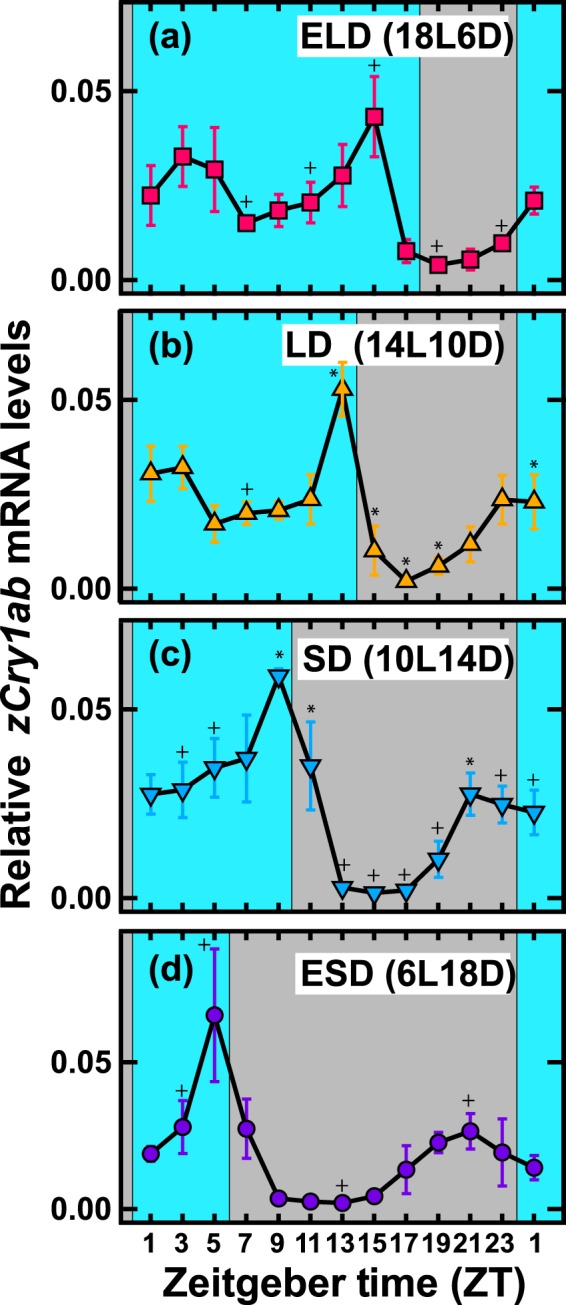
Daily profiles of *zCry1ab* mRNA expression levels in the eye under different photoperiods. Zebrafish were entrained to 18L:6D extra-long-day (ELD; panel a), 14L:10D long-day (LD; panel b), 10L:14D short-day (SD; panel c) or 6L:18D extra-short-day (ESD; panel d) cycles. Eyes (n = 5, no mark; n = 4, +; n = 3, *) were collected to measure *zCry1ab* mRNA levels (see Methods for details) at the indicated time points from ZT1. Each mRNA level was estimated as a relative value to the geometric mean of mRNA levels of *zβ-actin*, *zGapdh* and *zEf1α*. Error bars represent ± SD. Light and dark periods are indicated in blue and grey, respectively. Data were analyzed by two-way ANOVA (Supplementary Table S58) and Tukey-Kramer post-hoc tests (Supplementary Tables S59–S63).

The biphasic expression of *zCry1ab* in the eye (Fig. 4e) led us to hypothesize that *zCry1ab* expression is dependent upon day length. To explore this possibility, we entrained zebrafish to blue light/dark cycles of the different photoperiods and investigated the expression patterns of ocular *zCry1ab* at 2-h intervals (Fig. 5). Under all of the photoperiodic conditions examined, the evening peaks were more pronounced than the morning (shoulder) peaks. Notably, the expression profiles of *zCry1ab* changed depending on the photoperiod (interaction *p* < 0.01, Supplementary Table S58): both the morning and evening peaks advanced with the shortening of the light period.

**Figure 6:**
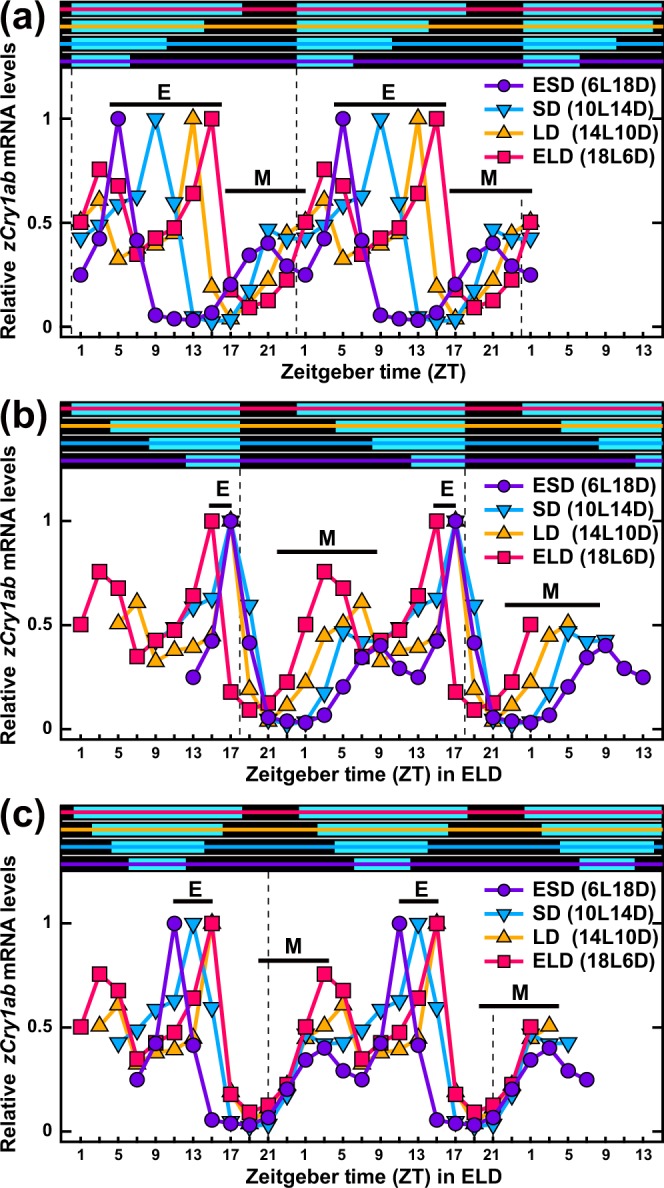
Double plots of 24-h profiles of relative *zCry1ab* expression levels in the eye under different photoperiods. Relative values of *zCry1ab* expression levels (Fig. 5; normalized by the peak) were double-plotted using ‘lights on’ (panel a), ‘lights off’ (panel b) or the middle of the night (panel c) as reference points. Data for two points at ZT1 (the start and end of sampling) were averaged. Evening peaks and increasing phases to morning (shoulder) peaks are indicated by E and M, respectively. Light conditions are indicated by bars with colored lines at the top of each panel.

In order to identify the underlying regulatory mechanisms, we compared *zCry1ab* profiles (relative mRNA levels) under the four photoperiodic conditions using different reference points (Fig. 6). When the profiles were plotted with ‘lights off’ as the reference point (Fig. 6b), the evening peaks were superimposed 1 h before lights off, except under ELD conditions (“E” in panel b), while the morning (shoulder) peaks diverged. Under ELD conditions (Figs. 5a and 6b), the evening peak occurred 3 h before lights off (ZT15), and the *zCry1ab* level decreased 1 h before lights off (ZT17). The fact that *zCry1ab* mRNA decreased during the light period refutes the hypothesis of dark-triggered mRNA downregulation around the end of the light period. In contrast to the evening peaks, the morning (shoulder) peaks seemed to be closer together when the profiles were plotted with midnight (broken lines in Fig. 6c) or noon as the reference points. In this plot, troughs and increasing phases coincided at midnight (“M” in Fig. 6c). These comparisons highlight the photoperiod-dependent changes in the duration of *zCry1ab* expression.

### Contributions of the light input pathway and circadian clock

**Figure 7:**
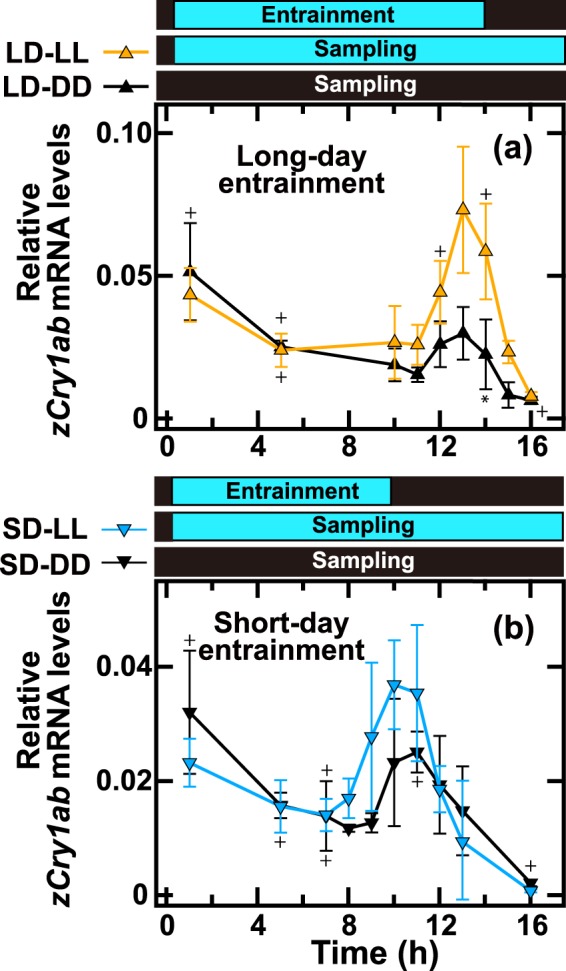
*zCry1ab* mRNA levels in the eye under constant light (LL) or dark (DD) conditions after entrainment to different photoperiods. (**a**) Zebrafish were entrained to a 14L:10D long-day cycle and kept in constant blue light (LL; yellow up-pointing triangles) or in the dark (DD; black up-pointing triangles). (**b**) Zebrafish were entrained to a 10L:14D short-day cycle and kept in constant blue light (LL; blue down-pointing triangles) or in the dark (DD; black down-pointing triangles). Eyes (n = 4, no mark; n = 3, +; n = 2, *) were collected to measure *zCry1ab* mRNA levels (see Methods for details) at the indicated time points from ZT0 on the last day. Each mRNA level was estimated as a relative value to the geometric mean of mRNA levels of *zβ-actin*, *zGapdh* and *zEf1α*. Data were analyzed by two-way ANOVA (Supplementary Tables S64 and S68) and Tukey-Kramer post-hoc tests (Supplementary Tables S65–S67 and S69–S71). Error bars represent ± SD.

*zCry1ab* mRNA level was maintained in the evening under constant dark condition (black square at ZT14 in Fig. 4e), implying that *zCry1ab* expression was regulated by not only the light signal but also the circadian clock in the evening. In order to validate this hypothesis, we investigated the evening levels of *zCry1ab* mRNA at 1-h intervals under blue light or in the dark after entrainment to the LD or SD conditions (Fig. 7). When the fish were kept under constant blue light conditions after entrainment to a LD condition (LD-LL, orange up-pointing triangles in Fig. 7a), the *zCry1ab* expression level increased from ZT11 and peaked at ZT13, before decreasing to a very low level at ZT16. This decrease in constant light confirmed that the decrease after the evening peak was not triggered by lights off, and instead suggested that downregulation was probably dependent on the circadian regulatory mechanism. In fact, a small peak was observed in the evening (12–14 h after ZT0) when the fish were kept under constant dark conditions after entrainment to a LD condition (LD-DD, black up-pointing triangles in Fig. 7a). The level of the peak at ZT13 in LD-LL was significantly higher than that in LD-DD (2.45-fold, *p* < 0.01, Light13 vs Dark13 in Supplementary Table S65), suggesting combinatorial regulation by both the circadian clock and photic upregulation to form the evening peak. We then investigated levels of *zCry1ab* transcripts after entrainment to short-day conditions (SD-LL and SD-DD, Fig. 7b, Supplementary Tables S68–S71). The evening peak was observed at ZT10-11 in SD-LL (blue down-pointing triangles in Fig. 7b), and a lower peak was observed at ZT11 under SD-DD conditions (black down-pointing triangles in Fig. 7b).

### Localization of the cell layer expressing *zCry1ab* mRNA in the evening by laser microdissection (LMD) and qRT-PCR

**Figure 8:**
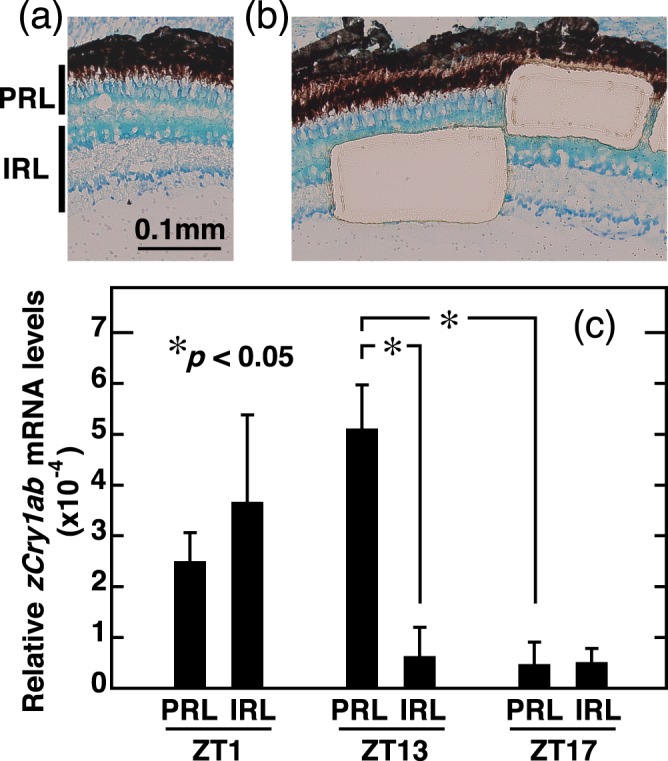
qRT-PCR analysis of *zCry1ab* mRNA levels in the zebrafish retinal cells captured by laser microdissection (LMD). Retinas were collected at ZT1 or ZT13 or ZT17 from zebrafish entrained to long-day cycles (14L10D). Photoreceptor cell layer (PRL) mainly containing outer nuclear layer (visual photoreceptors) and inner retinal layer (IRL) containing inner nuclear layer, inner plexiform layer and retinal ganglion cells were obtained from the retinal sections. (**a**) A retinal section (ZT1) showing PRL and IRL. (**b**) An example of retinal sections after the collection of PRL and IRL samples by LMD. (**c**) Levels of *zCry1ab* mRNA in PRL and IRL. The levels of *zCry1ab* mRNA and 18S rRNA were measured by qRT-PCR, and the *zCry1ab* mRNA levels relative to 18S rRNA levels were shown. Error bars represent ± SE. Data were analyzed by two-way ANOVA (Supplementary Table S72) and Tukey-Kramer post-hoc tests (Supplementary Table S73). Asterisks represent significant difference (*p* < 0.05).

The photoperiodic and biphasic expression of *zCry1ab* in the retina raised questions about its physiological importance and its transcriptional regulation mechanisms. To approach these questions, we next tried to identify the retinal cell layer, in which the retina-specific biphasic expression of *zCry1ab* occurs, by LMD followed by qRT-PCR (Fig. 8). We collected two retinal regions, photoreceptor layer (PRL) and inner retinal layer (IRL)(Fig. 8a,b); PRL dominantly contains visual photoreceptors and IRL contains inner nuclear layer, inner plexiform layer, and ganglion cell layer. The *zCry1ab* mRNA level in PRL at ZT13 was significantly higher than those in IRL at ZT13 and PRL at ZT17 (Fig. 8c; *p* < 0.05, Supplementary Tables S72 and S73). The *zCry1ab* mRNA levels in PRL well agreed with the profile obtained in the analyses of the eye (Fig. 5b), while those in IRL were rather consistent with *zCry1ab* mRNA profile in the brain (Fig. 4b).

## Discussion

The *zCry* genes studied were classified into two groups based on their mRNA expression profiles in five zebrafish tissues (Figs. 1 and 2): the morning group (*zCry1aa*, *zCry1ab* and *zCry2*) and the evening group (*zCry1ba*, *zCry1bb* and *zCry4*), which exhibited expression peaks in the morning and evening, respectively (Fig. 3). The daily expression peaks of all six *Cry* genes seemed to be conserved in most of the tissues examined, except for ocular *zCry1ab* and muscle *zCry4* (Fig. 3, Table 1), suggesting possible tissue-specific regulation mechanisms of *zCry1ab* and *zCry4* in the eye and muscle, respectively. The further examination of photic and circadian regulations of the *zCry* genes between the brain and eye suggested that not only *zCry1ab* and *zCry4* but also *zCry1ba* is likely regulated in a tissue-specific manner (Fig. 4g,j). The diurnal variation and/or light responsiveness of *zCry* mRNA expression in zebrafish cell lines and living animals have been reported by several groups so far (Table 1). Although some previous reports had different experimental conditions to the present study, such as sampling regime, tissues and light conditions, our results are mainly consistent with those obtained in previous studies. Together, they indicate that each zebrafish organ has light input and oscillation systems.

The divergent, or almost anti-phase, profiles of the morning and evening groups (Figs. 1–3) suggest that group-specific expression control occurred and there was functional divergence between the two groups. In fact, zCRYs exhibit varying repressive activity against CLOCK-BMAL complexes^2,6,14^, which could be explained by combinations of three CLOCKs and BMALs forming varying complexes. In mammals, E-box/E′-box, D-box and RRE are morning-time, day-time and night-time elements, respectively^18^ and CREB and AP-1 sites possibly function as light-responsive elements^28,29^. On the other hand, E-box and D-box are involved in light-dependent gene expression as well as circadian regulation in zebrafish^30–32^. Thus, *zCry* genes in the morning and evening groups may be differentially regulated by these elements. This hypothesis led us to searche for putative core clock and light-responsive elements (E-box/E′-box, D-box, RRE, CREB, and AP-1) in *zCry* genes (Supplementary Fig. S2, Supplementary Tables S74–S79). There is no clear difference between numbers of the putative elements in the morning- and evening-group genes, but clusters of E-box/E′-box and D-box elements were found in or near Exon 1 of the morning-group genes (Supplementary Fig. S2, red bars). On the other hand, the E-box/E′-box and D-box clusters were accompanied by RREs in the evening-group genes (Supplementary Fig. S2, red bars). Combinatorial regulations depending on the context of promoter/enhancer may occur in those clusters, and hence temporal and tissue-dependent changes in their regulatory activities should be further examined by genome-wide analyses such as ChIP-seq.

**Figure 9:**
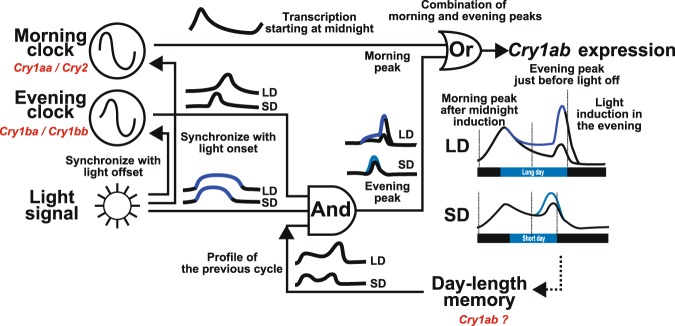
Model of a possible *zCry1ab* regulatory circuit in the zebrafish eye. The *zCry1ab* mRNA level was regulated by morning, evening, light and day length signals. Based on their mRNA expression patterns (Figs. 1–3), *Cry1aa/Cry2* and *Cry1ba/Cry1bb* were considered morning and evening oscillators, respectively. The morning signal induced the expression of *zCry1ab* at midnight, regardless of light and day length conditions. In addition to resetting the morning and evening clocks, the external light signal induced *zCry1ab* expression in the evening to form the evening peak, which may have been based on an integrated signal of light, day length and evening information. The morning and evening peaks were independent and additive. Shown on the right are schematic drawings of *zCry1ab* expression profiles under long-day and short-day cycles (modified from data shown in Figs. 4 and 7). Biological signal integration and regulation are represented using logic gates, with representative images of daily regulation patterns.

We found that the previously unreported unique expression properties of *zCry1ab* may be governed by both photoperiod- and tissue-specific regulation: *zCry1ab* expression peaks in the morning and evening, and the evening peak probably depends on phase-specific photic induction (Fig. 7). Weak rhythmic changes were observed around the projected time points, which corresponded with the end of the light period in the dark (LD-DD and SD-DD; Fig. 7a,b), indicating that the timing of the evening peak was synchronized with the internal clock, the phase of which was defined by the end of the light period in the previous cycle(s) (Fig. 9). The evening signal might trigger the phase-specific photoinduction of the *zCry1ab* promoter/enhancer region (Supplementary Fig. S2), in which light and circadian signals combinatorially regulate *zCry1ab* transcription. Such regulation may fine-tune the evening peak to occur just before lights off in a wide range of photoperiods (Figs. 5 and 6). In addition to the evening peak, *zCry1ab* transcription was probably tuned to start at midnight (Fig. 6c), which resulted in morning *zCry1ab* expression (between midnight and noon). It is unclear what mechanism underlies how the ocular cells anticipate midnight, so future studies should conduct transcriptome analyses of zebrafish eyes under different photoperiods in order to identify the mechanism involved.

Another important issue is whether both the morning and evening peaks originated from multiple oscillatory loops present in a single cell or different subsets of ocular cells. The present analysis of *zCry1ab* mRNA levels in the retinal photoreceptor and the other layers (Fig. 8) implied that the biphasic expression occurs exclusively in the photoreceptor cells. Although it is difficult to show the presence of dual loops in a single photoreceptor cell, analyses of cultured photoreceptor cells or a photoreceptor-derived cell line would resolve the issue.

*zCry1ab* activity around dusk may be particularly important, because photoperiodic responses are generally triggered by a light stimulus at dusk called the “photo-inducible phase”^1^. In Japanese quail (*Coturnix japonica*), which is a photoperiodic species, the photo-inducible phase occurs around ZT13-ZT14, and downstream photoperiodic responses, such as the induction of *Dio2* and thyroid hormones in the hypothalamic area, are well-characterised^1^. In the quail *pars tuberalis*, which is a key site for its seasonal responses, *Cry1* is induced by light in a photoinducible-phase-specific manner^33^. The upstream molecular mechanism that opens the temporal window of the photoinducible phase has not yet been identified in quail or any other animal, so the zebrafish eye is a suitable model for investigating photoperiodic gene expression mechanisms.

When compared with a strongly photoperiodic fish, medaka (*Oryzias latipes*)^34^, photoperiodic responses had been less characterised in zebrafish. Zebrafish are naturally found in northern and north-eastern India (~26.5°N, ~89.5°E), and their breeding season is reportedly between April and August^35^. The day length change in their natural habitat is approximately 10–14 h that is not so large as those in temperate zones but may be enough to trigger the photoperiodic responses, because some tropical or neotropical animals respond to changes in the day length less than 1 h^36,37^.

Based on the result of LMD followed by qRT-PCR (Fig. 8), we consider that the light signal received in the visual photoreceptors likely controls the photoperiodic expression of *zCry1ab*. Possibly, it regulates the photoperiod-responsive genes in the photoreceptor cells, thereby triggering the seasonal change in the retinal photoreceptor physiology relevant to vision. In the retina, visual sensitivity, retinomotor movement and melatonin synthesis may change with light and circadian time^38^. In the medaka, the mRNA expression of opsins in the retina changes with the environmental temperature as a seasonal response^39^. To the best of our knowledge, there have been few reports of the eye controlling photoperiodic responses, either locally or systemically, but our results suggest that the eye might play a role in photoperiodic responses, at least in the eye itself. In this regard, a recent paper reported that the neural network and intercellular synchronization in the mouse suprachiasmatic nucleus (SCN) are dependent upon day length^40^, which suggests that tissue-specific photoperiodic measurement occurs in neural tissues. Because *zCry1ab* is a potent inhibitor of E-box/E′-box-mediated circadian gene expression, it is possible that it plays a role in photoreceptor-specific physiological phenomena. In fact, mRNA levels of retinal *Opn4* decrease when zebrafish are transferred from 14L:10D to 18L:6D^41^. Future studies should analyze the downstream molecules of *zCry1ab* and the upstream regulatory mechanism of *Opn4*. The *in vitro* and *in vivo* functional characterization of *zCry1ab* and its promoter/enhancer are also required in order to ascertain how and why two peaks occur in the morning and evening.

## Methods

### Ethics statement

All experiments were conducted in accordance with the guidelines of Waseda University. All protocols were approved by the Committee for the Management of Biological Experiment at Waseda University, and experimental animal care was conducted with permission from the Committee for Animal Experimentation of the School of Science and Engineering, Waseda University (permission # WD15-060; 2015-A028; #2018-A104; #2019-A039).

### Animals

Wild-type zebrafish (EkkWill or commercial fish obtained from a local supplier) were maintained in stock tanks at 26–30 °C under 14L:10D long days and white light/dark cycles with lights on at 9:00 am. Fluorescent lamps (FHF32EX-N-HX-S, three-wavelength type, daylight-white, 32 W; Supplementary Fig. S1, black curve) were used as a light source, and the intensity was 5–20 μW cm^−2^ at water level.

### Collection of tissues during the white light, long-day cycle

Zebrafish (EkkWill line) were transferred from the stock tanks to another tank and fasted under the same cycles (14L:10D, lights on at 9:00 am) at 26 °C for 2 days before sampling. The light source was fluorescent lamps (FL20SS-EX-N/18-F, three-wavelength type, daylight-white, 18 W; Supplementary Fig. S1, black dotted curve) with an intensity of 210–240 μW cm^−2^ at water level. The fish were chilled on ice before sampling to avoid possible RNA synthesis or degradation. The brain, eye, pectoral fin, skin and skeletal muscle were taken at ZT1, ZT7, ZT15, ZT19 and ZT1 (n = 4 at each time point). During the light (ZT1 and ZT7) and dark (ZT15 and ZT19) periods, sampling was conducted under a white fluorescent lamp (240 μW cm^−2^; Supplementary Fig. S1, black curve) and a dim red light (>650 nm and <80 μW cm^−2^), respectively. The samples were kept in RNAlater^®^ (Ambion) for 24 h at 4 °C and stored at −80 °C until RNA extraction.

### Collection of eyes and brain under blue light conditions

Zebrafish obtained from a local supplier were entrained to a 14L:10D white light/dark cycle (lights on at 9:00 am) in stock tanks over 3 months. Subsequently, they were transferred to another tank the day before sampling. At ZT0 of the sampling day, the fish were exposed to blue light (λ_max_ = 462 nm, λ_1/2_ = 20 nm; Supplementary Fig. S1, blue curve) or kept in the dark for 14 h. The light intensities were set at 5.586 × 10^14^ photons cm^−2^ s^−1^ which was equivalent to 240 μW cm^−2^. We took three or four samples at ZT0 (start of the light treatment), ZT1 (1-h light treatment), ZT3 (3-h light treatment), ZT7 (midpoint of the light period in the previous LD cycle), ZT14 (end of the light treatment), ZT15 (1 h after the light treatment), ZT19 (midpoint of the dark period in the previous LD cycle) and ZT24 (start of the light period in the previous LD cycle). At each sample collection, the fish were chilled as described above and their heads were collected under white light (light conditions) or a dim red light (>650 nm) (dark conditions; ZT15, ZT19 and ZT24 for the light-treated fish and all time points for the control fish kept in the dark) and incubated in RNAlater^®^ (Ambion) for 24 h at 4 °C. The eyes and brain were taken from the head and homogenized in TRIzol reagent (Invitrogen).

### Collection of eyes under different photoperiods

Zebrafish obtained from a local supplier were entrained to a 14L:10D white light/dark cycle (lights on at 9:00 am) in stock tanks. Subsequently, they were entrained to 18L:6D extra-long-day (ELD), 14L:10D long-day (LD), 10L:14D short-day (SD) or 6L:18D extra-short-day (ESD) cycles under white light/dark cycles for 7 days. They were then transferred to another tank and kept under blue light (Supplementary Fig. S1, blue curve)/dark cycles for each photoperiod (lights on at 9:00 am) at 28 °C for 4 days until sampling. On the day of sampling, we took three to five fish for 13 points from ZT1 at 2-h intervals. At each sampling, the fish were chilled as described above. The eyes were collected and immediately homogenized in TRIzol reagent under white light (light conditions) or a dim red light (>650 nm) (dark conditions).

### Collection of eyes after entrainment to LD or SD photoperiod

For the long-day sampling, zebrafish were entrained to a 14L:10D white light/dark cycle (lights on at 9:00 am; for the light conditions, see Animals) for more than 10 days and then transferred to another tank and kept under 14L:10D blue light (240 μW cm^−2^; Supplementary Fig. S1, blue curve)/dark cycles at 28 °C for 4 days until sampling. For the short-day sampling, zebrafish were entrained to the 10L:14D white light/dark cycle (lights on at 9:00 am; for the light conditions, see Animals) for 10 days and then transferred to another tank and kept under 10L:14D blue light (240 μW cm^−2^; Supplementary Fig. S1, blue curve)/dark cycles at 28 °C for 4 days until sampling. At ZT0 on the sampling day, the fish were exposed to blue light (light conditions) or kept in the dark (dark conditions). We took three or four samples at each time point. For each sampling, the fish were chilled as described in above and the eyes were collected and treated as described immediately homogenized in TRIzol reagent under white light (light conditions) or a dim red light (>650 nm) (dark conditions).

### RNA extraction and cDNA synthesis

Total RNA was extracted using TRIzol reagent (Invitrogen) following the manufacturer’s instructions, then treated with RNase-free Recombinant DNase I (TakaraBio). We synthesized cDNA from 0.4 or 0.5 μg (experiments shown in Figs. 1, 2 and 4) or 1 μg (experiments shown in Figs. 5 and 7) of total RNA using High Capacity cDNA Reverse Transcription Kit (Applied Biosystems).

### Quantitative reverse transcription-polymerase chain reaction

For each primer pair (Table 2), we verified the amplification efficiency and accuracy of the target sequence by quantitative reverse transcription-polymerase chain reaction (qRT-PCR) using a dilution series of head cDNA (a mixture of ZT7 and ZT19). qRT-PCR except for the case using LMD samples (see below) was conducted using 2 × Fast SYBR^®^ Green Master Mix (Applied Biosystems). A StepOnePlus Real-Time PCR System (Applied Biosystems) was used according to the manufacturer’s instructions. The relative level of each mRNA was calculated using the 2^−ΔΔCT^ method. Each CT value was the mean of three to five biological replicates.

**Table 2:** Primers used in qPCR.

Gene	Primer name	Sequence (5′-3′)	Amplification efficiency (%)
*zGapdh*	zf_gapdh_qRT-PCR_F	GATACACGGAGCACCAGGTTGTG	92.3
	zf_gapdh_qRT-PCR_R	GCTGTAACCGAACTCATTGTCATACCATG	
*zβ-actin*	zf_bactin2_qRT-PCR_F	CCTGTATGCCAACACTGTATTGTCTGG	86.2
	zf_bactin2_qRT-PCR_R	CTCAGGTGGGGCAATGATCTTGATC	
*zEf1α*^#^	zf_elfa_qRT-PCR_F	CTTCTCAGGCTGACTGTGC	107.4
	zf_elfa_qRT-PCR_R	CCGCTAGCATTACCCTCC	
*zCry1aa*	zf_cry1a_qRT-PCR_F	CAACGGAGACTACATACGGCGATATC	107.4
	zf_cry1a_qRT-PCR_R	CCATGGGCATAGGGTAGTGGAC	
*zCry1ab*	zf_cry1b_qRT-PCR_F2	GCAGCCACAGCCAGGTTACTC	92.8
	zf_cry1b_qRT-PCR_R2	CTGTTGTTCGGTCTTCACCATGTCG	
*zCry1ba*	zf_cry2a_qRT-PCR_F3	CAATGTCTCGCATGACATGGCAG	103.2
	zf_cry2a_qRT-PCR_R2	CCGTTGTAACTTGTGCGAAGTGGAG	
*zCry1bb*	zf_cry2b_qRT-PCR_F2	GACACGAAACGACGCATCGTCTTA	104.4
	zf_cry2b_qRT-PCR_R	CAGTTCACTCTGAGTGGCGTACG	
*zCry2*	zf_cry3_qRT-PCR_F	ACGTGGGAGTCAACCGATGGAGAT	121.4
	zf_cry3_qRT-PCR_R	GAAGACATCTGTTGGCTGTCCGC	
*zCry4*	zf_cry4_qRT-PCR_F	CGCAGAACTCACGCGAGATGTAG	105.0
	zf_cry4_qRT-PCR_R	CTTGGAAGTGCACTGTGACTCCG	

^#^Reported in ref. ^42^.

### LMD followed by qRT-PCR

Heads of zebrafish (ZT1, ZT13, and ZT17 under 14L:10D blue light/dark cycle entrainment) were embedded in a 1:2 (vol/vol) mixture of optimal cutting temperature (OCT) embedding compound (Tissue-Tek, Sakura) and 0.1 M sodium phosphate buffer (pH 7.4) without fixation, and frozen using isopentane and liquid nitrogen. Tissues were cut at 20 μm onto membrane slides (PEN-Membrane, 2.0 μm, Leica) using a cryostat (CM1850, Leica). Sections were fixed with 70% ethanol for 30 sec and washed with diethyl pyrocarbonate (DEPC) water for 30 sec. Morphology was exposed with 0.05% toluidine blue (pH 4.1, Wako) for 30 sec and washed with DEPC water for 30 sec for two times and air-dried for 30 minutes. Then the sections were immediately subjected to LMD system (LMD6000, Leica) to collect two regions of the retina; photoreceptor layer (PRL) and inner retinal layer (IRL) including inner nuclear layer, inner plexiform layer and ganglion cell layer. Tissues of each region from three fish were pooled together (45,000 μm^2^–70,000 μm^2^).

Total RNA was extracted with the RNeasy Mini kit (Qiagen) and the On-column DNase digestion (RNase-free DNase set, Qiagen). cDNAs were synthesized with High Capacity cDNA Reverse Transcription Kit (Applied Biosystems) according to the manufacturer’s instructions. The total RNA was divided into four aliquots for triplicate RT reactions and one negative control reaction without RTase to confirm no amplification from possibly contaminating residual gDNA.

The PCR reaction mixture was prepared with cDNA and 2x TaqMan Fast Advanced Master Mix (Applied Biosystems), 200 nM TaqMan probe ([FAM] TCTGT TTGTG AGCTT GTCCA ACCAT [TAMRA]) and 300 nM primers for *zCry1ab* (Table 2). PCR cycling conditions were 50 °C for 2 min, 95 °C for 20 sec and 40 cycles of 95 °C for 1 sec and 60 °C for 20 sec. 18S rRNA was used as internal control (18S_F1, TCGCT AGTTG GCATC GTTTA TG; 18S_R1, CGGAG GTTCG AAGAC GATCA). A StepOnePlus Real-Time PCR System (Applied Biosystems) was used according to the manufacturer’s instructions.

### Statistical analyses

Statistical analyses of the qRT-PCR data were performed using R v. 3.3.2 and 3.5.2 (https://cran.r-project.org). We investigated differences in gene expression using one-way and two-way analyses of variance (ANOVA) and Tukey-Kramer post-hoc tests. Results were considered statistically significant if *p* < 0.05. The acrophases of the daily expression patterns were analyzed by CircWave (v.1.4) (http://www.euclock.org/results/item/circ-wave.html).

## Supplementary information

Supplemetary information.

## Data Availability

The data supporting the findings of this study are available within the paper and its Supplementary Information Files.
