# Supplementary material for: A photoperiodic time measurement served by the biphasic expression of Cryptochrome1ab in the zebrafish eye
Source: Sci Rep. 2020 Mar 19;10:5056. doi: 10.1038/s41598-020-61877-4 (PMC7081220; doi:10.1038/s41598-020-61877-4)
Supplement: Supplementary file 1 — Supplemetary information. [file 41598_2020_61877_MOESM1_ESM.pdf]

Supplementary Information for

A photoperiodic time measurement served by the biphasic expression of *Cryptochrome1ab* in the zebrafish eye

Keiko Okano, Yuya Saratani, Ayumi Tamasawa, Yosuke Shoji, Riko Toda, Toshiyuki Okano*

Author affiliations:
Department of Electrical Engineering and Bioscience, Graduate School of Advanced Science and Engineering, Waseda University, Wakamatsu-cho 2-2, Shinjuku-ku, Tokyo 162-8480, Japan

* ***Corresponding author:** okano@waseda.jp

Supplementary Figure S1

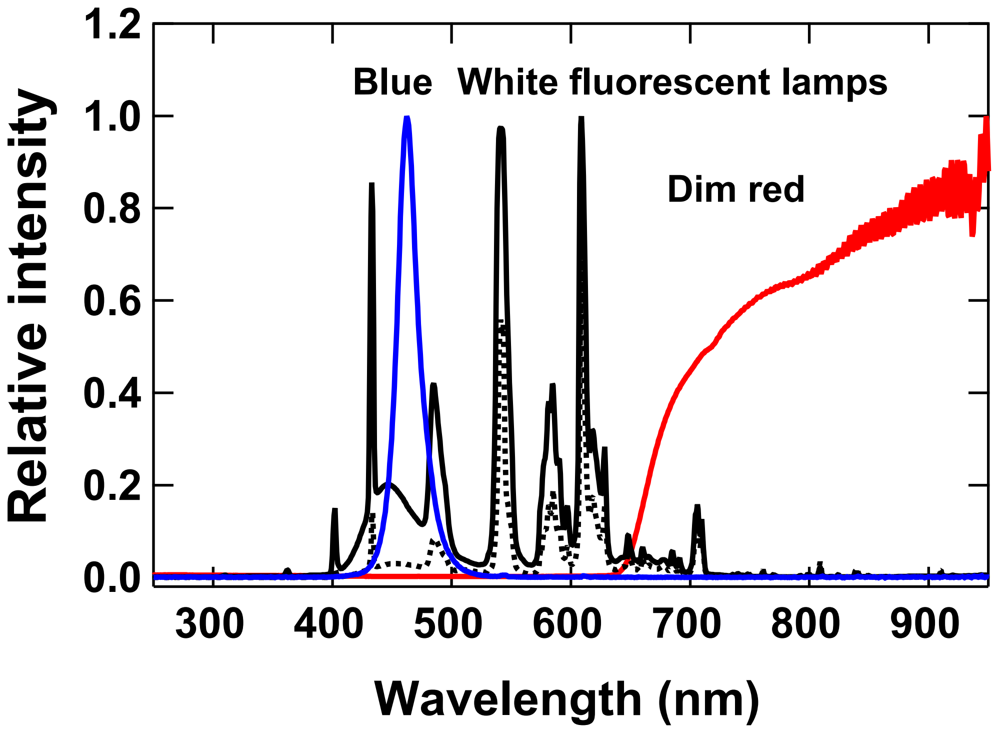

Supplementary Figure S1. Emission spectra of light-emitting diode (LED), fluorescent lamps and dim red light.

Philips Lumileds LXK2-PR14-Q00 (λ_{max} = 462 nm; $\lambda_{1/2}$ = 453 nm and 573 nm; blue curve) was used for blue light. The fluorescent lamps used were FHF32EX-N-HX-S (three-wavelength type, daylight-white, 32 W; black curve) and FL20SS-EX-N/18-F (three-wavelength type, daylight-white, 18 W; black dotted curve). Dim red light was produced using a combination of a safe light glass filter (No. 3, King) and a 5-W tungsten lamp (red curve). Spectra were measured using a photonic multichannel spectral analyser (Hamamatsu Photonics, Model PMA-11, type C7473-36).

Supplementary Figure S2

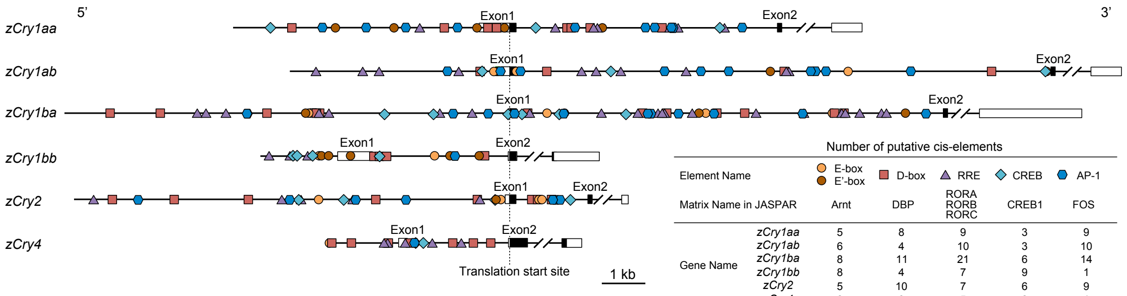

Number of putative cis-elements

Element Name		E-box / E'-box	D-box	RRE	CREB	AP-1
Matrix Name in JASPAR		Arnt	DBP	RORA RORB RORC	CREB1	FOS
Gene Name	*zCry1aa*	5	8	9	3	9
	zCry1ab	6	4	10	3	10
	zCry1ba	8	11	21	6	14
	zCry1bb	8	4	7	9	1
	zCry2	5	10	7	6	9
	zCry4	2	8	5	2	1

Supplementary Figure S2. Putative cis-acting elements present in zebrafish *Cry* genes. Positions of the E-box (CACGTG), E'-box (CACGTT, AACGTG), D-box, RRE, CREB, and AP-1 from the end or start of coding sequence of the upstream adjacent gene to exon2.

In case of *zCry1ba* and *zCry2*, 10 kb upstreams of the putative transcriptional start site (5' end of ESTs) were used, because the end or start of coding sequence of the upstream adjacent gene locates far from the putative transcriptional start sites. Sequences of *zCry* genes (*zCry1aa*, ID: 100003956; *zCry1ab*, ID: 554836; *zCry1ba*, ID: 573209; *zCry1bb*; ID: 83780, *zCry2*, Gene ID: 83774; *zCry4*, ID: 83775) were obtained from NCBI database and the elements were searched by using JASPAR (http://jaspar.genereg.net; Threshold, 90%). Captions and numbers of the putative elements are summarized in the inset. Open and filled boxes indicate coding and untranslated sequences, respectively. Locations of the putative elements are listed in Supplementary Tables S74-S79.

Description of additional supplementary items.

Supplementary Table S1: One-way analysis of variance (ANOVA) of the data shown in Figures 1 and 2.

Supplementary Tables S2-S31: Tukey-Kramer post-hoc tests after a one-way ANOVA of the data shown in Figures 1 and 2.

Supplementary Table S32: Cosinor analysis of the *Cry* gene expression levels shown in Figures 1 and 2.

Supplementary Table S33: Two-way ANOVA of the data shown in Figure 4.

Supplementary Tables S34-S45: Tukey-Kramer post-hoc tests after a two-way ANOVA of the data shown in Figure 4.

Supplementary Tables S46-S57: Selected results of a Tukey-Kramer post-hoc tests after a two-way ANOVA of the data shown in Figure 4.

Supplementary Table S58: Two-way ANOVA of the data shown in Figure 5.

Supplementary Table S59: Tukey-Kramer post-hoc test after a two-way ANOVA of the data shown in Figure 5.

Supplementary Tables S60-S63: Selected results of a Tukey-Kramer post-hoc test after a two-way ANOVA of the data shown in Figure 5.

Supplementary Table S64: Two-way ANOVA of the data shown in Figure 7a.

Supplementary Table S65: Tukey-Kramer post-hoc test after a two-way ANOVA of the data shown in Figure 7a.

Supplementary Tables S66 and S67: Selected results of a Tukey-Kramer post-hoc test after a two-way ANOVA of the data shown in Figure 7a.

Supplementary Table S68: Two-way ANOVA of the data shown in Figure 7b.

Supplementary Tables S69: Tukey-Kramer post-hoc test after a two-way ANOVA of the data shown in Figure 7b.

Supplementary Tables S70 and S71: Selected results of a Tukey-Kramer post-hoc test after a two-way ANOVA of the data shown in Figure 7b.

Supplementary Tables S72 and S73: Two-way ANOVA of the data shown in Figure 8 ("tableS72" Excel sheet) and Tukey-Kramer post-hoc test.

Supplementary Tables S74-S79: Results of searches for cis-acting elements in putative *zCry* promoter/enhancer regions shown in Supplementary Figure S2.

Table S1 One-way ANOVA for data shown in fig1&fig2

gene	tissue	degree of freedom	F value	*p* value	figure	post hoc test
cry1aa	brain	(4, 15)	34.91	1.96.E-07	figure 1a	table S2
	eyes	(4, 15)	40.17	7.60.E-08	figure 1b	table S3
	fin	(4, 15)	28.45	7.57.E-07	figure 1c	table S4
	skin	(4, 15)	30.11	5.21.E-07	figure 1d	table S5
	muscle	(4, 15)	20.94	3.50.E-05	figure 1e	table S6
cry1ab	brain	(4, 15)	18.66	1.09.E-05	figure 1f	table S7
	eyes	(4, 15)	22.18	3.73.E-06	figure 1g	table S8
	fin	(4, 15)	39.8	8.09.E-08	figure 1h	table S9
	skin	(4, 15)	4.438	1.45.E-02	figure 1i	table S10
	muscle	(4, 15)	5.1	8.50.E-03	figure 1j	table S11
cry1ba	brain	(4, 15)	29.27	6.27.E-07	figure 2a	table S12
	eyes	(4, 14)	241	9.72.E-13	figure 2b	table S13
	fin	(4, 13)	29.73	2.12.E-06	figure 2c	table S14
	skin	(4, 13)	23.8	7.00.E-06	figure 2d	table S15
	muscle	(4, 15)	6.558	2.94.E-03	figure 2e	table S16
cry1bb	brain	(4, 15)	39.58	8.41.E-08	figure 2f	table S17
	eyes	(4, 15)	63.63	3.15.E-09	figure 2g	table S18
	fin	(4, 15)	24.05	2.23.E-06	figure 2h	table S19
	skin	(4, 15)	24.82	1.83.E-06	figure 2i	table S20
	muscle	(4, 15)	13.1	8.73.E-05	figure 2j	table S21
cry2	brain	(4, 15)	34.36	2.17.E-07	figure 1k	table S22
	eyes	(4, 15)	46.19	2.93.E-08	figure 1l	table S23
	fin	(4, 15)	63.29	3.28.E-09	figure 1m	table S24
	skin	(4, 15)	36.63	1.42.E-07	figure 1n	table S25
	muscle	(4, 15)	5.028	8.99.E-03	figure 1o	table S26
cry4	brain	(4, 15)	31.16	4.16.E-07	figure 2k	table S27
	eyes	(4, 15)	50	1.69.E-08	figure 2l	table S28
	fin	(4, 15)	33.43	2.61.E-07	figure 2m	table S29
	skin	(4, 15)	12.2	1.30.E-04	figure 2n	table S30
	muscle	(4, 15)	8.63	8.02.E-04	figure 2o	table S31

tableS2 cry1aa_brain

	diff	lwr	upr	p adj	
ZT7 vs ZT19	0.038856319	0.027214819	0.050497819	2.96E-07	**
ZT19 vs ZT1	-0.030450154	-0.042091654	-0.018808654	6.64E-06	**
ZT7 vs ZT15	0.02826373	0.01662223	0.03990523	1.63E-05	**
ZT25 vs ZT19	0.026956258	0.015314758	0.038597758	2.86E-05	**
ZT15 vs ZT1	-0.019857566	-0.031499066	-0.008216065	0.000771026	**
ZT25 vs ZT15	0.016363669	0.004722169	0.028005169	0.004497883	**
ZT7 vs ZT25	0.011900061	0.000258561	0.023541561	0.043984889	*
ZT19 vs ZT15	-0.010592589	-0.022234089	0.001048911	0.083216707	
ZT7 vs ZT1	0.008406165	-0.003235335	0.020047665	0.221615758	
ZT25 vs ZT1	-0.003493897	-0.015135397	0.008147604	0.882129145	

tableS3 cry1aa_eyes

	diff	lwr	upr	p adj	
ZT19 vs ZT1	-0.028014958	-0.037005036	-0.019024881	7.27E-07	**
ZT7 vs ZT19	0.026918128	0.01792805	0.035908205	1.22E-06	**
ZT25 vs ZT19	0.02437519	0.015385112	0.033365268	4.26E-06	**
ZT15 vs ZT1	-0.021871928	-0.030862006	-0.01288185	1.59E-05	**
ZT7 vs ZT15	0.020775097	0.01178502	0.029765175	2.92E-05	**
ZT25 vs ZT15	0.01823216	0.009242082	0.027222237	0.000127893	**
ZT19 vs ZT15	-0.00614303	-0.015133108	0.002847047	0.265989804	
ZT25 vs ZT1	-0.003639768	-0.012629846	0.005350309	0.723597822	
ZT7 vs ZT25	0.002542938	-0.00644714	0.011533015	0.902239372	
ZT7 vs ZT1	-0.001096831	-0.010086908	0.007893247	0.995238997	

tableS4 cry1aa_fin

	diff	lwr	upr	p adj	
ZT7 vs ZT19	0.008094541	0.005083415	0.011105668	4.74E-06	**
ZT25 vs ZT19	0.007189844	0.004178717	0.010200971	1.99E-05	**
ZT19 vs ZT1	-0.007116645	-0.010127772	-0.004105518	2.25E-05	**
ZT7 vs ZT15	0.006316223	0.003305096	0.00932735	8.82E-05	**
ZT25 vs ZT15	0.005411525	0.002400398	0.008422652	0.000457763	**
ZT15 vs ZT1	-0.005338327	-0.008349453	-0.0023272	0.000525385	**
ZT19 vs ZT15	-0.001778318	-0.004789445	0.001232808	0.396655357	
ZT7 vs ZT1	0.000977897	-0.00203323	0.003989023	0.850063566	
ZT7 vs ZT25	0.000904698	-0.002106429	0.003915825	0.881728606	
ZT25 vs ZT1	7.32E-05	-0.002937928	0.003084326	0.999991874	

tableS5 cry1aa_skin

	diff	lwr	upr	p adj	
ZT7 vs ZT19	0.008034077	0.005298609	0.010769545	1.56E-06	**
ZT19 vs ZT1	-0.006875575	-0.009611042	-0.004140107	1.08E-05	**
ZT25 vs ZT19	0.006116555	0.003381087	0.008852023	4.28E-05	**
ZT7 vs ZT15	0.006047726	0.003312258	0.008783194	4.87E-05	**
ZT15 vs ZT1	-0.004889224	-0.007624691	-0.002153756	0.000483958	**
ZT25 vs ZT15	0.004130204	0.001394736	0.006865672	0.002419797	**
ZT19 vs ZT15	-0.001986351	-0.004721819	0.000749117	0.217318943	
ZT7 vs ZT25	0.001917522	-0.000817946	0.00465299	0.245007939	
ZT7 vs ZT1	0.001158503	-0.001576965	0.003893971	0.690764654	
ZT25 vs ZT1	-0.000759019	-0.003494487	0.001976448	0.908098554	

tableS6 cry1aa_muscle

	diff	lwr	upr	p adj	
ZT7 vs ZT19	0.078653933	0.048052021	0.109255844	8.22E-06	**
ZT7 vs ZT15	0.07299267	0.042390758	0.103594581	2.02E-05	**
ZT7 vs ZT25	0.057690083	0.027088172	0.088291995	0.000279503	**
ZT19 vs ZT1	-0.043123507	-0.073725419	-0.012521595	0.004403671	**
ZT15 vs ZT1	-0.037462244	-0.068064156	-0.006860333	0.013334174	*
ZT7 vs ZT1	0.035530426	0.004928514	0.066132337	0.019436228	*
ZT25 vs ZT1	-0.022159658	-0.052761569	0.008442254	0.219448348	
ZT25 vs ZT19	0.020963849	-0.009638062	0.051565761	0.263869736	
ZT25 vs ZT15	0.015302587	-0.015299325	0.045904498	0.551832651	
ZT19 vs ZT15	-0.005661263	-0.036263174	0.024940649	0.977338865	

tableS7 cry1ab_brain

	diff	lwr	upr	p adj	
ZT25 vs ZT19	0.01591103	0.008253654	0.023568405	9.79E-05	**
ZT25 vs ZT15	0.015044288	0.007386913	0.022701664	0.000180358	**
ZT19 vs ZT1	-0.014714381	-0.022371757	-0.007057006	0.000228477	**
ZT15 vs ZT1	-0.01384764	-0.021505015	-0.006190265	0.000429628	**
ZT7 vs ZT19	0.010836617	0.003179241	0.018493992	0.004248467	**
ZT7 vs ZT15	0.009969876	0.0023125	0.017627251	0.008365302	**
ZT7 vs ZT25	-0.005074413	-0.012731788	0.002582963	0.292097474	
ZT7 vs ZT1	-0.003877764	-0.01153514	0.003779611	0.540365111	
ZT25 vs ZT1	0.001196648	-0.006460727	0.008854024	0.987835367	
ZT19 vs ZT15	-0.000866741	-0.008524117	0.006790634	0.996432221	

tableS8 cry1ab_eyes

	diff	lwr	upr	p adj	
ZT19 vs ZT1	-0.010927787	-0.015124829	-0.006730746	7.02E-06	**
ZT25 vs ZT19	0.010629804	0.006432762	0.014826845	9.83E-06	**
ZT19 vs ZT15	-0.006496954	-0.010693995	-0.002299912	0.001932252	**
ZT7 vs ZT1	-0.006178762	-0.010375803	-0.00198172	0.003025873	**
ZT7 vs ZT25	-0.005880778	-0.01007782	-0.001683737	0.004619329	**
ZT7 vs ZT19	0.004749026	0.000551984	0.008946067	0.023163533	*
ZT15 vs ZT1	-0.004430834	-0.008627875	-0.000233792	0.036198236	*
ZT25 vs ZT15	0.00413285	-6.42E-05	0.008329892	0.054584824	
ZT7 vs ZT15	-0.001747928	-0.005944969	0.002449113	0.703265978	
ZT25 vs ZT1	-0.000297983	-0.004495025	0.003899058	0.999423346	

tableS9 cry1ab_fin

	diff	lwr	upr	p adj	
ZT19 vs ZT1	-0.002284621	-0.003009188	-0.001560053	6.24E-07	**
ZT15 vs ZT1	-0.002264562	-0.00298913	-0.001539995	7.00E-07	**
ZT25 vs ZT19	0.001759899	0.001035332	0.002484466	1.63E-05	**
ZT25 vs ZT15	0.001739841	0.001015273	0.002464408	1.86E-05	**
ZT7 vs ZT19	0.001511204	0.000786637	0.002235772	9.39E-05	**
ZT7 vs ZT15	0.001491146	0.000766578	0.002215713	0.000108892	**
ZT7 vs ZT1	-0.000773417	-0.001497984	-4.88E-05	0.033800624	*
ZT25 vs ZT1	-0.000524722	-0.001249289	0.000199846	0.219385245	
ZT7 vs ZT25	-0.000248695	-0.000973262	0.000475873	0.823629574	
ZT19 vs ZT15	-2.01E-05	-0.000744626	0.000704509	0.999986347	

tableS10 cry1ab_skin

	diff	lwr	upr	p adj	
ZT19 vs ZT1	-0.00274043	-0.004926927	-0.000553933	0.011197809	*
ZT25 vs ZT19	0.002166474	-2.00E-05	0.004352971	0.052699566	
ZT7 vs ZT19	0.001791853	-0.000394644	0.003978349	0.135624803	
ZT15 vs ZT1	-0.001629779	-0.003816276	0.000556718	0.197844725	
ZT19 vs ZT15	-0.001110651	-0.003297148	0.001075846	0.537576514	
ZT25 vs ZT15	0.001055823	-0.001130674	0.00324232	0.583019857	
ZT7 vs ZT1	-0.000948577	-0.003135074	0.001237919	0.672274577	
ZT7 vs ZT15	0.000681202	-0.001505295	0.002867698	0.867734989	
ZT25 vs ZT1	-0.000573956	-0.002760453	0.001612541	0.923321111	
ZT7 vs ZT25	-0.000374621	-0.002561118	0.001811875	0.982886341	

tableS11 cry1ab_muscle

	diff	lwr	upr	p adj	
ZT19 vs ZT1	-0.032045173	-0.060253286	-0.00383706	0.022552326	*
ZT15 vs ZT1	-0.029625745	-0.057833858	-0.001417632	0.037368086	*
ZT7 vs ZT19	0.027234004	-0.00097411	0.055442117	0.060912683	
ZT7 vs ZT15	0.024814575	-0.003393538	0.053022688	0.098255695	
ZT25 vs ZT1	-0.022582985	-0.050791098	0.005625128	0.149680135	
ZT7 vs ZT25	0.017771816	-0.010436298	0.045979929	0.336958823	
ZT25 vs ZT19	0.009462188	-0.018745925	0.037670301	0.83501311	
ZT25 vs ZT15	0.00704276	-0.021165354	0.035250873	0.935108464	
ZT7 vs ZT1	-0.00481117	-0.033019283	0.023396943	0.983169603	
ZT19 vs ZT15	-0.002419428	-0.030627542	0.025788685	0.99878756	

tableS12 cry1ba_brain

	diff	lwr	upr	p adj	
ZT15 vs ZT1	0.052723907	0.033449404	0.071998409	3.82E-06	**
ZT7 vs ZT1	0.04791678	0.028642278	0.067191282	1.23E-05	**
ZT25 vs ZT15	-0.04646693	-0.065741432	-0.027192427	1.78E-05	**
ZT7 vs ZT25	0.041659803	0.0223853	0.060934305	6.30E-05	**
ZT19 vs ZT15	-0.031362542	-0.050637044	-0.012088039	0.001215222	**
ZT7 vs ZT19	0.026555415	0.007280912	0.045829917	0.005313726	**
ZT19 vs ZT1	0.021361365	0.002086863	0.040635868	0.02657948	*
ZT25 vs ZT19	-0.015104388	-0.03437889	0.004170114	0.163290614	
ZT25 vs ZT1	0.006256977	-0.013017525	0.02553148	0.85025143	
ZT7 vs ZT15	-0.004807127	-0.024081629	0.014467376	0.935342318	

tableS13 cry1ba_eyes

	diff	lwr	upr	p adj	
ZT25 vs ZT15	-0.241968324	-0.270653921	-0.213282726	1.70E-12	**
ZT15 vs ZT1	0.241949687	0.210965701	0.272933673	6.68E-12	**
ZT19 vs ZT15	-0.219277653	-0.24796325	-0.190592056	9.49E-12	**
ZT7 vs ZT15	-0.195841103	-0.2245267	-0.167155506	4.98E-11	**
ZT7 vs ZT25	0.046127221	0.017441623	0.074812818	0.001505398	**
ZT7 vs ZT1	0.046108584	0.015124598	0.07709257	0.002973033	**
ZT7 vs ZT19	0.02343655	-0.005249047	0.052122147	0.135612845	
ZT25 vs ZT19	-0.02269067	-0.051376267	0.005994927	0.155032531	
ZT19 vs ZT1	0.022672034	-0.008311952	0.05365602	0.208205709	
ZT25 vs ZT1	-1.86E-05	-0.031002622	0.03096535	1	

tableS14 cry1ba_fin

	diff	lwr	upr	p adj	
ZT15 vs ZT1	0.019893982	0.012189406	0.027598558	1.56E-05	**
ZT25 vs ZT15	-0.019845039	-0.027549614	-0.012140463	1.60E-05	**
ZT7 vs ZT1	0.015458938	0.008325886	0.022591989	9.95E-05	**
ZT7 vs ZT25	0.015409994	0.008276943	0.022543045	0.000102785	**
ZT19 vs ZT15	-0.016253329	-0.024489867	-0.008016791	0.000254006	**
ZT7 vs ZT19	0.011818285	0.004113709	0.019522861	0.002515995	**
ZT7 vs ZT15	-0.004435044	-0.01213962	0.003269532	0.407283263	
ZT19 vs ZT1	0.003640652	-0.004063923	0.011345228	0.586986392	
ZT25 vs ZT19	-0.003591709	-0.011296285	0.004112867	0.598637968	
ZT25 vs ZT1	4.89E-05	-0.007084108	0.007181994	0.999999943	

tableS15 cry1ba_skin

	diff	lwr	upr	p adj	
ZT15 vs ZT1	0.020485615	0.012756399	0.028214831	1.17E-05	**
ZT25 vs ZT15	-0.020276363	-0.028005579	-0.012547147	1.31E-05	**
ZT7 vs ZT1	0.010134207	0.002978343	0.01729007	0.004817194	**
ZT7 vs ZT25	0.009924955	0.002769091	0.017080818	0.00567043	**
ZT7 vs ZT15	-0.010351408	-0.018080625	-0.002622192	0.007407788	**
ZT19 vs ZT15	-0.010906633	-0.019169513	-0.002643753	0.008256663	**
ZT19 vs ZT1	0.009578982	0.001849766	0.017308198	0.013011885	*
ZT25 vs ZT19	-0.00936973	-0.017098946	-0.001640514	0.015164148	*
ZT7 vs ZT19	0.000555225	-0.007173992	0.008284441	0.999336741	
ZT25 vs ZT1	0.000209252	-0.006946612	0.007365116	0.999981309	

tableS16 cry1ba_muscle

	diff	lwr	upr	p adj	
ZT25 vs ZT15	-0.027477778	-0.048585301	-0.006370254	0.008375152	**
ZT15 vs ZT1	0.025530413	0.004422889	0.046637936	0.014554199	*
ZT7 vs ZT25	0.023399941	0.002292418	0.044507464	0.026526784	*
ZT7 vs ZT1	0.021452576	0.000345053	0.042560099	0.045502435	*
ZT25 vs ZT19	-0.015453269	-0.036560792	0.005654255	0.211115466	
ZT19 vs ZT1	0.013505904	-0.00760162	0.034613427	0.322996448	
ZT19 vs ZT15	-0.012024509	-0.033132032	0.009083014	0.430524898	
ZT7 vs ZT19	0.007946672	-0.013160851	0.029054196	0.771597975	
ZT7 vs ZT15	-0.004077837	-0.02518536	0.017029687	0.973489832	
ZT25 vs ZT1	-0.001947365	-0.023054888	0.019160158	0.998387303	

tableS17 cry1bb_brain

	diff	lwr	upr	p adj	
ZT25 vs ZT15	-0.036981834	-0.048096618	-0.02586705	3.09E-07	**
ZT15 vs ZT1	0.036873659	0.025758875	0.047988443	3.21E-07	**
ZT19 vs ZT15	-0.028643157	-0.03975794	-0.017528373	7.96E-06	**
ZT7 vs ZT25	0.022770798	0.011656015	0.033885582	0.000114447	**
ZT7 vs ZT1	0.022662623	0.01154784	0.033777407	0.000120586	**
ZT7 vs ZT19	0.014432121	0.003317337	0.025546905	0.008544465	**
ZT7 vs ZT15	-0.014211036	-0.025325819	-0.003096252	0.009626595	**
ZT25 vs ZT19	-0.008338677	-0.019453461	0.002776106	0.19316217	
ZT19 vs ZT1	0.008230502	-0.002884281	0.019345286	0.202651556	
ZT25 vs ZT1	-0.000108175	-0.011222959	0.011006609	0.999999791	

tableS18 cry1bb_eyes

	diff	lwr	upr	p adj	
ZT25-ZT15	-0.041491961	-0.051269102	-0.031714819	1.16E-08	**
ZT15-ZT1	0.040831401	0.031054259	0.050608542	1.44E-08	**
ZT19-ZT15	-0.037457301	-0.047234443	-0.02768016	4.68E-08	**
ZT7-ZT25	0.020955248	0.011178107	0.030732389	6.93E-05	**
ZT7-ZT15	-0.020536713	-0.030313854	-0.010759571	8.68E-05	**
ZT7-ZT1	0.020294688	0.010517547	0.03007183	9.90E-05	**
ZT7-ZT19	0.016920589	0.007143447	0.02669773	0.000668491	**
ZT25-ZT19	-0.004034659	-0.013811801	0.005742482	0.709970304	
ZT19-ZT1	0.0033741	-0.006403042	0.013151241	0.820849723	
ZT25-ZT1	-0.00066056	-0.010437701	0.009116582	0.999525892	

tableS19 cry1bb_fin

	diff	lwr	upr	p adj	
ZT25 vs ZT15	-0.007565342	-0.010409689	-0.004720995	5.40E-06	**
ZT15 vs ZT1	0.007326491	0.004482144	0.010170838	8.01E-06	**
ZT19 vs ZT15	-0.006772103	-0.009616449	-0.003927756	2.06E-05	**
ZT7 vs ZT15	-0.00410662	-0.006950967	-0.001262273	0.003582475	**
ZT7 vs ZT25	0.003458723	0.000614376	0.006303069	0.01400294	*
ZT7 vs ZT1	0.003219871	0.000375524	0.006064218	0.023093963	*
ZT7 vs ZT19	0.002665483	-0.000178864	0.00550983	0.071491704	
ZT25 vs ZT19	-0.00079324	-0.003637587	0.002051107	0.906584874	
ZT19 vs ZT1	0.000554389	-0.002289958	0.003398735	0.972632749	
ZT25 vs ZT1	-0.000238851	-0.003083198	0.002605496	0.998884106	

tableS20 cry1bb_skin

	diff	lwr	upr	p adj	
ZT25 vs ZT15	-0.008473778	-0.01148732	-0.005460236	2.70E-06	**
ZT15 vs ZT1	0.008348136	0.005334594	0.011361678	3.26E-06	**
ZT7 vs ZT15	-0.005849551	-0.008863093	-0.002836009	0.000205238	**
ZT19 vs ZT15	-0.005405254	-0.008418796	-0.002391712	0.000466972	**
ZT25 vs ZT19	-0.003068524	-0.006082066	-5.50E-05	0.045005873	*
ZT19 vs ZT1	0.002942882	-7.07E-05	0.005956424	0.057187421	
ZT7 vs ZT25	0.002624228	-0.000389314	0.005637769	0.103122663	
ZT7 vs ZT1	0.002498585	-0.000514957	0.005512127	0.128931841	
ZT7 vs ZT19	-0.000444297	-0.003457839	0.002569245	0.990221387	
ZT25 vs ZT1	-0.000125642	-0.003139184	0.0028879	0.999930146	

tableS21 cry1bb_muscle

	diff	lwr	upr	p adj	
ZT25 vs ZT15	-0.016100481	-0.024105511	-0.008095452	0.000139985	**
ZT15 vs ZT1	0.014364345	0.006359315	0.022369374	0.000464968	**
ZT19 vs ZT15	-0.011244323	-0.019249353	-0.003239293	0.004524156	**
ZT7 vs ZT25	0.010540096	0.002535066	0.018545126	0.00766023	**
ZT7 vs ZT1	0.008803959	0.00079893	0.016808989	0.027941406	*
ZT7 vs ZT19	0.005683938	-0.002321092	0.013688967	0.234743534	
ZT7 vs ZT15	-0.005560385	-0.013565415	0.002444644	0.252428007	
ZT25 vs ZT19	-0.004856158	-0.012861188	0.003148871	0.371658391	
ZT19 vs ZT1	0.003120022	-0.004885008	0.011125051	0.749495896	
ZT25 vs ZT1	-0.001736137	-0.009741166	0.006268893	0.960001606	

tableS22 cry2_brain

	diff	lwr	upr	p adj	
ZT25 vs ZT15	0.066209683	0.042455877	0.089963488	3.02E-06	**
ZT15 vs ZT1	-0.063099661	-0.086853466	-0.039345856	5.49E-06	**
ZT25 vs ZT19	0.058624011	0.034870205	0.082377816	1.34E-05	**
ZT19 vs ZT1	-0.055513989	-0.079267794	-0.031760184	2.56E-05	**
ZT7 vs ZT25	-0.052258763	-0.076012568	-0.028504957	5.15E-05	**
ZT7 vs ZT1	-0.049148741	-0.072902547	-0.025394936	0.000102614	**
ZT7 vs ZT15	0.01395092	-0.009802886	0.037704725	0.40185662	
ZT19 vs ZT15	0.007585672	-0.016168133	0.031339477	0.857436738	
ZT7 vs ZT19	0.006365248	-0.017388558	0.030119053	0.917945033	
ZT25 vs ZT1	0.003110022	-0.020643784	0.026863827	0.993763709	

tableS23 cry2_eyes

	diff	lwr	upr	p adj	
ZT15 vs ZT1	-0.08000187	-0.103176176	-0.056827563	1.89E-07	**
ZT25 vs ZT15	0.07283	0.049655693	0.096004306	6.51E-07	**
ZT7 vs ZT1	-0.064625301	-0.087799608	-0.041450994	3.00E-06	**
ZT19 vs ZT1	-0.058442193	-0.0816165	-0.035267886	1.04E-05	**
ZT7 vs ZT25	-0.057453431	-0.080627738	-0.034279124	1.27E-05	**
ZT25 vs ZT19	0.051270323	0.028096016	0.07444463	4.83E-05	**
ZT19 vs ZT15	0.021559677	-0.00161463	0.044733984	0.074265294	
ZT7 vs ZT15	0.015376569	-0.007797738	0.038550875	0.291007064	
ZT25 vs ZT1	-0.00717187	-0.030346177	0.016002437	0.870410816	
ZT7 vs ZT19	-0.006183108	-0.029357415	0.016991199	0.91910201	

tableS24 cry2_fin

	diff	lwr	upr	p adj	
ZT15 vs ZT1	-0.018414115	-0.023257487	-0.013570743	5.19E-08	**
ZT19 vs ZT1	-0.017583923	-0.022427295	-0.012740551	9.66E-08	**
ZT25 vs ZT15	0.016586449	0.011743077	0.02142982	2.11E-07	**
ZT25 vs ZT19	0.015756256	0.010912884	0.020599628	4.15E-07	**
ZT7 vs ZT1	-0.014130766	-0.018974137	-0.009287394	1.69E-06	**
ZT7 vs ZT25	-0.012303099	-0.017146471	-0.007459727	9.48E-06	**
ZT7 vs ZT15	0.00428335	-0.000560022	0.009126721	0.095777161	
ZT7 vs ZT19	0.003453157	-0.001390215	0.008296529	0.231506605	
ZT25 vs ZT1	-0.001827667	-0.006671038	0.003015705	0.77017183	
ZT19 vs ZT15	0.000830192	-0.00401318	0.005673564	0.982859135	

tableS25 cry2_skin

	diff	lwr	upr	p adj	
ZT19 vs ZT1	-0.01846611	-0.02509095	-0.011841271	3.02E-06	**
ZT15 vs ZT1	-0.017224061	-0.023848901	-0.010599222	7.15E-06	**
ZT7 vs ZT1	-0.016873401	-0.023498241	-0.010248562	9.18E-06	**
ZT25 vs ZT19	0.016809683	0.010184843	0.023434522	9.61E-06	**
ZT25 vs ZT15	0.015567634	0.008942794	0.022192473	2.40E-05	**
ZT7 vs ZT25	-0.015216974	-0.021841813	-0.008592134	3.14E-05	**
ZT25 vs ZT1	-0.001656428	-0.008281267	0.004968412	0.93479228	
ZT7 vs ZT19	0.001592709	-0.005032131	0.008217549	0.942892934	
ZT19 vs ZT15	-0.001242049	-0.007866889	0.005382791	0.976213689	
ZT7 vs ZT15	0.00035066	-0.00627418	0.0069755	0.999819611	

tableS26 cry2_muscle

	diff	lwr	upr	p adj	
ZT19 vs ZT1	-0.065058114	-0.117926078	-0.01219015	0.01283334	*
ZT15 vs ZT1	-0.06287114	-0.115739104	-0.010003176	0.016433395	*
ZT7 vs ZT1	-0.054268233	-0.107136197	-0.001400269	0.042908578	*
ZT25 vs ZT1	-0.033494113	-0.086362077	0.019373851	0.33192199	
ZT25 vs ZT19	0.031564001	-0.021303963	0.084431965	0.386499554	
ZT25 vs ZT15	0.029377027	-0.023490937	0.082244991	0.453992549	
ZT7 vs ZT25	-0.02077412	-0.073642084	0.032093844	0.744095595	
ZT7 vs ZT19	0.010789881	-0.042078083	0.063657845	0.967732439	
ZT7 vs ZT15	0.008602907	-0.044265057	0.061470871	0.985857656	
ZT19 vs ZT15	-0.002186974	-0.055054938	0.05068099	0.999932293	

tableS27 cry4_brain

	diff	lwr	upr	p adj	
ZT15 vs ZT1	0.00664995	0.004447345	0.008852555	1.09E-06	**
ZT25 vs ZT15	-0.005952921	-0.008155525	-0.003750316	4.43E-06	**
ZT7 vs ZT1	0.005018425	0.00281582	0.007221029	3.45E-05	**
ZT7 vs ZT25	0.004321396	0.002118791	0.006524	0.000183071	**
ZT19 vs ZT15	-0.003326131	-0.005528735	-0.001123526	0.002416669	**
ZT19 vs ZT1	0.00332382	0.001121215	0.005526424	0.002431718	**
ZT25 vs ZT19	-0.00262679	-0.004829395	-0.000424186	0.016105829	*
ZT7 vs ZT19	0.001694605	-0.000507999	0.00389721	0.175555689	
ZT7 vs ZT15	-0.001631525	-0.00383413	0.000571079	0.202428064	
ZT25 vs ZT1	0.000697029	-0.001505575	0.002899634	0.861296656	

tableS28 cry4_eyes

	diff	lwr	upr	p adj	
ZT15 vs ZT1	0.003131162	0.002285064	0.00397726	7.47E-08	**
ZT25 vs ZT15	-0.003112185	-0.003958283	-0.002266086	8.11E-08	**
ZT19 vs ZT15	-0.002442534	-0.003288632	-0.001596436	1.94E-06	**
ZT7 vs ZT1	0.002028179	0.001182081	0.002874277	1.90E-05	**
ZT7 vs ZT25	0.002009202	0.001163103	0.0028553	2.12E-05	**
ZT7 vs ZT19	0.001339551	0.000493453	0.002185649	0.001570977	**
ZT7 vs ZT15	-0.001102983	-0.001949081	-0.000256885	0.008284712	**
ZT19 vs ZT1	0.000688628	-0.00015747	0.001534726	0.139677151	
ZT25 vs ZT19	-0.00066965	-0.001515748	0.000176448	0.156890537	
ZT25 vs ZT1	1.90E-05	-0.00082712	0.000865076	0.999994108	

tableS29 cry4_fin

	diff	lwr	upr	p adj	
ZT15 vs ZT1	0.000829185	0.000551359	0.00110701	1.27E-06	**
ZT25 vs ZT15	-0.000820495	-0.00109832	-0.000542669	1.45E-06	**
ZT7 vs ZT1	0.000618197	0.000340372	0.000896023	4.53E-05	**
ZT7 vs ZT25	0.000609508	0.000331682	0.000887333	5.32E-05	**
ZT19 vs ZT15	-0.000507675	-0.0007855	-0.00022985	0.000386222	**
ZT19 vs ZT1	0.00032151	4.37E-05	0.000599335	0.019882647	*
ZT25 vs ZT19	-0.00031282	-0.000590645	-3.50E-05	0.02393932	*
ZT7 vs ZT19	0.000296688	1.89E-05	0.000574513	0.033706786	*
ZT7 vs ZT15	-0.000210987	-0.000488813	6.68E-05	0.1845327	
ZT25 vs ZT1	8.69E-06	-0.000269136	0.000286515	0.999977779	

tableS30 cry4_skin

	diff	lwr	upr	p adj	
ZT15 vs ZT1	0.001411529	0.000648638	0.002174419	0.00033963	**
ZT25 vs ZT15	-0.001386295	-0.002149185	-0.000623404	0.000408938	**
ZT7 vs ZT1	0.000950705	0.000187814	0.001713595	0.011688221	*
ZT7 vs ZT25	0.000925471	0.00016258	0.001688361	0.014246892	*
ZT19 vs ZT1	0.000744848	-1.80E-05	0.001507739	0.057253689	
ZT25 vs ZT19	-0.000719614	-0.001482505	4.33E-05	0.069059337	
ZT19 vs ZT15	-0.000666681	-0.001429571	9.62E-05	0.101413857	
ZT7 vs ZT15	-0.000460824	-0.001223715	0.000302067	0.375625612	
ZT7 vs ZT19	0.000205857	-0.000557034	0.000968747	0.916057674	
ZT25 vs ZT1	2.52E-05	-0.000737657	0.000788125	0.999972227	

tableS31 cry4_muscle

	diff	lwr	upr	p adj	
ZT7 vs ZT19	0.012366622	0.004746682	0.019986563	0.001245492	**
ZT7 vs ZT25	0.0122462	0.004626259	0.01986614	0.001365807	**
ZT7 vs ZT15	0.010450872	0.002830931	0.018070812	0.005515614	**
ZT7 vs ZT1	0.008113144	0.000493204	0.015733085	0.034337895	*
ZT19 vs ZT1	-0.004253478	-0.011873418	0.003366462	0.449702928	
ZT25 vs ZT1	-0.004133055	-0.011752996	0.003486885	0.476720742	
ZT15 vs ZT1	-0.002337728	-0.009957668	0.005282213	0.873832634	
ZT19 vs ZT15	-0.001915751	-0.009535691	0.00570419	0.933576603	
ZT25 vs ZT15	-0.001795328	-0.009415268	0.005824613	0.946697244	
ZT25 vs ZT19	0.000120423	-0.007499518	0.007740363	0.999998546	

Table S32 Cosinor analysis of zCry gene expression levels in various tissues

gene	group	brain		eye		fin		skin		muscle	
		Acrophase	p-value	Acrophase	p-value	Acrophase	p-value	Acrophase	p-value	Acrophase	p-value
zCry1aa	morning	**5.9**	**< 0.001**	**4.8**	**< 0.001**	**5.1**	**< 0.001**	**5.5**	**< 0.001**	**6.9**	**< 0.001**
zCry1ab	morning	**3.3**	**< 0.001**	**(2.62)**	**(0.028)**	**3.4**	**< 0.001**	**4.1**	**0.020**	**5.3**	**0.012**
zCry1ba	evening	**11.4**	**< 0.001**	**13.1**	**< 0.001**	**11.3**	**< 0.001**	**13.0**	**< 0.001**	**12.0**	**< 0.001**
zCry1bb	evening	**11.9**	**< 0.001**	**11.9**	**< 0.001**	**12.0**	**< 0.001**	**13.4**	**< 0.001**	**11.9**	**< 0.001**
zCry2	morning	**1.1**	**< 0.001**	**0.5**	**< 0.001**	**1.3**	**< 0.001**	**0.9**	**< 0.001**	**1.4**	**0.016**
zCry4	evening	**12.1**	**< 0.001**	**11.7**	**< 0.001**	**11.9**	**< 0.001**	**12.5**	**< 0.001**	**8.2**	**< 0.001**

Table S33 Two-way ANOVA for data shown in fig4

gene	tissue	ZT vs light condition			light condition			ZT			figure	post hoc test
		degree of freedom	F value	*p* value	degree of freedom	F value	*p* value	degree of freedom	F value	*p* value		
cry1aa	brain	(7, 44)	4.19	1.29E-03	(1, 44)	47.08	1.82E-08	(7, 44)	23.35	7.14E-13	figure 4a	table S34
	eye	(7, 47)	4.89	3.33E-04	(1, 47)	20.00	4.88E-05	(7, 47)	25.95	3.98E-14	figure 4d	table S35
cry1ab	brain	(7, 44)	2.28	4.50E-02	(1, 44)	0.74	3.96E-01	(7, 44)	23.96	4.63E-13	figure 4b	table S36
	eye	(7, 47)	2.33	4.00E-02	(1, 47)	18.76	7.74E-05	(7, 47)	21.94	7.90E-13	figure 4e	table S37
cry1ba	brain	(7, 41)	2.35	4.12E-02	(1, 41)	5.21	2.77E-02	(7, 41)	12.32	2.32E-08	figure 4g	table S38
	eye	(7, 46)	15.34	3.67E-10	(1, 46)	48.98	9.17E-09	(7, 46)	89.09	< 2e-16	figure 4j	table S39
cry1bb	brain	(7, 43)	8.55	1.57E-06	(1, 43)	59.36	1.25E-09	(7, 43)	25.16	2.91E-13	figure 4h	table S40
	eye	(7, 45)	20.00	6.86E-12	(1, 45)	101.94	3.85E-13	(7, 45)	74.64	< 2e-16	figure 4k	table S41
cry2	brain	(7, 44)	0.80	5.93E-01	(1, 44)	1.79	1.88E-01	(7, 44)	8.91	8.60E-07	figure 4c	table S42
	eye	(7, 47)	2.24	4.71E-02	(1, 47)	5.14	2.80E-02	(7, 47)	37.06	<2e-16	figure 4f	table S43
cry4	brain	(7, 44)	0.58	7.70E-01	(1, 44)	0.16	6.94E-01	(7, 44)	14.42	1.42E-09	figure 4i	table S44
	eye	(7, 47)	4.31	9.32E-04	(1, 47)	6.14	1.69E-02	(7, 47)	54.36	< 2e-16	figure 4l	table S45

tableS34	brain_cry1aa				
	diff	lwr	upr	p adj	
D15-B03	-0.558017734	-0.751859175	-0.364176292	2.08E-11	**
D14-B03	-0.554637773	-0.748479215	-0.360796332	2.49E-11	**
B19-B03	-0.579065423	-0.78843811	-0.369692737	7.02E-11	**
D24-B03	-0.519605778	-0.71344722	-0.325764337	1.81E-10	**
D19-B03	-0.555403588	-0.764776274	-0.346030902	2.48E-10	**
D07-B03	-0.491173637	-0.685015079	-0.297332196	9.59E-10	**
B14-B03	-0.485221871	-0.679063312	-0.291380429	1.37E-09	**
B15-B03	-0.447448405	-0.641289846	-0.253606964	1.33E-08	**
D01-B03	-0.387103817	-0.580945258	-0.193262375	5.55E-07	**
D15-B01	-0.368398814	-0.562240255	-0.174557372	1.79E-06	**
D14-B01	-0.365018853	-0.558860295	-0.171177412	2.21E-06	**
B19-B01	-0.389446503	-0.59881919	-0.180073817	2.92E-06	**
D00-B03	-0.355174488	-0.54901593	-0.161333047	4.09E-06	**
B07-B03	-0.354187852	-0.548029294	-0.160346411	4.35E-06	**
D19-B01	-0.365784668	-0.575157354	-0.156411982	1.15E-05	**
D24-B01	-0.329986858	-0.5238283	-0.136145417	1.98E-05	**
B24-B03	-0.321811426	-0.515652867	-0.127969985	3.29E-05	**
D03-B03	-0.347549066	-0.556921753	-0.13817638	3.30E-05	**
B03-B00	0.345054023	0.135681337	0.55442671	3.81E-05	**
D07-B01	-0.301554717	-0.495396159	-0.107713276	0.000115116	**
B14-B01	-0.295602951	-0.489444392	-0.101761509	0.000165837	**
B15-B01	-0.257829485	-0.451670926	-0.063988044	0.001596535	**
B24-B19	0.257253997	0.047881311	0.466626684	0.004923753	**
D15-B24	-0.236206308	-0.430047749	-0.042364866	0.005495663	**
D14-B24	-0.232826347	-0.426667789	-0.038984906	0.006630851	**
D19-B24	-0.233592162	-0.442964848	-0.024219476	0.016212782	*
B19-B07	-0.224877571	-0.434250257	-0.015504885	0.024617735	*
D00-B19	0.223890935	0.014518249	0.433263621	0.025788431	*
D15-B07	-0.203829881	-0.397671323	-0.00998844	0.030689865	*
D15-D00	-0.202843245	-0.396684687	-0.009001804	0.032238951	*
B19-B00	-0.2340114	-0.457840217	-0.010182583	0.032534671	*
D03-B19	0.231516357	0.00768754	0.455345174	0.036208531	*
D14-B07	-0.200449921	-0.394291362	-0.00660848	0.036296262	*
D14-D00	-0.199463285	-0.393304726	-0.005621844	0.038099419	*
D24-B24	-0.197794352	-0.391635794	-0.003952911	0.04133426	*
D01-B01	-0.197484897	-0.391326338	-0.003643455	0.041960526	*
D15-B00	-0.21296371	-0.422336396	-0.003591024	0.04261463	*
D15-D03	-0.210468667	-0.419841353	-0.001095981	0.047633396	*
D14-B00	-0.20958375	-0.418956436	-0.000211064	0.049536268	*
D14-D03	-0.207088707	-0.416461393	0.002283979	0.055270216	
B03-B01	0.18961892	-0.004222521	0.383460361	0.06100651	
D19-B07	-0.201215736	-0.410588422	0.00815695	0.071130802	
D19-D00	-0.2002291	-0.409601786	0.009143586	0.074151736	
D19-B00	-0.210349565	-0.434178381	0.013479252	0.085653895	
D19-D03	-0.207854522	-0.431683338	0.015974295	0.094231247	
D01-B19	0.191961607	-0.01741108	0.401334293	0.104080628	
D15-D01	-0.170913917	-0.364755358	0.022927525	0.138596974	
D07-B24	-0.169362211	-0.363203653	0.02447923	0.147631277	
D14-D01	-0.167533957	-0.361375398	0.026307485	0.158866298	
D00-B01	-0.165555568	-0.35939701	0.028285873	0.17176309	
D24-B07	-0.165417926	-0.359259367	0.028423515	0.172689451	
B07-B01	-0.164568932	-0.358410374	0.029272509	0.178488025	
D24-D00	-0.16443129	-0.358272731	0.029410151	0.179441897	
B24-B14	0.163410445	-0.030430997	0.357251886	0.186637328	
D24-B00	-0.174551755	-0.383924441	0.034820931	0.199900517	
D24-D03	-0.172056712	-0.381429398	0.037315974	0.217849912	
D19-D01	-0.168299771	-0.377672458	0.041072915	0.247005429	
D03-B01	-0.157930146	-0.367302833	0.05144254	0.340567273	
B01-B00	0.155435103	-0.053937583	0.36480779	0.36577768	
D07-B07	-0.136985785	-0.330827226	0.056855656	0.446537895	
D07-D00	-0.135999149	-0.32984059	0.057842292	0.458593345	
D07-B00	-0.146119614	-0.3554923	0.063253072	0.467443443	
D07-D03	-0.143624571	-0.352997257	0.065748115	0.496233427	
D24-D01	-0.132501962	-0.326343403	0.06133948	0.502128887	
B24-B01	-0.132192506	-0.326033947	0.061648935	0.506031432	
B14-B07	-0.131034018	-0.32487546	0.062807423	0.520697406	
D00-B14	0.130047382	-0.063794059	0.323888824	0.533247326	
B14-B00	-0.140167847	-0.349540533	0.069204839	0.536781023	
D03-B14	0.137672804	-0.071699882	0.34704549	0.566323258	
B24-B15	0.125636979	-0.068204462	0.31947842	0.589683872	
B19-B15	-0.131617018	-0.340989705	0.077755668	0.637890387	
D15-B15	-0.110569329	-0.30441077	0.083272113	0.772711368	
D14-B15	-0.107189368	-0.30103081	0.086652073	0.80841606	
D07-D01	-0.104069821	-0.297911262	0.089771621	0.838768019	
D19-B15	-0.107955183	-0.317327869	0.101417503	0.874630462	
D01-B14	0.098118054	-0.095723388	0.291959495	0.888903234	
B15-B00	-0.102394382	-0.311767068	0.106978305	0.912167664	
B15-B07	-0.093260553	-0.287101994	0.100580889	0.92179516	
D03-B15	0.099899339	-0.109473348	0.309272025	0.9263528	
D00-B15	0.092273917	-0.101567525	0.286115358	0.927589771	
B19-B14	-0.093843553	-0.303216239	0.115529133	0.954185495	
D07-B19	0.087891786	-0.1214809	0.297264472	0.973279943	
D15-B14	-0.072795863	-0.266637304	0.121045578	0.990251221	
D24-B15	-0.072157373	-0.265998815	0.121684068	0.99104027	
D14-B14	-0.069415903	-0.263257344	0.124425539	0.993864988	
D15-D07	-0.066844096	-0.260685538	0.126997345	0.995807594	
D01-B24	-0.065292391	-0.259133832	0.128549051	0.996710122	
D19-B14	-0.070181718	-0.279554404	0.139190969	0.996872796	
D14-D07	-0.063464136	-0.257305577	0.130377305	0.997559433	
D01-B15	0.060344588	-0.133496853	0.25418603	0.998583637	
D19-D07	-0.064229951	-0.273602637	0.145142735	0.998795126	
D24-B19	0.059459645	-0.149913041	0.268832331	0.999496767	
D07-B15	-0.043725232	-0.237566674	0.150116209	0.999969427	
D01-B00	-0.042049793	-0.25142248	0.167322893	0.999993193	
D24-D15	0.038411955	-0.155429486	0.232253397	0.999994295	
B15-B14	0.037773465	-0.156067976	0.231614907	0.999995428	
D03-D01	0.03955475	-0.169817936	0.248927437	0.999996973	
D24-D14	0.035031995	-0.158809446	0.228873436	0.99999833	
D24-B14	-0.034383908	-0.228225349	0.159457534	0.999998702	
D00-B24	-0.033363062	-0.227204504	0.160478379	0.999999138	
D24-D19	0.03579781	-0.173574876	0.245170496	0.999999212	
D01-B07	-0.032915964	-0.226757406	0.160925477	0.999999283	
B24-B07	0.032376426	-0.161465015	0.226217868	0.999999428	
D01-D00	-0.031929329	-0.22577077	0.161912113	0.999999527	
D24-D07	-0.028432141	-0.222273582	0.1654093	0.999999905	
D03-B24	-0.02573764	-0.235110327	0.183635046	0.999999992	
D14-B19	0.02442765	-0.184945036	0.233800336	0.999999996	
B24-B00	0.023242597	-0.186130089	0.232615284	0.999999998	
D19-B19	0.023661835	-0.200166981	0.247490652	0.999999999	
D15-B19	0.02104769	-0.188324996	0.230420376	1	
B07-B00	-0.009133829	-0.218506515	0.200238857	1	
D00-B00	-0.010120465	-0.219493151	0.199252221	1	
D03-B00	-0.002495043	-0.22632386	0.221333774	1	
D00-B07	-0.000986636	-0.194828077	0.192854805	1	
D03-B07	0.006638786	-0.2027339	0.216011472	1	
D07-B14	-0.005951767	-0.199793208	0.187889675	1	
D03-D00	0.007625422	-0.201747264	0.216998108	1	
D15-D14	-0.00337996	-0.197221402	0.190461481	1	
D19-D14	-0.000765815	-0.210138501	0.208606871	1	
D19-D15	0.002614145	-0.206758541	0.211986832	1	
	diff	lwr	upr	p adj	

tableS35	eye_cry1aa				
	diff	lwr	upr	p adj	
B19-B03	-0.116661029	-0.161109542	-0.072212516	1.94E-10	**
D15-B03	-0.113525247	-0.15797376	-0.069076734	4.53E-10	**
D14-B03	-0.110928781	-0.155377294	-0.066480268	9.14E-10	**
D19-B03	-0.107756211	-0.152204724	-0.063307698	2.17E-09	**
B15-B03	-0.103314009	-0.147762522	-0.058865496	7.35E-09	**
B19-B01	-0.097127537	-0.14157605	-0.052679024	4.09E-08	**
D15-B01	-0.093991755	-0.138440268	-0.049543242	9.82E-08	**
D14-B01	-0.091395289	-0.135843802	-0.046946776	2.03E-07	**
D07-B03	-0.090419732	-0.134868245	-0.045971219	2.67E-07	**
D19-B01	-0.08822272	-0.132671233	-0.043774206	4.96E-07	**
B14-B03	-0.084371982	-0.128820495	-0.039923469	1.47E-06	**
B15-B01	-0.083780517	-0.12822903	-0.039332004	1.73E-06	**
D24-B03	-0.082955858	-0.127404371	-0.038507344	2.19E-06	**
B03-B00	0.072170734	0.027722221	0.116619247	4.48E-05	**
D00-B19	0.071593623	0.02714511	0.116042136	5.25E-05	**
D07-B01	-0.07088624	-0.115334753	-0.026437727	6.39E-05	**
D15-D00	-0.068457841	-0.112906354	-0.024009327	0.000124658	**
D14-D00	-0.065861375	-0.110309888	-0.021412862	0.000252924	**
B24-B19	0.064900531	0.020452018	0.109349044	0.00032792	**
B14-B01	-0.06483849	-0.109287003	-0.020389977	0.00033345	**
D24-B01	-0.063422366	-0.107870879	-0.018973853	0.000487697	**
D19-D00	-0.062688805	-0.107137318	-0.018240292	0.000593138	**
D01-B19	0.062060331	0.017611818	0.106508844	0.000700936	**
D15-B24	-0.061764749	-0.106213262	-0.017316235	0.000758028	**
D14-B24	-0.059168283	-0.103616796	-0.01471977	0.001497562	**
D15-D01	-0.058924549	-0.103373062	-0.014476036	0.001595304	**
D03-B03	-0.05833121	-0.102779723	-0.013882696	0.001859759	**
D03-B19	0.05832982	0.013881306	0.102778333	0.001860426	**
D00-B15	0.058246603	0.01379809	0.102695116	0.001900764	**
D14-D01	-0.056328083	-0.100776596	-0.01187957	0.003102918	**
D19-B24	-0.055995713	-0.100444226	-0.0115472	0.003374809	**
B19-B07	-0.059736239	-0.107746121	-0.011726358	0.004014768	**
D15-D03	-0.055194037	-0.09964255	-0.010745524	0.004127866	**
D01-B03	-0.054600698	-0.099049211	-0.010152185	0.004786162	**
D19-D01	-0.053155513	-0.097604026	-0.008707	0.006834275	**
B07-B03	-0.05692479	-0.104934671	-0.008914908	0.007632564	**
B01-B00	0.052637243	0.008188729	0.097085756	0.007753762	**
D14-D03	-0.052597572	-0.097046085	-0.008149058	0.007828773	**
D15-B07	-0.056600457	-0.104610338	-0.008590576	0.008209029	**
B24-B03	-0.051760498	-0.096209011	-0.007311985	0.009580993	**
B24-B15	0.051553511	0.007104998	0.096002024	0.010068101	*
D14-B07	-0.054003991	-0.102013873	-0.00599411	0.014546425	*
D19-D03	-0.049425002	-0.093873515	-0.004976489	0.016621507	*
D01-B15	0.048713311	0.004264798	0.093161824	0.019581772	*
D19-B07	-0.050831422	-0.098841303	-0.00282154	0.028427228	*
D07-D00	-0.045352326	-0.089800839	-0.000903812	0.041275383	*
D00-B03	-0.045067406	-0.089515919	-0.000618893	0.043866201	*
D03-B15	0.0449828	0.000534286	0.089431313	0.044663054	*
B19-B00	-0.044490295	-0.088938808	-4.18E-05	0.049563109	*
B15-B07	-0.046389219	-0.094399101	0.001620662	0.068119098	
D15-B00	-0.041354512	-0.085803026	0.003094001	0.093345799	
D00-B14	0.039304576	-0.005143937	0.083753089	0.13692262	
D03-B01	-0.038797718	-0.083246231	0.005650795	0.149906365	
D14-B00	-0.038758047	-0.08320656	0.005690466	0.150962196	
D07-B24	-0.038659234	-0.083107747	0.00578928	0.153617453	
D24-D00	-0.037888451	-0.082336964	0.006560062	0.175595866	
D07-D01	-0.035819034	-0.080267547	0.008629479	0.246205381	
D19-B00	-0.035585477	-0.08003399	0.008863036	0.255262513	
D01-B01	-0.035067206	-0.079515719	0.009381307	0.276147083	
B07-B01	-0.037391298	-0.085401179	0.010618583	0.295131612	
D24-B19	0.033705172	-0.010743342	0.078153685	0.336057077	
B24-B14	0.032611484	-0.011837029	0.077059997	0.389039083	
B19-B14	-0.032289047	-0.07673756	0.012159466	0.405393732	
B24-B01	-0.032227007	-0.07667552	0.012221507	0.408575526	
D07-D03	-0.032088522	-0.076537035	0.012359991	0.41571692	
D24-B24	-0.031195359	-0.075643872	0.013253154	0.462952189	
B15-B00	-0.031143275	-0.075591788	0.013305238	0.46576263	
D07-B07	-0.033494942	-0.081504823	0.014514939	0.472962623	
D24-D15	0.030569389	-0.013879124	0.075017902	0.497059509	
D01-B14	0.029771284	-0.014677229	0.074219797	0.541343993	
D15-B14	-0.029153265	-0.073601778	0.015295248	0.575944694	
D24-D01	-0.02835516	-0.072803673	0.016093354	0.620561259	
D24-D14	0.027972924	-0.01647559	0.072421437	0.641744337	
D00-B00	0.027103328	-0.017345185	0.071551841	0.689043216	
D14-B14	-0.026556799	-0.071005312	0.017891714	0.717857648	
D07-B19	0.026241297	-0.018207216	0.07068981	0.734079641	
D03-B14	0.026040773	-0.018407741	0.070489286	0.744212627	
D00-B01	-0.025533915	-0.069982428	0.018914599	0.76914594	
B14-B07	-0.027447192	-0.075457074	0.020562689	0.77502703	
D24-D19	0.024800354	-0.019648159	0.069248867	0.803291321	
D24-D03	-0.024624648	-0.069073161	0.019823865	0.811095352	
D24-B07	-0.026031068	-0.074040949	0.021978813	0.833463667	
D19-B14	-0.023384229	-0.067832742	0.021064284	0.861606866	
D15-D07	-0.023105515	-0.067554028	0.021342998	0.871784833	
D14-D07	-0.020509049	-0.064957562	0.023939464	0.944831109	
B24-B00	0.020410236	-0.024038277	0.064858749	0.946850903	
D24-B15	0.020358151	-0.024090362	0.064806665	0.947894389	
B03-B01	0.019533492	-0.024915021	0.063982005	0.962540276	
B15-B14	-0.018942027	-0.06339054	0.025506486	0.971009374	
D07-B00	-0.018248997	-0.062697511	0.026199516	0.979006942	
D01-B00	0.017570036	-0.026878477	0.062018549	0.985080197	
D19-D07	-0.01733648	-0.061784993	0.027112034	0.986815011	
B07-B00	0.015245945	-0.032763937	0.063255826	0.998357492	
D03-B00	0.013839525	-0.030608988	0.058288038	0.998676824	
B19-B15	-0.01334702	-0.057795533	0.031101493	0.99911674	
D03-D00	-0.013263803	-0.057712316	0.03118471	0.999176962	
D07-B15	0.012894277	-0.031554236	0.05734279	0.999403582	
B14-B00	-0.012201248	-0.056649761	0.032247265	0.999686602	
D00-B07	0.011857383	-0.036152498	0.059867265	0.999912599	
D24-B00	-0.010785123	-0.055233636	0.03366339	0.999929819	
D15-B15	-0.010211238	-0.054659751	0.034237275	0.999964676	
D01-D00	-0.009533292	-0.053981805	0.034915221	0.999985353	
D19-B19	0.008904818	-0.035543695	0.053353331	0.999993994	
D14-B15	-0.007614772	-0.052063285	0.036833741	0.999999266	
D24-D07	0.007463874	-0.036984639	0.051912387	0.999999442	
D00-B24	0.006693092	-0.037755421	0.051141605	0.999999876	
D03-B24	-0.006570711	-0.051019224	0.037877802	0.999999904	
D07-B14	-0.00604775	-0.050496263	0.038400763	0.99999997	
D19-D15	0.005769035	-0.038679478	0.050217549	0.999999985	
D14-B19	0.005732248	-0.038716265	0.050180761	0.999999986	
B24-B07	0.005164291	-0.04284559	0.053174173	0.999999999	
D19-B15	-0.004442202	-0.048890715	0.040006311	1	
D03-D01	-0.003730511	-0.048179025	0.040718002	1	
D19-D14	0.00317257	-0.041275943	0.047621083	1	
D15-B19	0.003135782	-0.041312731	0.047584295	1	
D01-B07	0.002324092	-0.04568579	0.050333973	1	
D03-B07	-0.00140642	-0.049416301	0.046603461	1	
D24-B14	0.001416124	-0.043032389	0.045864638	1	
D01-B24	-0.0028402	-0.047288713	0.041608313	1	
D15-D14	-0.002596466	-0.047044979	0.041852047	1	
	diff	lwr	upr	p adj	

tableS36 brain_cry1ab

	diff	lwr	upr	p adj	
B14-B03	-0.294671897	-0.433350373	-0.155993421	1.19E-07	**
B19-B03	-0.307982036	-0.457771909	-0.158192163	2.72E-07	**
B15-B03	-0.284000775	-0.422679251	-0.1453223	3.00E-07	**
D15-B03	-0.272837265	-0.411515741	-0.134158789	7.94E-07	**
D19-B03	-0.29179135	-0.441581224	-0.142001477	1.01E-06	**
D24-B03	-0.253331612	-0.392010087	-0.114653136	4.38E-06	**
B14-B01	-0.250718137	-0.389396612	-0.112039661	5.50E-06	**
D14-B03	-0.248221792	-0.386900267	-0.109543316	6.84E-06	**
B19-B01	-0.264028275	-0.413818149	-0.114238402	9.52E-06	**
B15-B01	-0.240047015	-0.37872549	-0.101368539	1.40E-05	**
B07-B03	-0.23878043	-0.377458906	-0.100101955	1.56E-05	**
D19-B01	-0.24783759	-0.397627463	-0.098047716	3.52E-05	**
D15-B01	-0.228883505	-0.36756198	-0.090205029	3.70E-05	**
D07-B03	-0.216419258	-0.355097733	-0.077740782	0.000108577	**
D24-B01	-0.209377851	-0.348056327	-0.070699375	0.000198476	**
D14-B01	-0.204268031	-0.342946507	-0.065589555	0.000306492	**
D00-B14	0.196659227	0.057980751	0.335337702	0.00058185	**
B07-B01	-0.19482667	-0.333505145	-0.056148194	0.000678159	**
D03-B14	0.210184554	0.060394681	0.359974428	0.000691503	**
D00-B19	0.209969366	0.060179492	0.359759239	0.000703087	**
D03-B19	0.223494693	0.063362583	0.383626804	0.000754162	**
D00-B15	0.185988105	0.047309629	0.324666581	0.00140865	**
D03-B15	0.199513433	0.049723559	0.349303306	0.001563435	**
D19-D03	-0.207304008	-0.367436118	-0.047171897	0.00238083	**
D19-D00	-0.19377868	-0.343568553	-0.043988807	0.002405191	**
B24-B03	-0.177864455	-0.31654293	-0.039185979	0.002722131	**
D15-D00	-0.174824595	-0.31350307	-0.036146119	0.003470138	**
D15-D03	-0.188349922	-0.338139796	-0.038560049	0.003595432	**
D07-B01	-0.172465497	-0.311143973	-0.033787021	0.004183135	**
D01-B14	0.168155759	0.029477283	0.306834235	0.005863298	**
D01-B19	0.181465898	0.031676025	0.331255771	0.005932524	**
D01-B15	0.157484637	0.018806162	0.296163113	0.013215425	*
D24-D03	-0.168844269	-0.318634142	-0.019054396	0.014409388	*
D24-D00	-0.155318941	-0.293997417	-0.016640466	0.01551432	*
D19-D01	-0.165275212	-0.315065086	-0.015485339	0.01836622	*
D14-D03	-0.163734449	-0.313524322	-0.013944576	0.020368735	*
D14-D00	-0.150209121	-0.288887597	-0.011530646	0.022495915	*
D15-D01	-0.146321127	-0.284999603	-0.007642651	0.029640811	*
B03-B00	0.154987087	0.005197213	0.30477696	0.036087319	*
D03-B07	0.154293088	0.004503214	0.304082961	0.037716913	*
D00-B07	0.14076776	0.002089284	0.279446236	0.043457257	*
B24-B01	-0.133910694	-0.27258917	0.004767782	0.068298202	
B19-B00	-0.152994949	-0.31312706	0.007137161	0.074740555	
B14-B00	-0.13968481	-0.289474684	0.010105063	0.091145883	
D24-D01	-0.126815474	-0.265493949	0.011863002	0.10617416	
D01-B03	-0.126516138	-0.265194614	0.012162338	0.108099035	
D07-D03	-0.131931915	-0.281721788	0.017857958	0.139634706	
D14-D01	-0.121705654	-0.260384129	0.016972822	0.143178679	
B24-B19	0.130117581	-0.019672292	0.279907455	0.153563656	
B15-B00	-0.129013689	-0.278803562	0.020776185	0.162558682	
D19-B00	-0.136804264	-0.296936374	0.023327847	0.171445679	
D07-D00	-0.118406587	-0.257085063	0.020271888	0.172097043	
B24-B14	0.116807443	-0.021871033	0.255485918	0.18763942	
D01-B07	0.112264292	-0.026414183	0.250942768	0.237464818	
D15-B00	-0.117850178	-0.267640052	0.031939695	0.277095724	
B24-B15	0.106136321	-0.032542155	0.244814796	0.31810369	
D19-B24	-0.113926896	-0.263716769	0.035862978	0.327687323	
B01-B00	0.111033326	-0.038756547	0.260823199	0.368205416	
D00-B03	-0.09801267	-0.236691146	0.040665805	0.446368016	
D15-B24	-0.094972811	-0.233651286	0.043705665	0.498996955	
D24-B00	-0.098344525	-0.248134398	0.051445348	0.568804671	
D07-D01	-0.089903119	-0.228581595	0.048775356	0.589332458	
D03-B24	0.093377112	-0.056412761	0.243166985	0.650690341	
D14-B00	-0.093234705	-0.243024578	0.056555168	0.653004577	
D07-B19	0.091562778	-0.058227095	0.241352652	0.679928995	
D01-B01	-0.082562378	-0.221240853	0.056116098	0.717345742	
D00-B24	0.079851784	-0.058826691	0.21853026	0.761131123	
D07-B14	0.07825264	-0.060425836	0.216931115	0.785590592	
D03-B03	-0.084487343	-0.234277216	0.065302531	0.786076771	
B07-B00	-0.083793344	-0.233583217	0.06599653	0.795569342	
D24-B24	-0.075467157	-0.214145633	0.063211319	0.825315386	
D14-B24	-0.070357337	-0.209035813	0.068321139	0.88719726	
D19-D07	-0.075372093	-0.225161966	0.074417781	0.893213171	
D07-B15	0.067581518	-0.071096958	0.206259993	0.914326033	
B19-B07	-0.069201606	-0.218991479	0.080588268	0.942059635	
D03-B00	0.070499744	-0.089632366	0.230631854	0.960296632	
B24-B07	0.060915976	-0.0777625	0.199594451	0.961020467	
D07-B00	-0.061432171	-0.211222044	0.088357702	0.978157428	
D15-D07	-0.056418008	-0.195096483	0.082260468	0.979654752	
B14-B07	-0.055891467	-0.194569942	0.082787009	0.981280165	
D14-B19	0.059760244	-0.090029629	0.209550118	0.982908468	
D00-B01	-0.05405891	-0.192737385	0.084619566	0.98616612	
D00-B00	0.056974416	-0.092815457	0.20676429	0.988997399	
D01-B24	0.051348316	-0.087330159	0.190026792	0.99148941	
D24-B19	0.054650424	-0.095139449	0.204440298	0.992626552	
D19-B07	-0.05301092	-0.202800793	0.096778953	0.994547332	
D14-B14	0.046450106	-0.09222837	0.185128581	0.996897252	
B15-B07	-0.045220345	-0.183898821	0.093458131	0.997662186	
B03-B01	0.04395376	-0.094724715	0.182632236	0.998277307	
D24-B14	0.041340286	-0.09733819	0.180018761	0.999125107	
D19-D14	-0.043569559	-0.193359432	0.106220315	0.99933758	
D03-D01	0.042028795	-0.107761078	0.191818669	0.999562396	
D07-B24	-0.038554803	-0.177233279	0.100123673	0.999606863	
D03-B01	-0.040533582	-0.190323455	0.109256291	0.999713837	
D24-D07	-0.036912354	-0.17559083	0.101766122	0.999764773	
D14-B15	0.035778984	-0.102899492	0.174457459	0.999838209	
D24-D19	0.038459739	-0.111330135	0.188249612	0.999847379	
D15-B07	-0.034056835	-0.17273531	0.104621641	0.999911395	
D15-B19	0.035144771	-0.114645102	0.184934644	0.999949757	
D14-D07	-0.031802534	-0.17048101	0.106875942	0.999962317	
D24-B15	0.030669164	-0.108009312	0.169347639	0.999976242	
D01-D00	-0.028503468	-0.167181943	0.110175008	0.999990782	
D01-B00	0.028470949	-0.121318925	0.178260822	0.999996717	
D15-D14	-0.024615474	-0.163293949	0.114063002	0.99999869	
D07-B07	0.022361173	-0.116317303	0.161039648	0.999999647	
B19-B15	-0.023981261	-0.173771134	0.125808613	0.99999968	
D15-B14	0.021834632	-0.116843844	0.160513108	0.999999746	
B24-B00	-0.022877368	-0.172667241	0.126912505	0.999999833	
D24-D15	0.019505654	-0.119172822	0.158184129	0.999999947	
D19-D15	-0.018954085	-0.168743959	0.130835788	0.999999988	
D24-B07	-0.014551181	-0.153229657	0.124127294	0.999999999	
D19-B19	0.016190686	-0.143941425	0.176322796	1	
D03-D00	0.013525328	-0.136264546	0.163315201	1	
B19-B14	-0.013310139	-0.163100012	0.136479734	1	
D15-B15	0.01116351	-0.127514965	0.149841986	1	
B15-B14	0.010671122	-0.128007354	0.149349597	1	
D14-B07	-0.009441361	-0.148119837	0.129237114	1	
D19-B14	0.002880547	-0.146909327	0.15267042	1	
D19-B15	-0.007790575	-0.157580448	0.141999298	1	
D24-D14	-0.00510982	-0.143788296	0.133568656	1	

tableS37 eye_cry1ab

	diff	lwr	upr	p adj	
B19-B01	-0.064467066	-0.090472551	-0.038461581	1.12E-09	**
D19-B01	-0.064372758	-0.090378243	-0.038367273	1.17E-09	**
D15-B01	-0.052976279	-0.078981763	-0.026970794	2.58E-07	**
B15-B01	-0.050791926	-0.076797411	-0.024786441	7.38E-07	**
D07-B01	-0.04916534	-0.075170824	-0.023159855	1.61E-06	**
B19-B03	-0.048023482	-0.074028967	-0.022017997	2.80E-06	**
D19-B03	-0.047929175	-0.073934659	-0.02192369	2.92E-06	**
D14-B01	-0.045399772	-0.071405257	-0.019394287	9.85E-06	**
B19-B14	-0.045340827	-0.071346311	-0.019335342	1.01E-05	**
D19-B14	-0.045246519	-0.071252004	-0.019241034	1.06E-05	**
D24-B01	-0.042459369	-0.068464854	-0.016453884	4.01E-05	**
B24-B19	0.042116897	0.016111412	0.068122382	4.71E-05	**
D19-B24	-0.04202259	-0.068028074	-0.016017105	4.93E-05	**
B07-B01	-0.044600669	-0.072689803	-0.016511535	6.96E-05	**
D01-B19	0.040800472	0.014794987	0.066805957	8.78E-05	**
D19-D01	-0.040706164	-0.066711649	-0.014700679	9.17E-05	**
D15-B03	-0.036532695	-0.06253818	-0.01052721	0.000633457	**
D03-B19	0.036416231	0.010410746	0.062421716	0.000667856	**
D19-D03	-0.036321923	-0.062327408	-0.010316438	0.00069704	**
B15-B03	-0.034348342	-0.060353827	-0.008342857	0.001687258	**
D15-B14	-0.033850039	-0.059855524	-0.007844554	0.002101451	**
D00-B19	0.032883018	0.006877533	0.058888502	0.003202209	**
D19-D00	-0.03278871	-0.058794195	-0.006783225	0.003335294	**
D07-B03	-0.032721756	-0.058727241	-0.006716271	0.003432984	**
B19-B00	-0.032515708	-0.058521193	-0.006510224	0.003751141	**
D19-B00	-0.032421401	-0.058426886	-0.006415916	0.003906014	**
B01-B00	0.031951357	0.005945872	0.057956842	0.004773703	**
B15-B14	-0.031665687	-0.057671172	-0.005660202	0.005387959	**
D00-B01	-0.031584048	-0.057589533	-0.005578563	0.005576904	**
D15-B24	-0.03062611	-0.056631595	-0.004620625	0.008320527	**
D07-B14	-0.0300391	-0.056044585	-0.004033615	0.010587962	*
D15-D01	-0.029309685	-0.055315169	-0.0033042	0.01421647	*
D14-B03	-0.028956188	-0.054961673	-0.002950703	0.016365955	*
B24-B15	0.028441757	0.002436272	0.054447242	0.020038134	*
D03-B01	-0.028050835	-0.05405632	-0.00204535	0.023322751	*
D01-B15	0.027125332	0.001119847	0.053130817	0.033160056	*
D07-B24	-0.026815171	-0.052820656	-0.000809686	0.037218836	*
D14-B14	-0.026273533	-0.052279017	-0.000268048	0.045391186	*
B07-B03	-0.028157085	-0.056246219	-6.80E-05	0.048882507	*
D24-B03	-0.026015785	-0.05202127	-1.03E-05	0.049815499	*
D07-D01	-0.025498746	-0.05150423	0.000506739	0.059856955	
D15-D03	-0.024925444	-0.050930928	0.001080041	0.07302377	
D01-B01	-0.023666594	-0.049672079	0.002338891	0.110874132	
B14-B07	0.02547443	-0.002614704	0.053563564	0.113817301	
D24-B14	-0.02333313	-0.049338614	0.002672355	0.123267953	
D14-B24	-0.023049603	-0.049055088	0.002955882	0.134671126	
D03-B15	0.022741091	-0.003264394	0.048746576	0.148023314	
B24-B01	-0.022350169	-0.048355654	0.003655316	0.166414345	
D24-B19	0.022007697	-0.003997788	0.048013182	0.183928494	
D24-D19	0.02191339	-0.004092095	0.047918874	0.188986989	
D14-D01	-0.021733178	-0.047738663	0.004272307	0.198940246	
D15-D00	-0.02139223	-0.047397715	0.004613255	0.21881298	
D07-D03	-0.021114505	-0.047119989	0.00489098	0.236017453	
D15-B00	-0.021024921	-0.047030406	0.004980564	0.24176235	
B24-B07	0.0222505	-0.005838634	0.050339634	0.270309638	
D24-B24	-0.0201092	-0.046114685	0.005886285	0.305899017	
D01-B07	0.020934075	-0.007155059	0.049023209	0.363598787	
D00-B15	0.019207878	-0.006797607	0.045213363	0.378133267	
B14-B01	-0.019126239	-0.045131724	0.006879246	0.385078131	
D14-B19	0.019067294	-0.006938191	0.045072779	0.390130441	
D19-D14	-0.018972987	-0.044978471	0.007032498	0.398278187	
B15-B00	-0.018840569	-0.044846054	0.007164916	0.409847959	
D24-D01	-0.018792775	-0.04479826	0.00721271	0.414059739	
B19-B07	-0.019866397	-0.047955531	0.008222737	0.449999769	
D19-B07	-0.019772089	-0.047861223	0.008317045	0.457991701	
D07-D00	-0.017581291	-0.043586776	0.008424194	0.525810221	
D14-D03	-0.017348937	-0.043354422	0.008656548	0.547968533	
D07-B00	-0.017213982	-0.043219467	0.008791503	0.560880099	
B03-B01	-0.016443584	-0.042449069	0.009561901	0.634426764	
B03-B00	0.015507774	-0.010497711	0.041513258	0.720499228	
D03-B07	0.016549834	-0.0115393	0.044638968	0.736755687	
D07-B19	0.015301726	-0.010703759	0.041307211	0.738525114	
D19-D07	-0.015207419	-0.041212904	0.010798066	0.746630945	
D00-B03	-0.015140465	-0.041145949	0.01086502	0.752327087	
D24-D03	-0.014408534	-0.040414019	0.01159695	0.810990499	
D14-D00	-0.013815724	-0.039821208	0.012189761	0.85289127	
B19-B15	-0.01367514	-0.039680625	0.012330345	0.862006289	
D19-B15	-0.013580832	-0.039586317	0.012424653	0.867937055	
D14-B00	-0.013448414	-0.039453899	0.01255707	0.876012125	
B14-B00	0.012825118	-0.013180367	0.038830603	0.909993621	
D00-B14	-0.012457809	-0.038463294	0.013547676	0.926895712	
D00-B07	0.013016621	-0.015072513	0.041105755	0.942975576	
B07-B00	-0.012649312	-0.040738446	0.015439822	0.954385467	
D03-B03	-0.011607251	-0.037612736	0.014398234	0.957514962	
D15-B19	0.011490787	-0.014514698	0.037496272	0.960840431	
D19-D15	-0.01139648	-0.037401965	0.014609005	0.963391102	
D24-D00	-0.010875321	-0.036880805	0.015130164	0.975338281	
D24-D15	0.01051691	-0.015488575	0.036522395	0.981648077	
D24-B00	-0.010508011	-0.036513496	0.015497473	0.981786987	
B24-B00	0.009601189	-0.016404296	0.035606673	0.992187337	
D00-B24	-0.00923388	-0.035239364	0.016771605	0.994694737	
D03-B14	-0.008924596	-0.034930081	0.017080889	0.996253517	
D24-B15	0.008332557	-0.017672928	0.034338042	0.998189529	
D01-B00	0.008284763	-0.017720722	0.034290248	0.998299169	
D01-D00	0.007917454	-0.018088031	0.033922939	0.998968719	
D15-B07	-0.00837561	-0.036464744	0.019713524	0.999184065	
D15-D14	-0.007576507	-0.033581992	0.018428978	0.999373476	
D01-B03	-0.00722301	-0.033228495	0.018782475	0.999640075	
D24-D07	0.006705971	-0.019299514	0.032711456	0.999851755	
B24-B03	-0.005906585	-0.03191207	0.0200989	0.999969435	
B15-B07	-0.006191257	-0.034280391	0.021897877	0.999979172	
D03-B24	-0.005700666	-0.031706151	0.020304819	0.999980589	
D14-B15	0.005392154	-0.020613331	0.031397639	0.999990567	
D01-B14	-0.004540355	-0.03054584	0.02146513	0.99999905	
D03-D01	-0.004384241	-0.030389726	0.021621244	0.999999411	
D07-B07	-0.004564671	-0.032653805	0.023524463	0.999999644	
D03-B00	0.003900522	-0.022104963	0.029906007	0.999999883	
D15-D07	-0.003810939	-0.029816424	0.022194546	0.999999915	
D14-D07	0.003765568	-0.022239917	0.029771053	0.999999928	
D03-D00	0.003533213	-0.022472272	0.029538698	0.999999971	
B24-B14	-0.00322393	-0.029229414	0.022781555	0.999999992	
D24-D14	0.002940403	-0.023065082	0.028945888	0.999999998	
B14-B03	-0.002682656	-0.02868814	0.023322829	0.999999999	
D15-B15	-0.002184353	-0.028189837	0.023821132	1	
D24-B07	0.0021413	-0.025947834	0.030230434	1	
D00-B00	0.000367309	-0.025638176	0.026372794	1	
D14-B07	-0.000799103	-0.028888237	0.027290031	1	
D07-B15	0.001626586	-0.024378898	0.027632071	1	
D19-B19	9.43E-05	-0.025911177	0.026099792	1	
D01-B24	-0.001316425	-0.02732191	0.02468906	1	

tableS38	brain_cry1ba				
	diff	lwr	upr	p adj	
B24-B15	-0.770544276	-1.221735843	-0.319352709	2.20E-05	**
B19-B15	-0.822392916	-1.304736991	-0.340048841	2.27E-05	**
D24-B15	-0.762567731	-1.244911805	-0.280223656	9.69E-05	**
D01-B15	-0.693906632	-1.145098199	-0.242715064	0.000159563	**
B15-B00	0.723631336	0.241287261	1.20597541	0.000246564	**
D00-B15	-0.673589311	-1.124780878	-0.222397743	0.000268248	**
B15-B01	0.660577148	0.178233074	1.142921223	0.001090134	**
D03-B15	-0.63133611	-1.113680184	-0.148992035	0.002137526	**
B19-B14	-0.574670275	-1.025861843	-0.123478708	0.003141823	**
D07-B15	-0.571764608	-1.022956176	-0.120573041	0.003369266	**
B24-B14	-0.522821635	-0.940543857	-0.105099413	0.003989263	**
D19-B15	-0.582217721	-1.064561796	-0.099873647	0.006416255	**
B19-B07	-0.523467027	-0.974658595	-0.07227546	0.010465028	*
D24-B14	-0.51484509	-0.966036657	-0.063653522	0.012729505	*
B24-B07	-0.471618387	-0.889340609	-0.053896166	0.014387328	*
D15-B19	0.507115689	0.055924121	0.958307256	0.015144349	*
D15-B24	0.455267049	0.037544827	0.872989271	0.021271248	*
D01-B14	-0.446183991	-0.863906213	-0.028461769	0.026309787	*
B14-B00	0.475908695	0.024717127	0.927100262	0.029926528	*
B19-B03	-0.464937512	-0.91612908	-0.013745945	0.037700654	*
D24-B07	-0.463641842	-0.914833409	-0.012450275	0.038730636	*
D00-B14	-0.42586667	-0.843588892	-0.008144448	0.041773169	*
D14-B19	0.457442182	0.006250614	0.908633749	0.044019524	*
D24-D15	-0.447290503	-0.898482071	0.003901064	0.054090226	
B24-B03	-0.413088873	-0.830811094	0.004633349	0.055299934	
D14-B24	0.405593542	-0.01212868	0.823315764	0.064930355	
D01-B07	-0.394980743	-0.812702965	0.022741479	0.081063457	
B07-B00	0.424705447	-0.02648612	0.875897014	0.084083065	
B14-B01	0.412854507	-0.03833706	0.864046075	0.104894947	
D15-D01	0.378629405	-0.039092817	0.796351626	0.112601206	
D15-B00	0.408354108	-0.042837459	0.859545676	0.113856796	
D24-B03	-0.405112327	-0.856303894	0.04607924	0.120697633	
D00-B07	-0.374663422	-0.792385644	0.0430588	0.121628968	
D24-D14	-0.397616996	-0.848808564	0.053574571	0.137808031	
D15-D00	0.358312084	-0.059410138	0.776034305	0.165257532	
D03-B14	-0.383613469	-0.834805036	0.067578099	0.174938574	
B03-B00	0.366175932	-0.085015635	0.817367499	0.231310747	
D14-B15	-0.364950734	-0.816142302	0.086240833	0.235711047	
D01-B03	-0.336451228	-0.75417345	0.081270994	0.241331491	
B07-B01	0.36165126	-0.089540308	0.812842827	0.247850837	
D14-B00	0.358680601	-0.092510966	0.809872169	0.259141836	
B15-B03	0.357455404	-0.093736164	0.808646971	0.263897959	
D14-D01	0.328955898	-0.088766324	0.746678119	0.272353514	
D07-B14	-0.324041967	-0.741764189	0.093680254	0.294055448	
D15-B01	0.345299921	-0.105891646	0.796491488	0.314177539	
D00-B03	-0.316133907	-0.733856129	0.101588315	0.33117951	
D19-B14	-0.33449508	-0.785686648	0.116696487	0.36337372	
D14-D00	0.308638577	-0.109083645	0.726360798	0.368733726	
D03-B07	-0.332410221	-0.783601788	0.118781346	0.373318424	
D15-D03	0.316058882	-0.135132685	0.76725045	0.455654441	
D15-B15	-0.315277227	-0.766468795	0.13591434	0.45975625	
B03-B01	0.303121745	-0.148069823	0.754313312	0.524903695	
B15-B07	0.298925889	-0.152265679	0.750117456	0.547808746	
D14-B01	0.295626414	-0.155565153	0.746817981	0.565892217	
D07-B07	-0.27283872	-0.690560942	0.144883502	0.57097804	
D19-B07	-0.283291833	-0.7344834	0.167899735	0.633376559	
D15-D07	0.256487381	-0.161234841	0.674209603	0.667049632	
D03-B03	-0.273880706	-0.725072273	0.177310861	0.68378915	
D19-D15	-0.266940494	-0.718132061	0.184251073	0.719753748	
D14-D03	0.266385375	-0.184806192	0.717576943	0.722572885	
D07-B19	0.250628308	-0.20056326	0.701819875	0.798005784	
B15-B14	0.247722641	-0.203468927	0.698914208	0.810784498	
D07-B03	-0.214309205	-0.632031427	0.203413017	0.874365728	
D19-B03	-0.224762318	-0.675953885	0.22642925	0.896452294	
D19-B19	0.240175195	-0.24216888	0.722519269	0.896758587	
D14-D07	0.206813874	-0.210908348	0.624536096	0.900664987	
D19-D14	-0.217266987	-0.668458554	0.23392458	0.918062364	
D07-B24	0.198779667	-0.218942554	0.61650189	0.924713803	
D24-D07	-0.190803122	-0.64199469	0.260388445	0.970018625	
D19-B24	0.188326555	-0.262865013	0.639518122	0.973150025	
D03-B19	0.191056806	-0.291287268	0.673400881	0.983019208	
D24-D19	-0.180350009	-0.662694084	0.301994065	0.990035109	
D07-B00	0.151866727	-0.29932484	0.603058295	0.996488343	
B19-B01	-0.161815768	-0.644159842	0.320528307	0.996606581	
D00-B19	0.148803605	-0.302387962	0.599995173	0.997159466	
D03-B24	0.139208166	-0.311983401	0.590399734	0.998610037	
D19-B00	0.141413614	-0.34093046	0.623757689	0.999212145	
D07-D01	0.122142024	-0.295580198	0.539864245	0.999235492	
D01-B19	0.128486284	-0.322705283	0.579677852	0.999434189	
D14-B14	-0.117228093	-0.534950315	0.300494128	0.999521843	
D24-D03	-0.131231621	-0.613575696	0.351112454	0.999666703	
B14-B03	0.109732763	-0.307989459	0.527454985	0.999779889	
D19-D01	0.111688911	-0.339502657	0.562880478	0.999892604	
D07-D00	0.101824703	-0.315897519	0.519546924	0.99991108	
B24-B01	-0.109967128	-0.561158695	0.34122444	0.99991124	
D00-B24	0.096954965	-0.320767257	0.514677187	0.999951618	
D24-B01	-0.101990582	-0.584334657	0.380353492	0.999985226	
B19-B00	-0.09876158	-0.581105655	0.383582494	0.999990268	
D19-D00	0.09137159	-0.359819978	0.542563157	0.99999157	
D24-D00	-0.08897842	-0.540169987	0.362213148	0.999994049	
D07-B01	0.08881254	-0.362379027	0.540004107	0.999994194	
D03-B00	0.092295226	-0.390048849	0.574639301	0.999996006	
D01-B24	0.076637644	-0.341084578	0.494359866	0.99999772	
D19-B01	0.078359427	-0.403984648	0.560703502	0.999999562	
D15-B14	-0.067554586	-0.485276808	0.350167635	0.999999589	
D14-B07	-0.066024846	-0.483747068	0.351697376	0.9999997	
D24-D01	-0.068661099	-0.519852666	0.382530469	0.999999822	
B07-B03	0.058529515	-0.359192707	0.476251737	0.999999944	
D03-D01	0.062570522	-0.388621045	0.51376209	0.999999951	
D07-D03	0.059571501	-0.391620066	0.510763069	0.999999976	
B01-B00	0.063054187	-0.419289887	0.545398262	0.999999979	
D24-B19	0.059825185	-0.422518889	0.54216926	0.99999999	
B14-B07	0.051203248	-0.366518974	0.46892547	0.999999992	
D15-D14	0.049673507	-0.368048715	0.467395729	0.999999995	
B24-B19	0.05184864	-0.399342927	0.503040207	0.999999997	
D00-B00	0.050042025	-0.401149543	0.501233592	0.999999998	
B24-B00	-0.04691294	-0.498104508	0.404278627	0.999999999	
D19-D03	0.049118388	-0.433225686	0.531462463	0.999999999	
D15-B03	0.042178176	-0.375544046	0.459900398	0.999999999	
D03-D00	0.042253201	-0.408938366	0.493444769	1	
D24-B00	-0.038936395	-0.52128047	0.44340768	1	
D01-B01	-0.033329483	-0.484521051	0.417862084	1	
D01-B00	0.029724704	-0.421466864	0.480916271	1	
D00-B01	-0.013012163	-0.46420373	0.438179405	1	
D03-B01	0.029241039	-0.453103036	0.511585113	1	
D14-B03	-0.007495331	-0.425217553	0.410226891	1	
D15-B07	-0.016351339	-0.434073561	0.401370883	1	
D24-B24	0.007976545	-0.443215022	0.459168113	1	
D01-D00	-0.020317321	-0.438039543	0.397404901	1	
D19-D07	-0.010453113	-0.46164468	0.440738454	1	

tableS39 eye_cry1ba

	diff	lwr	upr	p adj	
B14-B00	0.695549069	0.53659818	0.854499958	0	**
B15-B00	0.723845326	0.552158744	0.895531908	0	**
B14-B01	0.671706851	0.512755962	0.830657739	0	**
B15-B01	0.700003108	0.528316525	0.87168969	0	**
B14-B03	0.623013948	0.46406306	0.781964837	0	**
B15-B03	0.651310205	0.479623623	0.822996787	0	**
B19-B14	-0.621493414	-0.780444302	-0.462542525	0	**
B24-B14	-0.690094231	-0.84904512	-0.531143343	0	**
D00-B14	-0.690524417	-0.849475305	-0.531573528	0	**
D01-B14	-0.679441419	-0.838392307	-0.52049053	0	**
D03-B14	-0.657875869	-0.816826757	-0.49892498	0	**
D07-B14	-0.591512384	-0.750463272	-0.432561495	0	**
D19-B14	-0.63162459	-0.790575479	-0.472673701	0	**
D24-B14	-0.688506665	-0.847457553	-0.529555776	0	**
B19-B15	-0.64978967	-0.821476253	-0.478103088	0	**
B24-B15	-0.718390488	-0.89007707	-0.546703906	0	**
D00-B15	-0.718820673	-0.890507256	-0.547134091	0	**
D01-B15	-0.707737675	-0.879424258	-0.536051093	0	**
D03-B15	-0.686172126	-0.857858708	-0.514485543	0	**
D07-B15	-0.619808641	-0.791495223	-0.448122058	0	**
D19-B15	-0.659920847	-0.831607429	-0.488234265	0	**
D24-B15	-0.716802922	-0.888489504	-0.545116339	0	**
B14-B07	0.506253984	0.334567402	0.677940566	5.14E-12	**
B15-B07	0.534550241	0.351009564	0.718090918	8.02E-12	**
D15-B14	-0.414077621	-0.573028509	-0.255126732	2.94E-10	**
D15-B15	-0.442373877	-0.61406046	-0.270687295	4.11E-10	**
D14-B14	-0.354878427	-0.513829316	-0.195927539	2.56E-08	**
D14-B15	-0.383174684	-0.554861266	-0.211488102	2.58E-08	**
D14-B00	0.340670642	0.181719753	0.49962153	7.64E-08	**
D14-D00	0.335645989	0.176695101	0.494596878	1.13E-07	**
D14-B24	0.335215804	0.176264915	0.494166693	1.17E-07	**
D24-D14	-0.333628238	-0.492579126	-0.174677349	1.32E-07	**
D14-D01	0.324562991	0.165612103	0.48351388	2.66E-07	**
D14-B01	0.316828424	0.157877535	0.475779312	4.86E-07	**
D14-D03	0.302997442	0.144046553	0.46194833	1.43E-06	**
D15-B00	0.281471449	0.12252056	0.440422337	7.64E-06	**
D19-D14	-0.276746163	-0.435697051	-0.117795274	1.10E-05	**
D15-D00	0.276446796	0.117495907	0.435397685	1.13E-05	**
D15-B24	0.276016611	0.117065722	0.434967499	1.17E-05	**
D24-D15	-0.274429044	-0.433379933	-0.115478156	1.32E-05	**
D14-B03	0.268135521	0.109184632	0.42708641	2.15E-05	**
D14-B19	0.266614986	0.107664098	0.425565875	2.42E-05	**
D15-D01	0.265363798	0.106412909	0.424314687	2.67E-05	**
D15-B01	0.25762923	0.098678342	0.416580119	4.84E-05	**
D15-D03	0.243798248	0.08484736	0.402749137	0.000139259	**
D14-D07	0.236633957	0.077683068	0.395584845	0.000239423	**
D19-D15	-0.21754697	-0.376497858	-0.058596081	0.000988014	**
D15-B03	0.208936328	0.049985439	0.367887216	0.001843198	**
D15-B19	0.207415793	0.048464904	0.366366682	0.002055217	**
D15-D07	0.177434763	0.018483875	0.336385652	0.015965321	*
B07-B00	0.189295085	0.017608503	0.360981667	0.018372974	*
D00-B07	-0.184270432	-0.355957015	-0.01258385	0.024670269	*
B24-B07	-0.183840247	-0.355526829	-0.012153665	0.025292691	*
D24-B07	-0.182252681	-0.353939263	-0.010566098	0.027716346	*
D01-B07	-0.173187434	-0.344874017	-0.001500852	0.046078149	*
B07-B01	0.165452867	-0.006233716	0.337139449	0.069626747	
D03-B07	-0.151621885	-0.323308467	0.020064698	0.137816605	
D14-B07	0.151375557	-0.020311025	0.323062139	0.13940356	
D19-B07	-0.125370606	-0.297057188	0.046315976	0.395409356	
B07-B03	0.116759964	-0.054926618	0.288446546	0.514216175	
B19-B07	-0.115239429	-0.286926012	0.056447153	0.536087257	
D07-B00	0.104036685	-0.054914203	0.262987574	0.577588929	
D07-D00	0.099012033	-0.059938856	0.257962921	0.65565012	
D07-B24	0.098581848	-0.060369041	0.257532736	0.662220885	
D24-D07	-0.096994281	-0.25594517	0.061956608	0.686217614	
D07-D01	0.087929035	-0.071021854	0.246879923	0.811201432	
D15-B07	0.092176364	-0.079510219	0.263862946	0.841693048	
D07-B01	0.080194467	-0.078756422	0.239145356	0.893639791	
D07-B07	-0.0852584	-0.256944982	0.086428183	0.90453191	
B19-B00	0.074055656	-0.084895233	0.233006544	0.939846803	
B03-B00	0.072535121	-0.086415768	0.231486009	0.948704672	
D00-B19	-0.069031003	-0.227981892	0.089919886	0.965534985	
B24-B19	-0.068600818	-0.227551706	0.090350071	0.967278711	
D00-B03	-0.067510468	-0.226461357	0.09144042	0.971404803	
B24-B03	-0.067080283	-0.226031172	0.091870606	0.972920555	
D24-B19	-0.067013251	-0.22596414	0.091937637	0.973151181	
D07-D03	0.066363485	-0.092587404	0.225314374	0.97531065	
D24-B03	-0.065492717	-0.224443605	0.093458172	0.977994682	
D19-B00	0.063924479	-0.09502641	0.222875368	0.982258527	
D15-D14	-0.059199193	-0.218150082	0.099751695	0.991338118	
D19-D00	0.058899826	-0.100051062	0.217850715	0.991753252	
D19-B24	0.058469641	-0.100481247	0.21742053	0.992321471	
D01-B19	-0.057948005	-0.216898894	0.101002884	0.992967439	
D24-D19	-0.056882075	-0.215832963	0.102068814	0.994150149	
D01-B03	-0.05642747	-0.215378359	0.102523418	0.994602273	
B19-B01	0.050213437	-0.108737451	0.209164326	0.99841386	
B03-B01	0.048692903	-0.110257986	0.207643791	0.998869719	
D19-D01	0.047816829	-0.11113406	0.206767717	0.999077364	
D19-D07	-0.040112206	-0.199063095	0.118838682	0.999883042	
D19-B01	0.040082261	-0.118868628	0.199033149	0.999884106	
D03-B00	0.0376732	-0.121277688	0.196624089	0.999946202	
D03-B19	-0.036382455	-0.195333344	0.122568433	0.999965327	
D03-B03	-0.034861921	-0.193812809	0.124088968	0.999979893	
D03-D00	0.032648548	-0.126302341	0.191599436	0.999991414	
D03-B24	0.032218362	-0.126732526	0.191169251	0.999992785	
D07-B03	0.031501565	-0.127449324	0.190452453	0.999994637	
D24-D03	-0.030630796	-0.189581685	0.128320093	0.999996302	
D07-B19	0.02998103	-0.128969859	0.188931918	0.999997222	
D19-D03	0.026251279	-0.13269961	0.185202167	0.999999541	
B15-B14	0.028296257	-0.143390325	0.199982839	0.999999554	
B01-B00	0.023842218	-0.13510867	0.182793107	0.999999879	
D03-D01	0.02156555	-0.137385339	0.180516438	0.99999997	
D00-B01	-0.018817566	-0.177768454	0.140133323	0.999999996	
B24-B01	-0.01838738	-0.177338269	0.140563508	0.999999997	
D24-B01	-0.016799814	-0.175750703	0.142151075	0.999999999	
D01-B00	0.01610765	-0.142843238	0.175058539	1	
D03-B01	0.013830982	-0.145119907	0.172781871	1	
D01-D00	0.011082998	-0.147867891	0.170033887	1	
B24-B00	0.005454838	-0.153496051	0.164405726	1	
D00-B00	0.005024653	-0.153926236	0.163975541	1	
D24-B00	0.007042404	-0.151908484	0.165993293	1	
D01-B01	-0.007734568	-0.166685456	0.151216321	1	
B19-B03	0.001520535	-0.157430354	0.160471423	1	
D19-B03	-0.008610642	-0.16756153	0.150340247	1	
D19-B19	-0.010131177	-0.169082065	0.148819712	1	
D00-B24	-0.000430185	-0.159381074	0.158520703	1	
D01-B24	0.010652813	-0.148298076	0.169603701	1	
D24-B24	0.001587566	-0.157363322	0.160538455	1	
D24-D00	0.002017752	-0.156933137	0.16096864	1	
D24-D01	-0.009065246	-0.168016135	0.149885642	1	

tableS40	brain_cry1bb				
	diff	lwr	upr	p adj	
D24-B15	-0.367314163	-0.492165189	-0.242463138	1.39E-11	**
D01-B15	-0.361380309	-0.486231335	-0.236529284	2.24E-11	**
D07-B15	-0.358714632	-0.483565658	-0.233863607	2.78E-11	**
D00-B15	-0.355592035	-0.480443061	-0.23074101	3.61E-11	**
B15-B01	0.339045372	0.214194346	0.463896397	1.50E-10	**
D03-B15	-0.361782386	-0.496636907	-0.226927866	2.16E-10	**
B24-B15	-0.354435213	-0.489289733	-0.219580692	3.96E-10	**
B15-B00	0.346737429	0.211882908	0.481591949	7.51E-10	**
B19-B15	-0.33655793	-0.471412451	-0.20170341	1.77E-09	**
D19-B15	-0.323494889	-0.45834941	-0.188640369	5.37E-09	**
D24-B14	-0.298167753	-0.423018779	-0.173316727	6.07E-09	**
D01-B14	-0.292233899	-0.417084924	-0.167382873	1.05E-08	**
D07-B14	-0.289568222	-0.414419247	-0.164717196	1.35E-08	**
B15-B03	0.28677703	0.161926004	0.411628056	1.75E-08	**
D00-B14	-0.286445625	-0.411296651	-0.161594599	1.81E-08	**
D14-B15	-0.272522513	-0.397373539	-0.147671488	6.70E-08	**
D03-B14	-0.292635976	-0.427490497	-0.157781455	7.79E-08	**
B14-B01	0.269898961	0.145047936	0.394749987	8.58E-08	**
D15-B15	-0.266081253	-0.390932278	-0.141230227	1.23E-07	**
B24-B14	-0.285288802	-0.420143323	-0.150434282	1.48E-07	**
B14-B00	0.277591018	0.142736498	0.412445539	2.93E-07	**
B19-B14	-0.26741152	-0.402266041	-0.132556999	7.21E-07	**
D19-B14	-0.254348479	-0.389202999	-0.119493958	2.30E-06	**
B14-B03	0.21763062	0.092779594	0.342481645	1.28E-05	**
B15-B07	0.207533109	0.082682083	0.332384135	3.37E-05	**
D14-B14	-0.203376103	-0.328227129	-0.078525077	5.02E-05	**
D15-B14	-0.196934842	-0.321785868	-0.072083817	9.26E-05	**
D24-B07	-0.159781055	-0.28463208	-0.034930029	0.002849752	**
D01-B07	-0.1538472	-0.278698226	-0.028996175	0.004791749	**
D07-B07	-0.151181523	-0.276032549	-0.026330498	0.006030271	**
D00-B07	-0.148058927	-0.272909952	-0.023207901	0.007869755	**
D03-B07	-0.154249278	-0.289103798	-0.019394757	0.0122152	*
B14-B07	0.138386699	0.013535673	0.263237724	0.017532263	*
B24-B07	-0.146902104	-0.281756624	-0.012047583	0.021224513	*
B07-B01	0.131512263	0.006661237	0.256363288	0.03021136	*
B07-B00	0.13920432	0.004349799	0.27405884	0.036980543	*
B19-B07	-0.129024822	-0.263879342	0.005829699	0.073766503	
D19-B07	-0.11596178	-0.250816301	0.01889274	0.163762012	
D24-D15	-0.101232911	-0.226083936	0.023618115	0.234423887	
D15-D01	0.095299057	-0.029551969	0.220150082	0.320889615	
D24-D14	-0.09479165	-0.219642676	0.030059376	0.329083121	
D15-D07	0.09263338	-0.032217646	0.217484405	0.365239494	
D15-D00	0.089510783	-0.035340243	0.214361808	0.42095929	
D14-D01	0.088857796	-0.03599323	0.213708821	0.433058099	
D15-D03	0.095701134	-0.039153387	0.230555654	0.437834346	
D14-D07	0.086192119	-0.038658907	0.211043144	0.483746074	
D14-D00	0.083069522	-0.041781504	0.207920548	0.545013012	
D14-D03	0.089259873	-0.045594648	0.224114394	0.553559087	
D15-B24	0.08835396	-0.04650056	0.223208481	0.570217841	
D24-B03	-0.080537134	-0.205388159	0.044313892	0.595306879	
B07-B03	0.079243921	-0.045607105	0.204094947	0.620914621	
D14-B24	0.081912699	-0.052941821	0.21676722	0.687118376	
D15-B00	0.080656176	-0.054198344	0.215510697	0.709018963	
D01-B03	-0.074603279	-0.199454305	0.050247746	0.710318681	
D15-B01	0.072964119	-0.051886907	0.197815145	0.740274293	
D07-B03	-0.071937602	-0.196788628	0.052913423	0.758431339	
D03-B03	-0.075005357	-0.209859877	0.059849164	0.800296858	
B15-B14	0.06914641	-0.055704615	0.193997436	0.805009376	
D00-B03	-0.068815006	-0.193666031	0.05603602	0.810238744	
D14-B00	0.074214915	-0.060639605	0.209069436	0.811886706	
D14-B01	0.066522858	-0.058328167	0.191373884	0.844466044	
D15-B19	0.070476678	-0.064377843	0.205331198	0.861941979	
D14-B07	-0.064989404	-0.18984043	0.059861621	0.865357295	
B24-B03	-0.067658183	-0.202512703	0.067196338	0.894066099	
D14-B19	0.064035417	-0.070819104	0.198889938	0.927936344	
D15-B07	-0.058548144	-0.183399169	0.066302882	0.934271268	
B03-B00	0.059960399	-0.074894122	0.194814919	0.956353362	
D19-D15	-0.057413636	-0.192268157	0.077440884	0.969384229	
B03-B01	0.052268342	-0.072582684	0.177119367	0.973444397	
D19-D14	-0.050972376	-0.185826896	0.083882145	0.989421911	
B19-B03	-0.049780901	-0.184635421	0.08507362	0.991562704	
D24-D19	-0.043819274	-0.178673795	0.091035246	0.997692998	
D19-D01	0.03788542	-0.0969691	0.172739941	0.999543579	
D19-B03	-0.036717859	-0.17157238	0.098136661	0.999683382	
D19-D03	0.038287497	-0.105878049	0.182453044	0.999764248	
D19-D07	0.035219743	-0.099634777	0.170074264	0.99980703	
D19-D00	0.032097146	-0.102757374	0.166951667	0.999938014	
D24-B19	-0.030756233	-0.165610754	0.104098288	0.999963717	
D24-B01	-0.028268792	-0.153119817	0.096582234	0.999966902	
D19-B24	0.030940324	-0.113225223	0.17510587	0.999983306	
D01-B19	-0.024822379	-0.159676899	0.110032142	0.999997788	
D01-B01	-0.022334937	-0.147185963	0.102516088	0.999998493	
D03-B19	-0.025224456	-0.169390003	0.118941091	0.999998883	
D03-B01	-0.022737015	-0.157591535	0.112117506	0.999999325	
D15-B03	0.020695777	-0.104155248	0.145546803	0.999999465	
D07-B19	-0.022156702	-0.157011222	0.112697819	0.999999526	
D19-B00	0.02324254	-0.120923007	0.167408086	0.999999635	
D07-B01	-0.01966926	-0.144520286	0.105181765	0.999999734	
D24-B00	-0.020576735	-0.155431255	0.114277786	0.999999829	
D00-B19	-0.019034105	-0.153888626	0.115820416	0.999999943	
D00-B01	-0.016546664	-0.141397689	0.108304362	0.999999976	
B24-B19	-0.017877282	-0.162042829	0.126288264	0.999999991	
D19-B01	0.015550483	-0.119304038	0.150405003	0.999999997	
D14-B03	0.014254517	-0.110596509	0.139105542	0.999999997	
B24-B01	-0.015389841	-0.150244362	0.119464679	0.999999997	
D01-B00	-0.01464288	-0.149497401	0.12021164	0.999999999	
D03-B00	-0.015044958	-0.159210504	0.129120589	0.999999999	
D24-B24	-0.012878951	-0.147733471	0.12197557	1	
D24-D00	-0.011722128	-0.136573154	0.113128898	1	
D19-B19	0.013063041	-0.131102505	0.157228588	1	
D07-B00	-0.011977203	-0.146831724	0.122877317	1	
B19-B00	0.010179498	-0.133986048	0.154345045	1	
D24-D07	-0.008599531	-0.133450557	0.116251494	1	
B01-B00	0.007692057	-0.127162463	0.142546578	1	
B24-B00	-0.007697784	-0.151863331	0.136467763	1	
D00-B00	-0.008854607	-0.143709127	0.125999914	1	
B19-B01	0.002487441	-0.132367079	0.137341962	1	
D00-B24	-0.001156823	-0.136011343	0.133697698	1	
D01-B24	-0.006945096	-0.141799617	0.127909424	1	
D03-B24	-0.007347174	-0.15151272	0.136818373	1	
D07-B24	-0.004279419	-0.13913394	0.130575101	1	
D01-D00	-0.005788274	-0.130639299	0.119062752	1	
D03-D00	-0.006190351	-0.141044872	0.12866417	1	
D07-D00	-0.003122597	-0.127973622	0.121728429	1	
D03-D01	-0.000402077	-0.135256598	0.134452443	1	
D07-D01	0.002665677	-0.122185349	0.127516703	1	
D24-D01	-0.005933854	-0.13078488	0.118917171	1	
D07-D03	0.003067754	-0.131786766	0.137922275	1	
D24-D03	-0.005531777	-0.140386298	0.129322744	1	
D15-D14	0.006441261	-0.118409765	0.131292286	1	

tableS41	eye_cry1bb				
	diff	lwr	upr	p adj	
B14-B00	0.160556251	0.127025746	0.194086756	2.40E-13	**
B14-B01	0.155967592	0.122437087	0.189498096	2.40E-13	**
B24-B14	-0.159150871	-0.192681376	-0.125620366	2.40E-13	**
D00-B14	-0.159739689	-0.193270194	-0.126209184	2.40E-13	**
D01-B14	-0.160054616	-0.193585121	-0.126524111	2.40E-13	**
D03-B14	-0.158925776	-0.192456281	-0.125395271	2.40E-13	**
D19-B14	-0.153149829	-0.186680334	-0.119619324	2.40E-13	**
D07-B14	-0.145982301	-0.179512806	-0.112451796	2.41E-13	**
D24-B14	-0.159550914	-0.195767999	-0.12333383	2.41E-13	**
B19-B14	-0.150684432	-0.186901516	-0.114467347	2.41E-13	**
B14-B03	0.134608972	0.101078467	0.168139477	2.43E-13	**
B15-B00	0.118673638	0.085143133	0.152204142	2.75E-13	**
D01-B15	-0.118172003	-0.151702507	-0.084641498	2.76E-13	**
D00-B15	-0.117857075	-0.15138758	-0.08432657	2.78E-13	**
B24-B15	-0.117268258	-0.150798762	-0.083737753	2.83E-13	**
D03-B15	-0.117043163	-0.150573668	-0.083512658	2.84E-13	**
D15-B14	-0.116380794	-0.149911299	-0.082850289	2.90E-13	**
B15-B01	0.114084978	0.080554473	0.147615483	3.27E-13	**
D14-B14	-0.112657835	-0.14618834	-0.07912733	3.66E-13	**
D19-B15	-0.111267216	-0.144797721	-0.077736711	4.19E-13	**
D24-B15	-0.117668301	-0.153885385	-0.081451216	5.75E-13	**
D07-B15	-0.104099688	-0.137630192	-0.070569183	1.64E-12	**
B19-B15	-0.108801818	-0.145018903	-0.072584734	4.28E-12	**
B15-B03	0.092726359	0.059195854	0.126256864	5.66E-11	**
B14-B07	0.099604597	0.063387513	0.135821682	6.72E-11	**
D15-B15	-0.07449818	-0.108028685	-0.040967675	3.29E-08	**
D14-B15	-0.070775222	-0.104305727	-0.037244717	1.26E-07	**
B07-B00	0.060951654	0.024734569	0.097168738	2.37E-05	**
D01-B07	-0.060450019	-0.096667103	-0.024232934	2.81E-05	**
D00-B07	-0.060135092	-0.096352176	-0.023918007	3.12E-05	**
B24-B07	-0.059546274	-0.095763358	-0.023329189	3.81E-05	**
D03-B07	-0.059321179	-0.095538264	-0.023104095	4.10E-05	**
B15-B07	0.057721984	0.021504899	0.093939068	7.00E-05	**
B07-B01	0.056362994	0.02014591	0.092580079	0.000110089	**
D24-B07	-0.059946317	-0.098664009	-0.021228625	0.000121136	**
D19-B07	-0.053545232	-0.089762317	-0.017328148	0.000278759	**
D14-B00	0.047898416	0.014367911	0.081428921	0.000502406	**
D14-D01	0.047396781	0.013866276	0.080927286	0.000598548	**
D14-D00	0.047081854	0.013551349	0.080612358	0.000667869	**
D14-B24	0.046493036	0.012962531	0.080023541	0.000819129	**
D14-D03	0.046267941	0.012737436	0.079798446	0.000885383	**
B19-B07	-0.051079835	-0.089797527	-0.012362143	0.001773548	**
D15-B00	0.044175457	0.010644952	0.077705962	0.001810527	**
D15-D01	0.043673822	0.010143317	0.077204327	0.002144408	**
D24-D14	-0.046893079	-0.083110163	-0.010675994	0.002339569	**
D15-D00	0.043358895	0.00982839	0.0768894	0.002383649	**
D14-B01	0.043309756	0.009779251	0.076840261	0.00242323	**
D07-B07	-0.046377704	-0.082594788	-0.010160619	0.002744456	**
D15-B24	0.042770077	0.009239572	0.076300582	0.002901837	**
D15-D03	0.042544983	0.009014478	0.076075487	0.003127306	**
B15-B14	-0.041882614	-0.075413118	-0.008352109	0.003892764	**
D19-D14	-0.040491994	-0.074022499	-0.006961489	0.006124818	**
D24-D15	-0.04317012	-0.079387205	-0.006953036	0.007248333	**
D15-B01	0.039586798	0.006056293	0.073117303	0.008184788	**
D19-D15	-0.036769036	-0.07029954	-0.003238531	0.019594014	*
D14-B19	0.038026597	0.001809512	0.074243681	0.031103611	*
D14-D07	0.033324466	-0.000206039	0.066854971	0.052921408	
B07-B03	0.035004375	-0.001212709	0.07122146	0.067823828	
D15-B19	0.034303638	-0.001913446	0.070520723	0.080464584	
D15-D07	0.029601507	-0.003928998	0.063132012	0.137779265	
B03-B00	0.025947279	-0.007583226	0.059477784	0.302580446	
D01-B03	-0.025445644	-0.058976149	0.008084861	0.332426005	
D00-B03	-0.025130717	-0.058661221	0.008399788	0.351991169	
B24-B03	-0.024541899	-0.058072404	0.008988606	0.390165569	
D03-B03	-0.024316804	-0.057847309	0.009213701	0.405266269	
D24-B03	-0.024941942	-0.061159026	0.011275143	0.491436772	
D14-B03	0.021951137	-0.011579368	0.055481642	0.575404871	
B03-B01	0.021358619	-0.012171886	0.054889124	0.619244694	
D19-B03	-0.018540857	-0.052071362	0.014989648	0.810087708	
D15-B03	0.018228179	-0.015302326	0.051758683	0.827922044	
B15-B07	-0.016776197	-0.052993281	0.019440888	0.941682478	
B19-B03	-0.01607546	-0.052292544	0.020141625	0.958226885	
D07-B00	0.01457395	-0.018956555	0.048104455	0.96478325	
D07-D01	0.014072315	-0.01945819	0.04760282	0.973767167	
D07-D00	0.013757388	-0.019773117	0.047287893	0.978446909	
D07-B24	0.01316857	-0.020361935	0.046699075	0.985452406	
D07-D03	0.012943475	-0.02058703	0.04647398	0.987598842	
D24-D07	-0.013568613	-0.049785698	0.022648471	0.990655737	
D14-B07	-0.013053238	-0.049270323	0.023163846	0.993594543	
D07-B03	-0.011373329	-0.044903834	0.022157176	0.996540279	
D07-B01	0.009985291	-0.023545214	0.043515795	0.999156666	
B19-B00	0.009871819	-0.026345265	0.046088904	0.999696577	
D01-B19	-0.009370184	-0.045587269	0.0268469	0.999837287	
D00-B19	-0.009055257	-0.045272341	0.027161828	0.999892679	
B24-B19	-0.008466439	-0.044683524	0.027750645	0.999953427	
D24-B19	-0.008866482	-0.047584174	0.02985121	0.999964083	
D03-B19	-0.008241344	-0.044458429	0.02797574	0.99996686	
D19-B00	0.007406422	-0.026124083	0.040936927	0.999977316	
D19-D07	-0.007167528	-0.040698033	0.026362977	0.999985125	
D19-D01	0.006904787	-0.026625718	0.040435292	0.999990847	
D19-D00	0.00658986	-0.026940645	0.040120364	0.999995047	
D19-B24	0.006001042	-0.027529463	0.039531547	0.999998584	
D24-D19	-0.006401085	-0.042618169	0.029816	0.999998805	
D19-D03	0.005775947	-0.027754558	0.039306452	0.999999157	
B19-B01	0.00528316	-0.030933925	0.041500244	0.999999915	
B01-B00	0.004588659	-0.028941845	0.038119164	0.999999965	
D07-B19	0.004702131	-0.031514954	0.040919215	0.999999984	
D01-B01	-0.004087024	-0.037617529	0.02944348	0.999999993	
D00-B01	-0.003772097	-0.037302602	0.029758408	0.999999998	
D15-D14	-0.003722959	-0.037253463	0.029807546	0.999999998	
D24-B01	-0.003583323	-0.039800407	0.032633762	1	
B24-B01	-0.003183279	-0.036713784	0.030347225	1	
D03-B01	-0.002958185	-0.03648869	0.03057232	1	
D19-B01	0.002817762	-0.030712743	0.036348267	1	
D19-B19	-0.002465397	-0.038682482	0.033751687	1	
B24-B00	0.00140538	-0.032125125	0.034935885	1	
D00-B00	0.000816562	-0.032713943	0.034347067	1	
D01-B00	0.000501635	-0.03302887	0.03403214	1	
D03-B00	0.001630475	-0.03190003	0.03516098	1	
D24-B00	0.001005337	-0.035211748	0.037222421	1	
D00-B24	-0.000588818	-0.034119323	0.032941687	1	
D01-B24	-0.000903745	-0.03443425	0.03262676	1	
D03-B24	0.000225095	-0.03330541	0.0337556	1	
D24-B24	-0.000400043	-0.036617128	0.035817041	1	
D01-D00	-0.000314927	-0.033845432	0.033215578	1	
D03-D00	0.000813912	-0.032716592	0.034344417	1	
D24-D00	0.000188775	-0.03602831	0.036405859	1	
D03-D01	0.00112884	-0.032401665	0.034659345	1	
D24-D01	0.000503702	-0.035713383	0.036720786	1	
D24-D03	-0.000625138	-0.036842222	0.035591947	1	

tableS42 brain_cry2

	diff	lwr	upr	p adj	
B14-B01	-0.563759007	-1.030465737	-0.097052277	0.006161606	**
B15-B01	-0.535502588	-1.002209318	-0.068795858	0.011694882	*
D15-B01	-0.524750484	-0.991457214	-0.058043754	0.014835074	*
B07-B01	-0.502054262	-0.968760992	-0.035347531	0.02421132	*
D14-B01	-0.500403256	-0.967109986	-0.033696526	0.025072213	*
B14-B00	-0.520616596	-1.024717479	-0.016515712	0.036759644	*
D00-B14	0.465210591	-0.001496139	0.931917321	0.051500442	
B14-B03	-0.463875256	-0.930581986	0.002831474	0.052873225	
D07-B01	-0.457614062	-0.924320792	0.009092668	0.059751024	
B15-B00	-0.492360177	-0.99646106	0.011740707	0.061841352	
B19-B01	-0.489510832	-0.993611716	0.014590051	0.065062389	
D15-B00	-0.481608073	-0.985708956	0.022492811	0.074772896	
D00-B15	0.436954173	-0.029752558	0.903660903	0.088294335	
B15-B03	-0.435618837	-0.902325567	0.031087893	0.090485738	
D19-B01	-0.461327141	-0.965428025	0.042773742	0.105563423	
D15-D00	-0.426202068	-0.892908798	0.040504662	0.107280944	
B07-B00	-0.45891185	-0.963012734	0.045189033	0.109854745	
D15-B03	-0.424866733	-0.891573463	0.041839997	0.109860758	
D14-B00	-0.457260845	-0.961361728	0.046840039	0.112870535	
B24-B14	0.414506206	-0.052200524	0.881212936	0.131663923	
D00-B07	0.403505846	-0.063200884	0.870212576	0.158501777	
B07-B03	-0.402170511	-0.868877241	0.064536219	0.162032576	
D14-D00	-0.401854841	-0.868561571	0.06485189	0.162876112	
D14-B03	-0.400519505	-0.867226235	0.066187225	0.166482111	
B19-B00	-0.446368421	-0.985274933	0.092538091	0.207900862	
B24-B15	0.386249788	-0.080456943	0.852956518	0.208921473	
D07-B00	-0.414471651	-0.918572534	0.089629232	0.217186824	
D24-B01	-0.380313108	-0.847019838	0.086393623	0.228734785	
D15-B24	-0.375497683	-0.842204413	0.091209047	0.245752112	
D01-B14	0.372207726	-0.094499005	0.838914456	0.257865998	
D19-B00	-0.41818473	-0.957091242	0.120721782	0.297291491	
D00-B19	0.390962417	-0.113138467	0.8950633	0.298103348	
B19-B03	-0.389627082	-0.893727965	0.114473802	0.303210216	
D07-D00	-0.359065647	-0.825772377	0.107641083	0.310146463	
D07-B03	-0.357730312	-0.824437042	0.108976419	0.315798515	
B24-B07	0.352801461	-0.113905269	0.819508191	0.337181605	
D14-B24	-0.351150456	-0.817857186	0.115556275	0.34452325	
D01-B15	0.343951307	-0.122755423	0.810658037	0.377534375	
D19-D00	-0.362778726	-0.866879609	0.141322158	0.416383355	
D19-B03	-0.361443391	-0.865544274	0.142657493	0.422475046	
D15-D01	-0.333199203	-0.799905933	0.133507528	0.429578728	
B24-B19	0.340258032	-0.163842852	0.844358915	0.523172802	
D24-B00	-0.337170696	-0.84127158	0.166930187	0.538289901	
D01-B07	0.31050298	-0.15620375	0.77720971	0.547076416	
D14-D01	-0.308851975	-0.775558705	0.157854755	0.555847952	
D07-B24	-0.308361262	-0.775067992	0.158345468	0.55845706	
D19-B24	-0.312074341	-0.816175224	0.192026543	0.661172334	
D24-D00	-0.281764692	-0.748471422	0.184942038	0.69782432	
D24-B03	-0.280429357	-0.747136087	0.186277373	0.70453492	
D03-B14	0.301681293	-0.20241959	0.805782176	0.710162251	
D01-B19	0.297959551	-0.206141332	0.802060434	0.7271565	
D07-D01	-0.266062781	-0.732769511	0.200643949	0.773402748	
D03-B15	0.273424874	-0.230676009	0.777525757	0.828656601	
D19-D01	-0.26977586	-0.773876743	0.234325023	0.841850562	
D15-D03	-0.26267277	-0.766773653	0.241428113	0.865906779	
D03-B01	-0.262077714	-0.766178597	0.242023169	0.86782171	
D24-B24	-0.231060307	-0.697767037	0.235646423	0.904359619	
D03-B07	0.239976548	-0.264124336	0.744077431	0.927566641	
D14-D03	-0.238325542	-0.742426425	0.265775341	0.931140408	
D03-B19	0.227433118	-0.311473394	0.766339631	0.97204018	
D01-B01	-0.191551281	-0.658258011	0.275155449	0.978012637	
D03-B00	-0.218935303	-0.757841815	0.31997121	0.979904396	
D24-D01	-0.188761826	-0.655468556	0.277944904	0.980682021	
D24-B14	0.183445899	-0.283260831	0.650152629	0.985069956	
D07-D03	-0.195536348	-0.699637232	0.308564535	0.986784356	
D19-D03	-0.199249427	-0.73815594	0.339657085	0.991608593	
D24-B15	0.155189481	-0.31151725	0.621896211	0.997124983	
D03-D00	-0.163529298	-0.667630182	0.340571585	0.997787681	
D03-B03	-0.162193963	-0.666294846	0.34190692	0.99797372	
B24-B01	-0.149252801	-0.615959531	0.31745393	0.998101996	
D24-D15	0.144437376	-0.322269354	0.611144106	0.998672347	
D01-B00	-0.14840887	-0.652509753	0.355692013	0.999240152	
D24-B07	0.121741154	-0.344965576	0.588447884	0.999815284	
D24-D14	0.120090149	-0.346616582	0.586796879	0.999843331	
D24-D03	-0.118235394	-0.622336277	0.38586549	0.999949971	
D07-B14	0.106144945	-0.360561786	0.572851675	0.999966067	
D03-B24	-0.112824913	-0.616925797	0.39127597	0.999972324	
D24-B19	0.109197725	-0.394903159	0.613298608	0.999981786	
B03-B01	-0.099883751	-0.566590481	0.366822979	0.999984413	
D00-B01	-0.098548416	-0.565255146	0.368158315	0.999986904	
B24-B00	-0.106110389	-0.610211273	0.397990494	0.999987427	
D19-B14	0.102431866	-0.401669018	0.606532749	0.999992064	
D01-D00	-0.093002866	-0.559709596	0.373703864	0.999993857	
D01-B03	-0.091667531	-0.558374261	0.3750392	0.999994925	
D07-B15	0.077888526	-0.388818204	0.544595256	0.999999434	
D24-D07	0.077300955	-0.389405775	0.544007685	0.99999949	
D24-D19	0.081014034	-0.42308685	0.585114917	0.999999663	
B19-B14	0.074248175	-0.429852709	0.578349058	0.999999899	
D19-B15	0.074175447	-0.429925437	0.57827633	0.999999901	
D15-D07	-0.067136422	-0.533843152	0.399570309	0.999999928	
D03-D01	-0.070526433	-0.574627316	0.433574451	0.999999951	
D14-B14	0.063355751	-0.403350979	0.530062481	0.999999968	
B14-B07	-0.061704745	-0.528411475	0.405001985	0.999999978	
D19-D15	0.063423343	-0.440677541	0.567524226	0.999999989	
B03-B00	-0.056741339	-0.560842223	0.447359544	0.999999998	
D00-B00	-0.055406004	-0.559506888	0.448694879	0.999999998	
D00-B24	0.050704385	-0.416002345	0.517411115	0.999999999	
B24-B03	-0.04936905	-0.51607578	0.41733768	0.999999999	
D07-B07	0.044440199	-0.422266531	0.511146929	1	
D14-D07	-0.042789194	-0.509495924	0.423917536	1	
B19-B15	0.045991756	-0.458109128	0.550092639	1	
D01-B24	-0.042298481	-0.509005211	0.424408249	1	
B01-B00	0.043142411	-0.460958472	0.547243295	1	
D15-B14	0.039008523	-0.427698207	0.505715253	1	
D19-B07	0.04072712	-0.463373763	0.544828004	1	
D19-D14	0.039076115	-0.465024769	0.543176998	1	
D14-B15	0.035099332	-0.431607398	0.501806062	1	
B15-B07	-0.033448327	-0.500155057	0.433258404	1	
D15-B19	-0.035239652	-0.539340535	0.468861232	1	
D00-B03	0.001335335	-0.465371395	0.468042065	1	
B19-B07	0.012543429	-0.491557454	0.516644313	1	
D14-B07	0.001651005	-0.465055725	0.468357736	1	
D15-B07	-0.022696222	-0.489402952	0.444010508	1	
B15-B14	0.028256419	-0.438450311	0.494963149	1	
D15-B15	0.010752104	-0.455954626	0.477458834	1	
D07-B19	0.03189677	-0.472204113	0.535997653	1	
D14-B19	-0.010892424	-0.514993307	0.49320846	1	
D19-B19	0.028183691	-0.510722821	0.567090203	1	
D19-D07	-0.003713079	-0.507813962	0.500387804	1	
D15-D14	-0.024347228	-0.491053958	0.442359502	1	

tableS43 eye_cry2

	diff	lwr	upr	p adj	
D15-B24	-0.218436965	-0.313942476	-0.122931453	1.16E-08	**
B24-B15	0.214352132	0.11884662	0.309857643	1.96E-08	**
D14-B24	-0.214301638	-0.309807149	-0.118796126	1.98E-08	**
B24-B14	0.213858705	0.118353193	0.309364216	2.09E-08	**
D15-B01	-0.208513996	-0.304019508	-0.113008485	4.18E-08	**
B15-B01	-0.204429163	-0.299934674	-0.108923652	7.12E-08	**
D14-B01	-0.204378669	-0.29988418	-0.108873158	7.16E-08	**
B14-B01	-0.203935736	-0.299441247	-0.108430225	7.59E-08	**
D24-D15	0.201021862	0.105516351	0.296527374	1.11E-07	**
D19-B24	-0.197116099	-0.29262161	-0.101610587	1.85E-07	**
D24-B15	0.196937029	0.101431518	0.29244254	1.89E-07	**
D24-D14	0.196886535	0.101381024	0.292392046	1.90E-07	**
D24-B14	0.196443602	0.100938091	0.291949113	2.02E-07	**
D07-B24	-0.190960469	-0.28646598	-0.095454958	4.13E-07	**
D19-B01	-0.18719313	-0.282698642	-0.091687619	6.77E-07	**
D07-B01	-0.181037501	-0.276543012	-0.085531989	1.52E-06	**
D24-D19	0.179700996	0.084195485	0.275206508	1.81E-06	**
B24-B07	0.191286861	0.088129119	0.294444603	2.54E-06	**
D24-D07	0.173545367	0.078039855	0.269050878	4.04E-06	**
D15-D00	-0.172796735	-0.268302247	-0.077291224	4.46E-06	**
D00-B15	0.168711902	0.073206391	0.264217413	7.61E-06	**
D14-D00	-0.168661408	-0.264166919	-0.073155897	7.66E-06	**
D00-B14	0.168218475	0.072712964	0.263723986	8.12E-06	**
B07-B01	-0.181363893	-0.284521635	-0.07820615	8.45E-06	**
D24-B07	0.173871759	0.070714016	0.277029501	2.09E-05	**
D19-D00	-0.151475869	-0.246981381	-0.055970358	7.11E-05	**
D07-D00	-0.14532024	-0.240825751	-0.049814728	0.000156233	**
D15-B03	-0.142150818	-0.237656329	-0.046645306	0.000233422	**
B15-B03	-0.138065985	-0.233571496	-0.042560473	0.000390008	**
D14-B03	-0.138015491	-0.233521002	-0.042509979	0.000392478	**
B14-B03	-0.137572558	-0.233078069	-0.042067046	0.000414813	**
D00-B07	0.145646632	0.042488889	0.248804374	0.000582606	**
D15-B00	-0.134047562	-0.229553073	-0.03854205	0.000642872	**
B15-B00	-0.129962729	-0.22546824	-0.034457217	0.001062023	**
D14-B00	-0.129912235	-0.225417746	-0.034406723	0.00106859	**
B14-B00	-0.129469302	-0.224974813	-0.03396379	0.001127904	**
D15-D01	-0.12904251	-0.224548021	-0.033536998	0.001188076	**
D01-B15	0.124957676	0.029452165	0.220463188	0.00194583	**
D14-D01	-0.124907182	-0.220412694	-0.029401671	0.001957638	**
D01-B14	0.124464249	0.028958738	0.219969761	0.002064226	**
B24-B19	0.123941096	0.028435584	0.219446607	0.002197345	**
D19-B03	-0.120829952	-0.216335463	-0.02532444	0.003177208	**
D07-B03	-0.114674322	-0.210179833	-0.019168811	0.006485295	**
B19-B01	-0.114018127	-0.209523638	-0.018512616	0.006988165	**
D19-B00	-0.112726696	-0.208232207	-0.017221184	0.008087792	**
D15-D03	-0.109873769	-0.20537928	-0.014368257	0.011123648	*
D03-B24	-0.108563196	-0.204068708	-0.013057685	0.01285185	*
D19-D01	-0.107721644	-0.203227155	-0.012216132	0.014090864	*
D07-B00	-0.106571066	-0.202076577	-0.011065555	0.01596596	*
D24-B19	0.106525993	0.011020482	0.202031504	0.016043944	*
B07-B03	-0.115000714	-0.218158456	-0.011842972	0.016141363	*
D03-B15	0.105788935	0.010283424	0.201294447	0.017370502	*
D14-D03	-0.105738441	-0.201243953	-0.01023293	0.01746501	*
D03-B14	0.105295508	0.009789997	0.20080102	0.018314726	*
D07-D01	-0.101566014	-0.197071525	-0.006060502	0.02713933	*
B07-B00	-0.106897458	-0.2100552	-0.003739716	0.035425428	*
D03-B01	-0.098640228	-0.194145739	-0.003134716	0.036618691	*
D15-B19	-0.094495869	-0.190001381	0.001009642	0.055153383	
D01-B07	0.101892406	-0.001265337	0.205050148	0.05602158	
D24-D03	0.091148094	-0.004357418	0.186653605	0.075726597	
B19-B15	0.090411036	-0.005094475	0.185916547	0.081056531	
D14-B19	-0.090360542	-0.185866053	0.005144969	0.081433134	
B19-B14	0.089917609	-0.005587902	0.18542312	0.084801171	
D01-B24	-0.089394455	-0.184899967	0.006111056	0.088931121	
D19-D03	-0.088552903	-0.184058414	0.006952609	0.09593122	
B24-B00	0.084389403	-0.011116108	0.179894914	0.137653588	
D07-D03	-0.082397273	-0.177902784	0.013108239	0.16222237	
D01-B01	-0.079471487	-0.174976998	0.016034025	0.204262216	
D00-B19	0.078300866	-0.017204645	0.173806377	0.223151688	
D03-B07	0.082723665	-0.020434078	0.185881407	0.252976318	
B24-B03	0.076286147	-0.019219364	0.171791658	0.258483164	
B01-B00	0.074466435	-0.021039077	0.169971946	0.29344225	
D19-B19	-0.073175003	-0.168680515	0.022330508	0.319958044	
D24-D01	0.071979353	-0.023526159	0.167484864	0.345713305	
D07-B19	-0.067019374	-0.162524885	0.028486138	0.463187712	
D24-B00	0.066974301	-0.028531211	0.162479812	0.464319147	
B03-B01	-0.066363179	-0.16186869	0.029142333	0.479744038	
D03-D00	-0.062922967	-0.158428478	0.032582545	0.56858901	
B19-B07	0.067345766	-0.035811977	0.170503508	0.583531327	
D24-B03	0.058871044	-0.036634467	0.154376556	0.673147067	
B19-B03	-0.047654949	-0.14316046	0.047850563	0.902375403	
D00-B24	-0.04564023	-0.141145741	0.049865282	0.92818758	
D01-D00	-0.043754226	-0.139259737	0.051751286	0.947791284	
B19-B00	-0.039551692	-0.135057204	0.055953819	0.977348545	
D00-B00	0.038749174	-0.056756338	0.134254685	0.981106201	
D00-B01	-0.035717261	-0.131222772	0.05978825	0.991147299	
D01-B19	0.03454664	-0.060958871	0.130052152	0.993612545	
D03-B03	-0.032277049	-0.127782561	0.063228462	0.996805673	
D00-B03	0.030645918	-0.064859594	0.126151429	0.998160809	
D24-D00	0.028225127	-0.067280384	0.123730638	0.99926252	
D15-D07	-0.027476496	-0.122982007	0.068029016	0.999457953	
D15-B07	-0.027150104	-0.130307846	0.076007639	0.999810234	
D03-B00	-0.024173793	-0.119679305	0.071331718	0.999881827	
D07-B15	0.023391662	-0.072113849	0.118897174	0.999921174	
D14-D07	-0.023341169	-0.11884668	0.072164343	0.999923257	
D07-B14	0.022898236	-0.072607276	0.118403747	0.999939536	
B15-B07	-0.02306527	-0.126223013	0.080092472	0.999974979	
D19-D15	0.021320866	-0.074184645	0.116826377	0.999975475	
D14-B07	-0.023014776	-0.126172519	0.080142966	0.999975671	
B14-B07	-0.022571843	-0.125729586	0.080585899	0.999981041	
D03-D01	-0.019168741	-0.114674252	0.07633677	0.999993847	
D24-B24	-0.017415103	-0.112920614	0.078090409	0.999998289	
D19-B15	0.017236033	-0.078269479	0.112741544	0.999998512	
D19-D14	0.017185539	-0.078319973	0.11269105	0.999998569	
D19-B14	0.016742606	-0.078762906	0.112248117	0.999998996	
D03-B19	0.015377899	-0.080127612	0.110883411	0.999999687	
D01-B03	-0.013108308	-0.10861382	0.082397203	0.999999966	
B24-B01	0.009922968	-0.085582543	0.10542848	0.999999999	
B03-B00	0.008103256	-0.087402255	0.103608767	1	
D24-B01	-0.007492134	-0.102997645	0.088013377	1	
D01-B00	-0.005005052	-0.100510564	0.090500459	1	
D07-B07	0.000326392	-0.10283135	0.103484134	1	
D19-B07	-0.005829238	-0.10898698	0.097328505	1	
B15-B14	-0.000493427	-0.095998938	0.095012084	1	
D14-B14	-0.000442933	-0.095948444	0.095062578	1	
D15-B14	-0.00457826	-0.100083772	0.090927251	1	
D14-B15	5.05E-05	-0.095455017	0.095556005	1	
D15-B15	-0.004084833	-0.099590345	0.091420678	1	
D19-D07	-0.00615563	-0.101661141	0.089349882	1	
D15-D14	-0.004135327	-0.099640839	0.091370184	1	

tableS44 brain_cry4

	diff	lwr	upr	p adj	
D24-D15	-0.109639907	-0.182521291	-0.036758522	0.000211682	**
D24-B15	-0.109298534	-0.182179918	-0.036417149	0.000223728	**
D24-B14	-0.105383527	-0.178264911	-0.032502142	0.00042058	**
D15-B24	0.09777387	0.024892486	0.170655254	0.001402235	**
B24-B15	-0.097432497	-0.170313881	-0.024551113	0.001478821	**
D24-D14	-0.093911764	-0.166793148	-0.021030379	0.002546472	**
B24-B14	-0.09351749	-0.166398874	-0.020636106	0.002704641	**
D15-D01	0.09189681	0.019015425	0.164778194	0.003460117	**
D01-B15	-0.091555437	-0.164436821	-0.018674052	0.003643333	**
D15-B01	0.091469751	0.018588366	0.164351135	0.003690764	**
B15-B01	0.091128378	0.018246993	0.164009762	0.003885686	**
D15-B00	0.098226408	0.019505516	0.1769473	0.003997452	**
B15-B00	0.097885035	0.019164143	0.176605927	0.00419191	**
D01-B14	-0.08764043	-0.160521814	-0.014759045	0.006532715	**
B14-B01	0.087213371	0.014331986	0.160094755	0.006955933	**
B14-B00	0.093970028	0.015249136	0.17269092	0.007177723	**
D15-D03	0.091103348	0.012382456	0.16982424	0.010548688	*
D03-B15	-0.090761975	-0.169482867	-0.012041083	0.011037655	*
D15-D00	0.08328139	0.010400006	0.156162774	0.012281019	*
D00-B15	-0.082940017	-0.155821401	-0.010058633	0.012891281	*
D14-B24	0.082045727	0.009164343	0.154927111	0.014627515	*
D03-B14	-0.086846968	-0.16556786	-0.008126076	0.018394801	*
D00-B14	-0.07902501	-0.151906394	-0.006143626	0.022240001	*
D15-B19	0.085049709	0.006328817	0.163770601	0.023119374	*
B19-B15	-0.084708336	-0.163429228	-0.005987444	0.024134578	*
D14-B00	0.082498265	0.003777373	0.161219157	0.031761264	*
D14-D01	0.076168667	0.003287282	0.149050051	0.03265669	*
D14-B01	0.075741608	0.002860223	0.148622992	0.034549845	*
B19-B14	-0.080793329	-0.159514221	-0.002072437	0.039079466	*
D14-D03	0.075375205	-0.003345687	0.154096097	0.073391052	
D15-B03	0.069092383	-0.003789001	0.141973767	0.079754359	
B15-B03	0.06875101	-0.004130374	0.141632394	0.083066699	
D14-D00	0.067553247	-0.005328137	0.140434631	0.095632402	
B14-B03	0.064836003	-0.008045381	0.137717387	0.130131046	
D14-B19	0.069321566	-0.009399326	0.148042458	0.139834722	
D24-B07	-0.062658889	-0.135540273	0.010222495	0.164514102	
D15-D07	0.060848766	-0.012032618	0.13373015	0.198137491	
D07-B15	-0.060507393	-0.133388777	0.012373991	0.205014175	
D07-B14	-0.056592386	-0.12947377	0.016288998	0.296311547	
D14-B03	0.05336424	-0.019517144	0.126245624	0.388051768	
D19-D15	-0.05623326	-0.134954152	0.022487632	0.428649727	
D19-B15	-0.055891887	-0.134612779	0.022829005	0.438758965	
B24-B07	-0.050792853	-0.123674237	0.022088531	0.469761948	
D24-D19	-0.053406646	-0.132127538	0.025314246	0.514687835	
D24-D07	-0.04879114	-0.121672524	0.024090244	0.536796351	
D19-B14	-0.05197688	-0.130697772	0.026744012	0.559572661	
B07-B00	0.051245391	-0.027475501	0.129966283	0.582650305	
D15-B07	0.046981017	-0.025900367	0.119862401	0.598417099	
B15-B07	0.046639644	-0.02624174	0.119521028	0.610018915	
D14-D07	0.045120623	-0.027760761	0.118002007	0.661110643	
D01-B07	-0.044915792	-0.117797176	0.027965592	0.667900445	
B07-B01	0.044488733	-0.028392651	0.117370117	0.681954313	
B14-B07	0.042724637	-0.030156747	0.115606021	0.738129641	
D03-B07	-0.044122331	-0.122843223	0.034598561	0.793364264	
D24-B03	-0.040547524	-0.113428908	0.03233386	0.801723289	
D19-B24	0.04154061	-0.037180282	0.120261502	0.854872603	
D19-D14	-0.040505117	-0.119226009	0.038215775	0.876303048	
D07-B24	0.036925104	-0.03595628	0.109806488	0.888217046	
D19-B00	0.041993148	-0.042163026	0.126149321	0.899081829	
D00-B07	-0.036300372	-0.109181757	0.036581012	0.900336118	
B19-B07	-0.038068692	-0.116789584	0.0406522	0.918872756	
D07-B00	0.037377642	-0.04134325	0.116098534	0.928930053	
D19-D01	0.035663549	-0.043057343	0.114384441	0.950166036	
D19-B01	0.03523649	-0.043484402	0.113957382	0.954668655	
D14-B07	0.031252874	-0.04162851	0.104134258	0.968018632	
D07-D01	0.031048043	-0.041833341	0.103929427	0.969731857	
D07-B01	0.030620984	-0.0422604	0.103502368	0.973082554	
D19-D03	0.034870088	-0.049286086	0.119026261	0.976115455	
B24-B03	-0.028681488	-0.101562872	0.044199897	0.984905269	
D07-D03	0.030254582	-0.04846631	0.108975474	0.987872104	
B03-B00	0.029134025	-0.049586867	0.107854917	0.991528459	
D24-D00	-0.026358517	-0.099239901	0.046522867	0.993234682	
D19-D00	0.027048129	-0.051672763	0.105769021	0.995959963	
D19-B19	0.028816449	-0.055339725	0.112972622	0.996100127	
D01-B03	-0.022804427	-0.095685811	0.050076957	0.998502427	
D24-B19	-0.024590198	-0.10331109	0.054130695	0.998529736	
D07-D00	0.022432624	-0.050448761	0.095314008	0.998749949	
D07-B19	0.024200943	-0.054519949	0.102921835	0.998766415	
B03-B01	0.022377368	-0.050504016	0.095258752	0.998783565	
B07-B03	0.022111365	-0.050770019	0.09499275	0.998934786	
D03-B03	-0.022010965	-0.100731857	0.056709927	0.999579802	
D24-B01	-0.018170156	-0.09105154	0.054711228	0.99989336	
D24-D01	-0.017743097	-0.090624481	0.055138287	0.999920429	
D24-D03	-0.018536559	-0.097257451	0.060184334	0.999947443	
D15-D14	0.015728143	-0.057153241	0.088609527	0.999982648	
D14-B15	-0.01538677	-0.088268154	0.057494614	0.999986933	
B19-B03	-0.015957326	-0.094678218	0.062763566	0.999992311	
D00-B24	0.014492481	-0.058388904	0.087373865	0.999994028	
D00-B03	-0.014189007	-0.087070391	0.058692377	0.999995485	
D07-B07	-0.013867749	-0.086749133	0.059013635	0.999996669	
D00-B00	0.014945018	-0.063775874	0.09366591	0.999996768	
D19-B03	0.012859122	-0.06586177	0.091580014	0.999999578	
D24-B24	-0.011866036	-0.08474742	0.061015348	0.999999597	
B24-B19	-0.012724161	-0.091445053	0.065996731	0.999999635	
D14-B14	-0.011471763	-0.084353147	0.061409621	0.999999747	
B19-B00	0.013176699	-0.070979474	0.097332872	0.999999765	
D24-B00	-0.011413499	-0.090134391	0.067307394	0.999999919	
D01-D00	-0.00861542	-0.081496804	0.064265964	0.999999996	
D19-B07	-0.009252243	-0.087973135	0.069468649	0.999999996	
D07-B03	0.008243616	-0.064637768	0.081125001	0.999999998	
D00-B01	0.008188361	-0.064693023	0.081069745	0.999999998	
D03-D00	-0.007821958	-0.08654285	0.070898934	1	
D01-B19	-0.0068471	-0.085567993	0.071873792	1	
B24-B01	-0.00630412	-0.079185504	0.066577264	1	
B01-B00	0.006756658	-0.071964235	0.08547755	1	
D03-B24	0.006670522	-0.07205037	0.085391414	1	
D03-B00	0.00712306	-0.077033113	0.091279233	1	
B19-B01	0.006420042	-0.072300851	0.085140934	1	
D01-B24	0.005877061	-0.067004323	0.078758445	1	
D01-B00	0.006329599	-0.072391294	0.085050491	1	
D03-B19	-0.006053639	-0.090209812	0.078102534	1	
B24-B00	0.000452538	-0.078268354	0.07917343	1	
D01-B01	-0.000427059	-0.073308443	0.072454325	1	
D03-B01	0.000366403	-0.07835449	0.079087295	1	
B15-B14	0.003915007	-0.068966377	0.076796391	1	
D15-B14	0.00425638	-0.068625004	0.077137764	1	
D15-B15	0.000341373	-0.072540011	0.073222757	1	
D00-B19	0.001768319	-0.076952573	0.080489211	1	
D03-D01	0.000793461	-0.077927431	0.079514354	1	
D19-D07	0.004615506	-0.074105386	0.083336398	1	

tableS45 eye_cry4

	diff	lwr	upr	p adj	
B14-B00	0.021463108	0.015386192	0.027540024	0	**
B14-B01	0.019757974	0.013681058	0.02583489	0	**
B19-B14	-0.019409205	-0.025486121	-0.013332289	0	**
B24-B14	-0.020846965	-0.026923881	-0.014770049	0	**
D00-B14	-0.020894237	-0.026971153	-0.014817321	0	**
D01-B14	-0.020459749	-0.026536665	-0.014382832	0	**
D24-B14	-0.020727481	-0.026804397	-0.014650565	0	**
D03-B14	-0.018337728	-0.024414644	-0.012260812	3.76E-13	**
D19-B14	-0.015202947	-0.021279863	-0.009126031	8.50E-10	**
B14-B03	0.01496478	0.008887863	0.021041696	1.36E-09	**
D14-B00	0.013699399	0.007622483	0.019776315	1.74E-08	**
B15-B00	0.013350846	0.00727393	0.019427762	3.53E-08	**
D14-D00	0.013130528	0.007053612	0.019207444	5.54E-08	**
D14-B24	0.013083256	0.00700634	0.019160172	6.10E-08	**
D24-D14	-0.012963772	-0.019040688	-0.006886856	7.78E-08	**
D00-B15	-0.012781975	-0.018858891	-0.006705059	1.13E-07	**
B24-B15	-0.012734703	-0.018811619	-0.006657787	1.24E-07	**
D14-D01	0.01269604	0.006619123	0.018772956	1.35E-07	**
D24-B15	-0.012615219	-0.018692135	-0.006538303	1.59E-07	**
D07-B14	-0.012455579	-0.018532495	-0.006378663	2.21E-07	**
D01-B15	-0.012347487	-0.018424403	-0.00627057	2.76E-07	**
D14-B01	0.011994265	0.005917349	0.018071181	5.70E-07	**
B15-B01	0.011645712	0.005568796	0.017722628	1.17E-06	**
D14-B19	0.011645496	0.00556858	0.017722412	1.17E-06	**
B19-B15	-0.011296943	-0.017373859	-0.005220027	2.40E-06	**
D15-B14	-0.011081716	-0.017158632	-0.0050048	3.73E-06	**
D14-D03	0.010574019	0.004497103	0.016650935	1.06E-05	**
D15-B00	0.010381392	0.004304476	0.016458308	1.57E-05	**
B07-B00	0.011057783	0.004493964	0.017621603	2.11E-05	**
D03-B15	-0.010225466	-0.016302382	-0.00414855	2.16E-05	**
D15-D00	0.009812521	0.003735604	0.015889437	5.00E-05	**
D15-B24	0.009765249	0.003688332	0.015842165	5.50E-05	**
D00-B07	-0.010488912	-0.017052731	-0.003925092	6.14E-05	**
B24-B07	-0.01044164	-0.017005459	-0.00387782	6.71E-05	**
D24-D15	-0.009645764	-0.015722681	-0.003568848	7.01E-05	**
B14-B07	0.010405325	0.003841505	0.016969144	7.18E-05	**
D24-B07	-0.010322156	-0.016885975	-0.003758336	8.39E-05	**
D15-D01	0.009378032	0.003301116	0.015454948	0.000120095	**
D01-B07	-0.010054424	-0.016618243	-0.003490604	0.000138017	**
D07-B00	0.009007529	0.002930613	0.015084445	0.000251381	**
D15-B01	0.008676258	0.002599342	0.014753174	0.000482693	**
B07-B01	0.009352649	0.00278883	0.015916469	0.000499388	**
D07-D00	0.008438658	0.002361741	0.014515574	0.000766439	**
D07-B24	0.008391386	0.002314469	0.014468302	0.000839773	**
B19-B07	-0.00900388	-0.015567699	-0.00244006	0.000934459	**
D15-B19	0.008327488	0.002250572	0.014404405	0.000949845	**
D24-D07	-0.008271902	-0.014348818	-0.002194985	0.001056924	**
B15-B14	-0.008112262	-0.014189178	-0.002035346	0.001433818	**
D07-D01	0.008004169	0.001927253	0.014081085	0.001759893	**
D14-B14	-0.007763709	-0.013840625	-0.001686793	0.002762403	**
D19-D14	-0.007439238	-0.013516154	-0.001362322	0.00501454	**
D03-B07	-0.007932403	-0.014496223	-0.001368584	0.005956419	**
D07-B01	0.007302395	0.001225479	0.013379311	0.006418364	**
D15-D03	0.007256012	0.001179096	0.013332928	0.006973741	**
D14-B03	0.007201071	0.001124154	0.013277987	0.007690582	**
D19-B15	-0.007090685	-0.013167601	-0.001013769	0.009346862	**
D07-B19	0.006953626	0.000876709	0.013030542	0.01187249	*
B15-B03	0.006852517	0.000775601	0.012929434	0.014131929	*
B03-B00	0.006498329	0.000421413	0.012575245	0.02559675	*
D19-B00	0.006260161	0.000183245	0.012337078	0.037571797	*
D00-B03	-0.005929457	-0.012006373	0.000147459	0.062520839	
B24-B03	-0.005882185	-0.011959101	0.000194731	0.06707795	
D07-D03	0.005882149	-0.000194767	0.011959065	0.067081577	
D24-B03	-0.005762701	-0.011839617	0.000314215	0.079900652	
D19-D00	0.00569129	-0.000385626	0.011768206	0.08852506	
D19-B24	0.005644018	-0.000432898	0.011720934	0.094657968	
D24-D19	-0.005524534	-0.01160145	0.000552382	0.111765102	
D01-B03	-0.005494969	-0.011571885	0.000581947	0.116371243	
D19-D01	0.005256802	-0.000820114	0.011333718	0.159362115	
B03-B01	0.004793195	-0.001283721	0.010870111	0.27648793	
D14-D07	0.00469187	-0.001385046	0.010768786	0.308228987	
D19-B01	0.004555028	-0.001521889	0.010631944	0.354399295	
B19-B03	-0.004444425	-0.010521341	0.001632491	0.394256531	
D19-B07	-0.004797622	-0.011361441	0.001766198	0.395251913	
D07-B15	-0.004343317	-0.010420233	0.001733599	0.432422206	
B07-B03	0.004559455	-0.002004365	0.011123274	0.480296213	
D19-B19	0.004206258	-0.001870658	0.010283174	0.48627831	
D19-D15	-0.00412123	-0.010198147	0.001955686	0.520579225	
D15-B03	0.003883063	-0.002193853	0.009959979	0.617955833	
D03-B03	-0.003372949	-0.009449865	0.002703968	0.809059024	
D15-D14	-0.003318007	-0.009394923	0.002758909	0.826418016	
D19-D03	0.003134781	-0.002942135	0.009211697	0.878006022	
D03-B00	0.00312538	-0.002951536	0.009202296	0.880377603	
D15-B15	-0.002969454	-0.00904637	0.003107462	0.915658832	
D19-D07	-0.002747368	-0.008824284	0.003329549	0.952944148	
D03-D00	0.002556509	-0.003520407	0.008633425	0.974036677	
D03-B24	0.002509237	-0.003567679	0.008586153	0.977925592	
D07-B03	0.0025092	-0.003567716	0.008586116	0.977928411	
D14-B07	0.002641616	-0.003922204	0.009205435	0.982432842	
D24-D03	-0.002389753	-0.008466669	0.003687163	0.985773402	
B15-B07	0.002293063	-0.004270757	0.008856882	0.995501143	
D03-D01	0.002122021	-0.003954896	0.008198937	0.995521503	
B19-B00	0.002053903	-0.004023013	0.00813082	0.996803256	
D07-B07	-0.002050254	-0.008614074	0.004513565	0.998629401	
B01-B00	0.001705134	-0.004371782	0.00778205	0.999594495	
D00-B19	-0.001485032	-0.007561948	0.004591884	0.999923348	
B24-B19	-0.00143776	-0.007514676	0.004639156	0.9999488	
D03-B01	0.001420246	-0.00465667	0.007497162	0.999956113	
D15-D07	0.001373863	-0.004703053	0.007450779	0.999971185	
D24-B19	-0.001318276	-0.007395192	0.00475864	0.999983034	
D00-B01	-0.001136263	-0.007213179	0.004940654	0.999997602	
B24-B01	-0.001088991	-0.007165907	0.004987926	0.999998647	
D03-B19	0.001071477	-0.005005439	0.007148393	0.999998914	
D01-B19	-0.001050544	-0.00712746	0.005026372	0.99999917	
D01-B00	0.00100336	-0.005073556	0.007080276	0.999999557	
D24-B01	-0.000969506	-0.007046423	0.00510741	0.999999724	
D24-B00	0.000735627	-0.005341289	0.006812544	0.999999994	
D01-B01	-0.000701774	-0.00677869	0.005375142	0.999999997	
D15-B07	-0.000676391	-0.007240211	0.005887428	0.999999999	
B24-B00	0.000616143	-0.005460773	0.00669306	1	
D00-B00	0.000568871	-0.005508045	0.006645788	1	
D01-D00	0.000434488	-0.005642428	0.006511404	1	
B19-B01	0.00034877	-0.005728147	0.006425686	1	
D19-B03	-0.000238167	-0.006315083	0.005838749	1	
D14-B15	0.000348553	-0.005728363	0.006425469	1	
D00-B24	-4.73E-05	-0.006124188	0.006029644	1	
D01-B24	0.000387216	-0.0056897	0.006464132	1	
D24-B24	0.000119484	-0.005957432	0.0061964	1	
D24-D00	0.000166756	-0.00591016	0.006243672	1	
D24-D01	-0.000267732	-0.006344648	0.005809184	1	

tableS46	brain_cry1aa				
	diff	lwr	upr	p adj	
ZT3-ZT19	0.418284906	0.216386188	0.620183624	7.22E-07	**
ZT3-ZT14	0.370980222	0.183161818	0.558798626	2.19E-06	**
ZT3-ZT15	0.353783469	0.165965065	0.541601873	6.23E-06	**
ZT7-ZT3	-0.273731145	-0.461549549	-0.085912741	0.000676726	**
ZT3-ZT24	0.271759002	0.083940598	0.459577406	0.000755198	**
ZT19-ZT1	-0.278873137	-0.47486134	-0.082884935	0.000969209	**
ZT14-ZT1	-0.231568454	-0.41301827	-0.050118637	0.004235325	**
ZT15-ZT1	-0.214371701	-0.395821518	-0.032921884	0.010409266	*
ZT19-ZT0	-0.21639736	-0.418296077	-0.014498642	0.027599647	*
ZT3-ZT0	0.201887546	0.007909533	0.39586556	0.035848502	*
ZT14-ZT0	-0.169092676	-0.35691108	0.018725728	0.106530758	
ZT15-ZT0	-0.151895923	-0.339714327	0.035922481	0.196770121	
ZT24-ZT19	0.146525904	-0.049462299	0.342514106	0.282181261	
ZT3-ZT1	0.139411768	-0.048406636	0.327230173	0.290655922	
ZT7-ZT1	-0.134319376	-0.315769193	0.04713044	0.293879701	
ZT7-ZT19	0.144553761	-0.051434441	0.340541963	0.298226831	
ZT24-ZT1	-0.132347234	-0.313797051	0.049102583	0.311661371	
ZT24-ZT14	0.09922122	-0.082228597	0.280671037	0.670320624	
ZT7-ZT14	0.097249077	-0.08420074	0.278698894	0.692017043	
ZT24-ZT15	0.082024467	-0.09942535	0.263474284	0.840401932	
ZT7-ZT15	0.080052325	-0.101397492	0.261502141	0.856296451	
ZT7-ZT0	-0.071843599	-0.259662003	0.115974805	0.925988872	
ZT24-ZT0	-0.069871456	-0.25768986	0.117946948	0.935549929	
ZT1-ZT0	0.062475778	-0.125342626	0.250294182	0.963974296	
ZT19-ZT15	-0.064501436	-0.260489639	0.131486766	0.965986313	
ZT19-ZT14	-0.047304684	-0.243292886	0.148683518	0.994330155	
ZT15-ZT14	0.017196753	-0.164253064	0.198646569	0.999987918	
ZT7-ZT24	-0.001972143	-0.183421959	0.179477674	1	

tableS47	eye_cry1aa				
	diff	lwr	upr	p adj	
ZT3-ZT19	0.083043015	0.045900627	0.120185404	8.77E-08	**
ZT3-ZT15	0.079254023	0.042111635	0.116396411	2.93E-07	**
ZT19-ZT1	-0.075141525	-0.112283914	-0.037999137	1.08E-06	**
ZT15-ZT1	-0.071352533	-0.108494921	-0.034210145	3.57E-06	**
ZT3-ZT14	0.068484777	0.031342388	0.105627165	8.74E-06	**
ZT14-ZT1	-0.060583287	-0.097725675	-0.023440898	9.79E-05	**
ZT19-ZT0	-0.05358955	-0.090731938	-0.016447162	0.000757188	**
ZT15-ZT0	-0.049800558	-0.086942946	-0.012658169	0.002177443	**
ZT7-ZT3	-0.046899152	-0.085345176	-0.008453127	0.007164248	**
ZT24-ZT19	0.044850442	0.007708054	0.081992831	0.008055515	**
ZT24-ZT15	0.04106145	0.003919062	0.078203838	0.020500497	*
ZT14-ZT0	-0.039031311	-0.0761737	-0.001888923	0.032871884	*
ZT3-ZT24	0.038192573	0.001050185	0.075334961	0.039698697	*
ZT7-ZT1	-0.038997662	-0.077443686	-0.000551637	0.044511058	*
ZT7-ZT19	0.036143864	-0.002302161	0.074589888	0.079750357	
ZT7-ZT15	0.032354871	-0.006091153	0.070800896	0.160192126	
ZT24-ZT14	0.030292204	-0.006850185	0.067434592	0.189465359	
ZT24-ZT1	-0.030291083	-0.067433471	0.006851305	0.189501479	
ZT3-ZT0	0.029453466	-0.007688923	0.066595854	0.217918926	
ZT1-ZT0	0.021551975	-0.015590413	0.058694364	0.604303767	
ZT7-ZT14	0.021585625	-0.016860399	0.060031649	0.643067201	
ZT7-ZT0	-0.017445686	-0.055891711	0.021000338	0.839561109	
ZT19-ZT14	-0.014558239	-0.051700627	0.02258415	0.917755107	
ZT15-ZT14	-0.010769246	-0.047911635	0.026373142	0.983498965	
ZT24-ZT0	-0.008739108	-0.045881496	0.028403281	0.995243468	
ZT7-ZT24	-0.008706579	-0.047152603	0.029739446	0.996243702	
ZT3-ZT1	0.00790149	-0.029240898	0.045043878	0.99745901	
ZT19-ZT15	-0.003788992	-0.040931381	0.033353396	0.999980468	

tableS48	brain_cry1ab				
	diff	lwr	upr	p adj	
ZT3-ZT19	0.263677832	0.16112446	0.366231204	2.28E-09	**
ZT3-ZT15	0.242210159	0.146808807	0.337611511	3.31E-09	**
ZT3-ZT14	0.235237983	0.139836631	0.330639335	7.66E-09	**
ZT19-ZT1	-0.214651744	-0.314202901	-0.115100586	2.73E-07	**
ZT15-ZT1	-0.193184071	-0.285350533	-0.101017608	5.46E-07	**
ZT14-ZT1	-0.186211895	-0.278378358	-0.094045432	1.30E-06	**
ZT7-ZT3	-0.191390983	-0.286792335	-0.095989631	1.54E-06	**
ZT3-ZT24	0.179389172	0.08398782	0.274790524	6.47E-06	**
ZT19-ZT0	-0.177456416	-0.280009788	-0.074903044	3.50E-05	**
ZT15-ZT0	-0.155988743	-0.251390095	-0.060587391	0.000100578	**
ZT14-ZT0	-0.149016567	-0.244417919	-0.053615215	0.000222664	**
ZT7-ZT1	-0.142364895	-0.234531357	-0.050198432	0.000268347	**
ZT24-ZT1	-0.130363084	-0.222529546	-0.038196621	0.001057912	**
ZT7-ZT0	-0.105169567	-0.200570919	-0.009768214	0.021191037	*
ZT24-ZT0	-0.093167756	-0.188569108	0.002233597	0.060171317	
ZT3-ZT0	0.086221416	-0.012308677	0.184751509	0.127194315	
ZT24-ZT19	0.08428866	-0.015262497	0.183839818	0.154146424	
ZT7-ZT19	0.072286849	-0.027264308	0.171838007	0.317115245	
ZT24-ZT15	0.062820987	-0.029345475	0.15498745	0.396773969	
ZT24-ZT14	0.055848811	-0.036317651	0.148015274	0.548386211	
ZT7-ZT15	0.050819176	-0.041347286	0.142985639	0.661113061	
ZT3-ZT1	0.049026088	-0.046375264	0.14442744	0.7347116	
ZT7-ZT14	0.043847	-0.048319462	0.136013463	0.802789508	
ZT1-ZT0	0.037195328	-0.058206024	0.13259668	0.918758683	
ZT19-ZT14	-0.028439849	-0.127991006	0.071111309	0.98457641	
ZT19-ZT15	-0.021467673	-0.12101883	0.078083484	0.99717576	
ZT7-ZT24	-0.012001811	-0.104168273	0.080164652	0.999895324	
ZT15-ZT14	-0.006972176	-0.099138638	0.085194287	0.999997439	

tableS49	eye_cry1ab				
	diff	lwr	upr	p adj	
ZT19-ZT1	-0.052586615	-0.0721514	-0.03302183	4.24E-10	**
ZT3-ZT19	0.042172703	0.022607918	0.061737487	2.27E-07	**
ZT15-ZT1	-0.040050805	-0.05961559	-0.02048602	8.15E-07	**
ZT7-ZT1	-0.035375755	-0.055627231	-0.015124279	2.66E-05	**
ZT19-ZT0	-0.032652209	-0.052216994	-0.013087424	6.42E-05	**
ZT19-ZT14	-0.032156907	-0.051721691	-0.012592122	8.52E-05	**
ZT24-ZT19	0.032015143	0.012450358	0.051579928	9.23E-05	**
ZT3-ZT15	0.029636893	0.010072108	0.049201678	0.000350912	**
ZT7-ZT3	-0.024961843	-0.045213319	-0.004710366	0.006342944	**
ZT24-ZT1	-0.020571472	-0.040136257	-0.001006687	0.032706389	*
ZT14-ZT1	-0.020429709	-0.039994493	-0.000864924	0.034763627	*
ZT15-ZT0	-0.0201164	-0.039681184	-0.000551615	0.03972507	*
ZT1-ZT0	0.019934406	0.000369621	0.039499191	0.042887138	*
ZT15-ZT14	-0.019621097	-0.039185882	-5.63E-05	0.048853635	*
ZT24-ZT15	0.019479334	-8.55E-05	0.039044118	0.051784526	
ZT7-ZT19	0.01721086	-0.003040616	0.037462336	0.151618426	
ZT7-ZT0	-0.015441349	-0.035692826	0.004810127	0.2609077	
ZT7-ZT14	-0.014946047	-0.035197523	0.00530543	0.299104997	
ZT7-ZT24	-0.014804283	-0.03505576	0.005447193	0.310630523	
ZT19-ZT15	-0.01253581	-0.032100595	0.007028975	0.479264854	
ZT3-ZT1	-0.010413912	-0.029978697	0.009150872	0.701632302	
ZT3-ZT24	0.010157559	-0.009407225	0.029722344	0.726970775	
ZT3-ZT14	0.010015796	-0.009548989	0.029580581	0.74065631	
ZT3-ZT0	0.009520493	-0.010044291	0.029085278	0.786310974	
ZT7-ZT15	0.00467505	-0.015576426	0.024926526	0.995769885	
ZT24-ZT0	-0.000637066	-0.020201851	0.018927719	0.999999993	
ZT14-ZT0	-0.000495303	-0.020060088	0.019069482	0.999999999	
ZT24-ZT14	-0.000141763	-0.019706548	0.019423022	1	

tableS50	brain_cry1ba				
	diff	lwr	upr	p adj	
ZT24-ZT15	-0.586967341	-0.89698889	-0.276945792	6.39E-06	**
ZT15-ZT0	0.514877477	0.204855928	0.824899026	8.24E-05	**
ZT19-ZT15	-0.522146903	-0.844827562	-0.199466244	0.00013041	**
ZT15-ZT1	0.499464152	0.189442603	0.809485701	0.000140712	**
ZT24-ZT14	-0.460789069	-0.760966143	-0.160611995	0.000315582	**
ZT14-ZT0	0.388699205	0.088522131	0.688876279	0.003615142	**
ZT19-ZT14	-0.395968631	-0.709202925	-0.082734338	0.004862416	**
ZT14-ZT1	0.37328588	0.073108806	0.673462954	0.005913785	**
ZT7-ZT24	0.331780508	0.031603434	0.631957582	0.020793949	*
ZT3-ZT15	-0.294674433	-0.604695982	0.015347116	0.073410812	
ZT3-ZT24	0.292292908	-0.017728641	0.602314457	0.077779201	
ZT7-ZT0	0.259690644	-0.04048643	0.559867719	0.135537772	
ZT7-ZT19	0.26696007	-0.046274223	0.580194364	0.147781862	
ZT7-ZT15	-0.255186833	-0.555363907	0.044990241	0.149910025	
ZT7-ZT1	0.244277319	-0.055899755	0.544454393	0.189590999	
ZT3-ZT0	0.220203044	-0.089818505	0.530224593	0.342318212	
ZT3-ZT19	0.22747247	-0.095208189	0.550153129	0.351733234	
ZT3-ZT1	0.204789718	-0.105231831	0.514811267	0.434858417	
ZT3-ZT14	-0.168496161	-0.468673236	0.131680913	0.638107796	
ZT7-ZT14	-0.129008561	-0.419007166	0.160990044	0.849138563	
ZT15-ZT14	0.126178272	-0.173998802	0.426355346	0.882616782	
ZT24-ZT1	-0.087503189	-0.397524738	0.22251836	0.985325656	
ZT24-ZT0	-0.072089864	-0.382111413	0.237931685	0.995387223	
ZT24-ZT19	-0.064820438	-0.387501097	0.257860221	0.998153021	
ZT7-ZT3	0.039487601	-0.260689474	0.339664675	0.999885049	
ZT19-ZT1	-0.022682751	-0.34536341	0.299997908	0.999998416	
ZT1-ZT0	0.015413325	-0.294608224	0.325434874	0.999999857	
ZT19-ZT0	-0.007269426	-0.329950085	0.315411233	0.999999999	

tableS51	eye_cry1ba				
	diff	lwr	upr	p adj	
ZT14-ZT0	0.515597529	0.326153537	0.705041521	3.20E-10	**
ZT24-ZT14	-0.511861234	-0.701305227	-0.322417242	4.02E-10	**
ZT14-ZT1	0.498134921	0.308690929	0.687578913	9.33E-10	**
ZT3-ZT14	-0.463005695	-0.652449687	-0.273561703	8.15E-09	**
ZT15-ZT0	0.468547927	0.272454765	0.664641089	1.55E-08	**
ZT19-ZT14	-0.449119788	-0.63856378	-0.259675796	1.93E-08	**
ZT24-ZT15	-0.464811632	-0.660904794	-0.26871847	1.93E-08	**
ZT15-ZT1	0.451085319	0.254992157	0.64717848	4.40E-08	**
ZT3-ZT15	-0.415956093	-0.612049254	-0.219862931	3.61E-07	**
ZT19-ZT15	-0.402070186	-0.598163348	-0.205977024	8.27E-07	**
ZT7-ZT14	-0.377533856	-0.573627018	-0.181440694	3.54E-06	**
ZT7-ZT15	-0.330484254	-0.5330084	-0.127960107	9.94E-05	**
ZT7-ZT0	0.138063673	-0.058029489	0.334156835	0.356716378	
ZT7-ZT24	0.134327378	-0.061765783	0.33042054	0.391747796	
ZT7-ZT1	0.120601065	-0.075492097	0.316694227	0.531253144	
ZT7-ZT3	0.085471839	-0.110621323	0.281565001	0.86480009	
ZT7-ZT19	0.071585932	-0.12450723	0.267679094	0.94198398	
ZT19-ZT0	0.066477741	-0.122966251	0.255921733	0.952682702	
ZT24-ZT19	-0.062741446	-0.252185438	0.126702546	0.965190432	
ZT3-ZT0	0.052591834	-0.136852158	0.242035826	0.987095077	
ZT19-ZT1	0.049015133	-0.140428859	0.238459125	0.991486643	
ZT3-ZT24	0.04885554	-0.140588453	0.238299532	0.991651046	
ZT15-ZT14	-0.047049602	-0.243142764	0.149043559	0.994603785	
ZT3-ZT1	0.035129226	-0.154314766	0.224573218	0.998934881	
ZT1-ZT0	0.017462608	-0.171981384	0.2069066	0.999990171	
ZT3-ZT19	-0.013885907	-0.203329899	0.175558085	0.999997973	
ZT24-ZT1	-0.013726313	-0.203170306	0.175717679	0.999998129	
ZT24-ZT0	0.003736295	-0.185707697	0.193180287	1	

tableS52	brain_cry1bb				
	diff	lwr	upr	p adj	
ZT24-ZT15	-0.228753987	-0.370449243	-0.08705873	0.000129137	**
ZT15-ZT1	0.217172214	0.080281591	0.354062837	0.000174721	**
ZT15-ZT0	0.218756578	0.077061321	0.360451834	0.00027602	**
ZT24-ZT14	-0.190960151	-0.332655408	-0.049264895	0.002108953	**
ZT19-ZT15	-0.196985783	-0.344844555	-0.049127011	0.002459892	**
ZT3-ZT15	-0.185881556	-0.327576813	-0.0441863	0.003011018	**
ZT14-ZT1	0.179378379	0.042487755	0.316269002	0.003054741	**
ZT14-ZT0	0.180962742	0.039267486	0.322657999	0.00422807	**
ZT7-ZT15	-0.150083244	-0.286973867	-0.013192621	0.022374528	*
ZT19-ZT14	-0.159191948	-0.30705072	-0.011333176	0.026518716	*
ZT3-ZT14	-0.148087721	-0.289782977	-0.006392464	0.034603225	*
ZT7-ZT14	-0.112289409	-0.249180032	0.024601214	0.182648199	
ZT7-ZT24	0.078670743	-0.063024514	0.220365999	0.652466024	
ZT7-ZT1	0.06708897	-0.069801653	0.203979593	0.777416106	
ZT7-ZT0	0.068673333	-0.073021923	0.21036859	0.786908855	
ZT7-ZT19	0.046902539	-0.100956233	0.194761311	0.972007929	
ZT3-ZT24	0.04287243	-0.103469801	0.189214662	0.982070874	
ZT15-ZT14	0.037793836	-0.099096787	0.174684459	0.987265899	
ZT7-ZT3	0.035798312	-0.105896944	0.177493569	0.992472876	
ZT3-ZT0	0.032875021	-0.11346721	0.179217253	0.996328664	
ZT3-ZT1	0.031290658	-0.110404599	0.172985914	0.996699602	
ZT24-ZT19	-0.031768203	-0.184086027	0.120549621	0.997693833	
ZT19-ZT0	0.021770794	-0.13054703	0.174088618	0.999803079	
ZT19-ZT1	0.020186431	-0.127672341	0.168045203	0.99985505	
ZT24-ZT1	-0.011581773	-0.153277029	0.130113484	0.999995593	
ZT3-ZT19	0.011104227	-0.141213597	0.163422051	0.999997999	
ZT24-ZT0	-0.009997409	-0.156339641	0.136344823	0.999998724	
ZT1-ZT0	0.001584364	-0.140110893	0.14327962	1	

tableS53	eye_cry1bb				
	diff	lwr	upr	p adj	
ZT14-ZT0	0.103819052	0.055804756	0.151833349	2.38E-07	**
ZT14-ZT1	0.101682186	0.05366789	0.149696483	3.99E-07	**
ZT24-ZT14	-0.1029934	-0.152692919	-0.053293882	6.77E-07	**
ZT19-ZT14	-0.095764313	-0.145463831	-0.046064794	3.64E-06	**
ZT3-ZT14	-0.090438457	-0.138452753	-0.04242416	5.98E-06	**
ZT15-ZT0	0.081016266	0.03300197	0.129030563	5.50E-05	**
ZT15-ZT1	0.0788794	0.030865104	0.126893697	9.01E-05	**
ZT24-ZT15	-0.080190614	-0.129890133	-0.030491096	0.000124435	**
ZT19-ZT15	-0.072961527	-0.122661045	-0.023262008	0.000596322	**
ZT3-ZT15	-0.067635671	-0.115649967	-0.019621374	0.001109107	**
ZT7-ZT14	-0.069777225	-0.119476743	-0.020077706	0.001163984	**
ZT7-ZT15	-0.046974439	-0.096673957	0.00272508	0.076655813	
ZT7-ZT0	0.034041828	-0.015657691	0.083741346	0.391183349	
ZT7-ZT24	0.033216176	-0.018113266	0.084545618	0.464860493	
ZT7-ZT1	0.031904962	-0.017794557	0.08160448	0.475257221	
ZT7-ZT19	0.025987088	-0.025342354	0.07731653	0.749702344	
ZT15-ZT14	-0.022802786	-0.070817082	0.02521151	0.804834924	
ZT7-ZT3	0.020661232	-0.029038286	0.070360751	0.89040705	
ZT3-ZT0	0.013380596	-0.034633701	0.061394892	0.98672349	
ZT3-ZT24	0.012554944	-0.037144575	0.062254462	0.992577582	
ZT3-ZT1	0.01124373	-0.036770567	0.059258026	0.995320182	
ZT19-ZT0	0.00805474	-0.041644779	0.057754258	0.999554206	
ZT24-ZT19	-0.007229088	-0.05855853	0.044100354	0.999824342	
ZT19-ZT1	0.005917873	-0.043781645	0.055617392	0.999943222	
ZT3-ZT19	0.005325856	-0.044373662	0.055025375	0.999972252	
ZT1-ZT0	0.002136866	-0.04587743	0.050151163	0.999999936	
ZT24-ZT1	-0.001311214	-0.051010733	0.048388304	0.999999998	
ZT24-ZT0	0.000825652	-0.048873867	0.05052517	1	

tableS54	brain_cry2				
	diff	lwr	upr	p adj	
ZT14-ZT0	-0.457278146	-0.752631528	-0.161924764	0.000258195	**
ZT15-ZT0	-0.455323551	-0.750676933	-0.159970169	0.000277205	**
ZT14-ZT1	-0.436305491	-0.721643966	-0.150967015	0.000317052	**
ZT15-ZT1	-0.434350895	-0.719689371	-0.14901242	0.000341117	**
ZT7-ZT0	-0.405031177	-0.700384559	-0.109677795	0.001644153	**
ZT7-ZT1	-0.384058521	-0.669396997	-0.098720046	0.00212421	**
ZT19-ZT0	-0.400616002	-0.718111347	-0.083120656	0.004870431	**
ZT19-ZT1	-0.379643346	-0.687844125	-0.071442568	0.006492712	**
ZT3-ZT14	0.362685682	0.0673323	0.658039064	0.006733443	**
ZT3-ZT15	0.360731087	0.065377704	0.656084469	0.007168929	**
ZT7-ZT3	-0.310438713	-0.605792095	-0.01508533	0.0328938	*
ZT3-ZT19	0.306023538	-0.011471808	0.623518883	0.066414943	
ZT24-ZT14	0.267298177	-0.018040298	0.552636653	0.081585284	
ZT24-ZT15	0.265343582	-0.019994894	0.550682057	0.085854915	
ZT7-ZT24	-0.215051208	-0.500389683	0.070287268	0.272806943	
ZT24-ZT19	0.210636033	-0.097564746	0.518836811	0.39331683	
ZT24-ZT0	-0.189979969	-0.485333351	0.105373413	0.47188385	
ZT24-ZT1	-0.169007313	-0.454345789	0.116331162	0.576772558	
ZT3-ZT24	0.095387505	-0.199965877	0.390740887	0.969295621	
ZT3-ZT0	-0.094592464	-0.399632126	0.210447197	0.975454798	
ZT3-ZT1	-0.073619809	-0.368973191	0.221733574	0.993107386	
ZT19-ZT14	0.056662145	-0.251538634	0.364862923	0.998977679	
ZT7-ZT14	0.05224697	-0.233091506	0.337585445	0.999004089	
ZT19-ZT15	0.054707549	-0.253493229	0.362908328	0.999185668	
ZT7-ZT15	0.050292374	-0.235046102	0.33563085	0.999222261	
ZT1-ZT0	-0.020972656	-0.316326038	0.274380727	0.999998346	
ZT7-ZT19	-0.004415175	-0.312615954	0.303785603	1	
ZT15-ZT14	0.001954596	-0.28338388	0.287293071	1	

tableS55	eye_cry2				
	diff	lwr	upr	p adj	
ZT24-ZT15	0.207686997	0.142352524	0.27302147	5.15E-12	**
ZT24-ZT14	0.20537262	0.140038146	0.270707093	5.88E-12	**
ZT7-ZT24	-0.1823928	-0.250020405	-0.114765195	3.81E-10	**
ZT15-ZT1	-0.166735836	-0.23207031	-0.101401363	2.08E-09	**
ZT14-ZT1	-0.164421459	-0.229755932	-0.099086986	3.16E-09	**
ZT24-ZT19	0.151821046	0.086486573	0.217155519	3.09E-08	**
ZT15-ZT0	-0.151379732	-0.216714205	-0.086045259	3.35E-08	**
ZT14-ZT0	-0.149065355	-0.214399828	-0.083730881	5.10E-08	**
ZT7-ZT1	-0.141441639	-0.209069244	-0.073814035	4.83E-07	**
ZT3-ZT15	0.123969877	0.058635403	0.18930435	4.69E-06	**
ZT7-ZT0	-0.126085535	-0.19371314	-0.05845793	6.89E-06	**
ZT3-ZT14	0.121655499	0.056321026	0.186989973	7.08E-06	**
ZT19-ZT1	-0.110869885	-0.176204359	-0.045535412	4.68E-05	**
ZT19-ZT0	-0.095513781	-0.160848254	-0.030179308	0.00061848	**
ZT7-ZT3	-0.09867568	-0.166303285	-0.031048075	0.000637259	**
ZT3-ZT24	-0.08371712	-0.149051594	-0.018382647	0.003945823	**
ZT3-ZT19	0.068103926	0.002769452	0.133438399	0.035298009	*
ZT24-ZT0	0.056307265	-0.009027208	0.121641738	0.139866686	
ZT19-ZT15	0.055865951	-0.009468522	0.121200424	0.146405474	
ZT19-ZT14	0.053551574	-0.011782899	0.118886047	0.184623608	
ZT3-ZT1	-0.04276596	-0.108100433	0.022568514	0.451457592	
ZT24-ZT1	0.040951161	-0.024383313	0.106285634	0.507735832	
ZT7-ZT19	-0.030571754	-0.098199359	0.037055851	0.842126262	
ZT3-ZT0	-0.027409855	-0.092744329	0.037924618	0.886632165	
ZT7-ZT15	0.025294197	-0.042333408	0.092921802	0.934722317	
ZT7-ZT14	0.02297982	-0.044647785	0.090607425	0.960260202	
ZT1-ZT0	0.015356104	-0.049978369	0.080690578	0.995274242	
ZT15-ZT14	-0.002314377	-0.06764885	0.063020096	0.999999987	

tableS56	brain_cry4				
	diff	lwr	upr	p adj	
ZT24-ZT15	-0.103536202	-0.146696481	-0.060375922	1.65E-08	**
ZT24-ZT14	-0.093714627	-0.136874906	-0.050554347	2.29E-07	**
ZT15-ZT1	0.091512594	0.048352314	0.134672873	4.13E-07	**
ZT15-ZT0	0.089515711	0.044840577	0.134190845	1.58E-06	**
ZT14-ZT1	0.081691019	0.038530739	0.124851298	5.62E-06	**
ZT14-ZT0	0.079694136	0.035019002	0.12436927	1.93E-05	**
ZT3-ZT15	-0.078354967	-0.123030101	-0.033679833	2.70E-05	**
ZT3-ZT14	-0.068533392	-0.113208526	-0.023858258	0.000300673	**
ZT19-ZT15	-0.070470798	-0.117089228	-0.023852368	0.000381818	**
ZT19-ZT14	-0.060649223	-0.107267653	-0.014030793	0.00332373	**
ZT7-ZT15	-0.053744205	-0.096904485	-0.010583926	0.005712386	**
ZT7-ZT24	0.049791997	0.006631717	0.092952276	0.013422456	*
ZT7-ZT14	-0.04392263	-0.08708291	-0.000762351	0.043356282	*
ZT7-ZT1	0.037768388	-0.005391891	0.080928668	0.127197957	
ZT7-ZT0	0.035771506	-0.008903628	0.08044664	0.206807292	
ZT24-ZT19	-0.033065404	-0.079683834	0.013553026	0.346163845	
ZT3-ZT24	0.025181235	-0.019493899	0.069856369	0.636093409	
ZT7-ZT3	0.024610762	-0.020064372	0.069285896	0.66212922	
ZT19-ZT1	0.021041795	-0.025576635	0.067660225	0.841434761	
ZT19-ZT0	0.019044913	-0.028979413	0.067069239	0.911819368	
ZT7-ZT19	0.016726593	-0.029891837	0.063345023	0.946367209	
ZT24-ZT0	-0.014020491	-0.058695625	0.030654643	0.97377005	
ZT3-ZT1	0.013157626	-0.031517507	0.05783276	0.981636288	
ZT24-ZT1	-0.012023608	-0.055183888	0.031136671	0.986668085	
ZT3-ZT0	0.011160744	-0.034979536	0.057301024	0.994254985	
ZT15-ZT14	0.009821575	-0.033338705	0.052981855	0.996051499	
ZT3-ZT19	-0.007884169	-0.055908495	0.040140157	0.99951112	
ZT1-ZT0	-0.001996882	-0.046672016	0.042678251	0.999999934	

tableS57	eye_cry4				
	diff	lwr	upr	p adj	
ZT14-ZT0	0.017296818	0.012689226	0.02190441	3.63409E-12	**
ZT24-ZT14	-0.016905368	-0.02151296	-0.012297776	3.64409E-12	**
ZT14-ZT1	0.016227007	0.011619415	0.020834599	3.6805E-12	**
ZT19-ZT14	-0.013424221	-0.018031813	-0.008816629	3.41359E-11	**
ZT3-ZT14	-0.012769399	-0.017376991	-0.008161807	1.62029E-10	**
ZT15-ZT0	0.011581683	0.006974092	0.016189275	3.27071E-09	**
ZT24-ZT15	-0.011190234	-0.015797825	-0.006582642	8.92607E-09	**
ZT15-ZT1	0.010511872	0.00590428	0.015119464	5.10697E-08	**
ZT7-ZT0	0.009601774	0.004832463	0.014371084	1.21351E-06	**
ZT7-ZT24	0.009210324	0.004441013	0.013979635	3.17091E-06	**
ZT7-ZT1	0.008531963	0.003762652	0.013301273	1.64358E-05	**
ZT19-ZT15	-0.007709087	-0.012316678	-0.003101495	6.12025E-05	**
ZT7-ZT14	-0.007695044	-0.012464355	-0.002925734	0.000119143	**
ZT3-ZT15	-0.007054265	-0.011661856	-0.002446673	0.000294446	**
ZT15-ZT14	-0.005715135	-0.010322726	-0.001107543	0.005885308	**
ZT7-ZT19	0.005729177	0.000959866	0.010498488	0.008546935	**
ZT7-ZT3	0.005074355	0.000305044	0.009843666	0.029411088	*
ZT3-ZT0	0.004527419	-8.02E-05	0.009135011	0.057443686	
ZT3-ZT24	0.004135969	-0.000471623	0.008743561	0.108953142	
ZT19-ZT0	0.003872597	-0.000734995	0.008480189	0.161340325	
ZT24-ZT19	-0.003481147	-0.008088739	0.001126445	0.271438168	
ZT3-ZT1	0.003457608	-0.001149984	0.008065199	0.279342184	
ZT19-ZT1	0.002802786	-0.001804806	0.007410377	0.545983449	
ZT7-ZT15	-0.00197991	-0.00674922	0.002789401	0.892005184	
ZT1-ZT0	0.001069811	-0.003537781	0.005677403	0.995616682	
ZT24-ZT1	-0.000678361	-0.005285953	0.00392923	0.999767301	
ZT3-ZT19	0.000654822	-0.00395277	0.005262414	0.99981611	
ZT24-ZT0	0.00039145	-0.004216142	0.004999042	0.999994431	

Table S58 Two-way ANOVA for data shown in fig5#, $

ZT vs light condition			light condition			ZT		
Degree of freedom	F value	*p* value	Degree of freedom	F value	*p* value	Degree of freedom	F value	*p* value
(36, 175)	17.43	<2e-16	(3, 175)	0.34	7.96E-01	(12, 175)	20.72	<2e-16

#, Result of post hoc analysis for all light conditions is in tableS59.
$, Result of post hoc analysis for each light condition is in tableS60-S63.

tableS59_fig5_posthoc

		diff	lwr	upr	p adj	
ESDzt5	SDzt15	0.0813205	0.0588506	0.10379	0	**
ESDzt5	SDzt17	0.0806796	0.0582096	0.10315	0	**
ESDzt5	LDzt17	0.0806189	0.0563486	0.10489	0	**
ESDzt5	ESDzt13	0.0801186	0.0576486	0.10259	0	**
ESDzt5	SDzt13	0.0799464	0.0586296	0.10126	0	**
ESDzt5	ESDzt11	0.079543	0.0582262	0.10086	0	**
ESDzt5	ESDzt9	0.078132	0.0568151	0.09945	0	**
ESDzt5	ELDzt19	0.0781287	0.0556588	0.1006	0	**
ESDzt5	ESDzt15	0.0771784	0.0558616	0.0985	0	**
ESDzt5	ELDzt21	0.0764342	0.0551174	0.09775	0	**
ESDzt5	LDzt19	0.0762859	0.0520156	0.10056	0	**
ESDzt5	ELDzt17	0.0738122	0.0524953	0.09513	0	**
ESDzt5	SDzt19	0.0724098	0.0499399	0.09488	0	**
ESDzt5	ELDzt23	0.07145	0.04898	0.09392	0	**
ESDzt5	LDzt21	0.0701076	0.0487907	0.09142	0	**
ESDzt5	ESDzt17	0.0659275	0.0446107	0.08724	0	**
ESDzt5	ESDzt25	0.0651087	0.0437918	0.08643	0	**
ESDzt5	LDzt5	0.0643248	0.0430079	0.08564	0	**
ESDzt5	LDzt15	0.0719254	0.0476551	0.0962	1.34E-14	**
ESDzt5	ELDzt7	0.0652854	0.0428155	0.08776	3.76E-14	**
ESDzt5	ELDzt9	0.0613781	0.0400613	0.0827	5.67E-14	**
ESDzt5	LDzt9	0.0604513	0.0391344	0.08177	9.00E-14	**
ESDzt5	ESDzt1	0.0591616	0.0378448	0.08048	1.57E-13	**
ESDzt5	ESDzt23	0.0585578	0.0372409	0.07987	1.94E-13	**
ESDzt5	ELDzt25	0.0583606	0.0370437	0.07968	1.98E-13	**
ESDzt5	LDzt7	0.0612729	0.0388029	0.08374	2.17E-13	**
ESDzt5	LDzt23	0.0574123	0.0360955	0.07873	2.53E-13	**
ESDzt5	LDzt11	0.0573138	0.0359969	0.07863	2.56E-13	**
ESDzt5	SDzt25	0.0600072	0.0375372	0.08248	2.89E-13	**
ESDzt5	ELDzt1	0.0567871	0.0354703	0.0781	2.96E-13	**
ESDzt5	ELDzt11	0.0589333	0.0364633	0.0814	3.42E-13	**
ESDzt5	SDzt23	0.0579482	0.0354783	0.08042	3.78E-13	**
ESDzt5	SDzt01	0.0552702	0.0339533	0.07659	4.07E-13	**
ESDzt5	ESDzt19	0.054296	0.0329791	0.07561	4.32E-13	**
ESDzt5	ELDzt13	0.0506592	0.0293423	0.07198	4.51E-13	**
ESDzt5	SDzt03	0.0540457	0.0315758	0.07652	4.63E-13	**
SDzt09	SDzt13	0.0560404	0.0328336	0.07925	4.67E-13	**
LDzt13	SDzt13	0.0539186	0.0307118	0.07713	4.68E-13	**
SDzt09	ESDzt11	0.055637	0.0324302	0.07884	4.68E-13	**
SDzt09	SDzt15	0.0574145	0.0331442	0.08168	4.69E-13	**
SDzt09	SDzt17	0.0567736	0.0325033	0.08104	4.83E-13	**
ESDzt5	LDzt25	0.0580535	0.0337832	0.08232	4.84E-13	**
SDzt09	ESDzt13	0.0562126	0.0319423	0.08048	4.88E-13	**
ESDzt5	LDzt1	0.0500138	0.028697	0.07133	4.90E-13	**
SDzt09	ESDzt9	0.054226	0.0310191	0.07743	4.91E-13	**
SDzt09	ESDzt15	0.0532724	0.0300656	0.07648	5.02E-13	**
LDzt13	ESDzt11	0.0535152	0.0303084	0.07672	5.11E-13	**
ESDzt5	ELDzt5	0.0488047	0.0274879	0.07012	5.24E-13	**
LDzt13	SDzt15	0.0552927	0.0310224	0.07956	5.27E-13	**
ESDzt5	SDzt21	0.0551887	0.0309184	0.07946	5.42E-13	**
ESDzt5	ESDzt7	0.0484101	0.0270933	0.06973	5.55E-13	**
SDzt09	ELDzt21	0.0525282	0.0293214	0.07574	5.89E-13	**
ESDzt5	LDzt3	0.0482267	0.0269098	0.06954	5.91E-13	**
LDzt13	SDzt17	0.0546518	0.0303814	0.07892	6.11E-13	**
LDzt13	ESDzt9	0.0521042	0.0288973	0.07531	6.15E-13	**
SDzt09	ELDzt19	0.0542227	0.0299524	0.07849	6.88E-13	**
LDzt13	ESDzt13	0.0540908	0.0298205	0.07836	7.34E-13	**
ELDzt15	SDzt13	0.0472734	0.0259565	0.06859	7.93E-13	**
LDzt13	ESDzt15	0.0511506	0.0279438	0.07436	9.31E-13	**
ESDzt5	ESDzt21	0.049511	0.0270411	0.07198	9.40E-13	**
ELDzt15	ESDzt11	0.04687	0.0255531	0.06819	1.01E-12	**
SDzt09	LDzt17	0.0567129	0.0307668	0.08266	1.22E-12	**
LDzt13	ELDzt21	0.0504064	0.0271996	0.07361	1.50E-12	**
ELDzt15	SDzt15	0.0486475	0.0261775	0.07112	1.72E-12	**
SDzt09	ELDzt17	0.0499062	0.0266993	0.07311	2.30E-12	**
LDzt13	ELDzt19	0.0521009	0.0278306	0.07637	2.45E-12	**
ESDzt5	SDzt05	0.0482324	0.0257625	0.0707	2.46E-12	**
ESDzt5	SDzt07	0.0457398	0.0244229	0.06706	2.50E-12	**
ELDzt15	SDzt17	0.0480065	0.0255366	0.07048	3.04E-12	**
ELDzt15	ESDzt9	0.0454589	0.0241421	0.06678	3.32E-12	**
ESDzt5	ESDzt3	0.0476972	0.0252272	0.07017	4.13E-12	**
ELDzt15	ESDzt13	0.0474455	0.0249756	0.06992	5.28E-12	**
LDzt13	LDzt17	0.0545911	0.028645	0.08054	6.29E-12	**
ESDzt5	ELDzt3	0.0448267	0.0235098	0.06614	6.46E-12	**
ELDzt15	ESDzt15	0.0445054	0.0231885	0.06582	9.22E-12	**
LDzt13	ELDzt17	0.0477844	0.0245775	0.07099	1.83E-11	**
ELDzt15	ELDzt21	0.0437612	0.0224443	0.06508	2.13E-11	**
ELDzt15	ELDzt19	0.0454557	0.0229857	0.06793	4.41E-11	**
SDzt09	LDzt19	0.0523798	0.0264338	0.07833	4.88E-11	**
SDzt09	SDzt19	0.0485038	0.0242335	0.07277	8.00E-11	**
SDzt09	LDzt21	0.0462015	0.0229947	0.06941	9.63E-11	**
ELDzt15	LDzt17	0.0479458	0.0236755	0.07222	1.40E-10	**
ESDzt5	SDzt11	0.0476878	0.0234174	0.07196	1.81E-10	**
SDzt09	ELDzt23	0.0475439	0.0232736	0.07181	2.09E-10	**
LDzt13	LDzt19	0.050258	0.024312	0.0762	3.55E-10	**
ELDzt15	ELDzt17	0.0411391	0.0198223	0.06246	4.22E-10	**
LDzt13	SDzt19	0.046382	0.0221117	0.07065	6.63E-10	**
LDzt13	LDzt21	0.0440797	0.0208729	0.06729	8.77E-10	**
LDzt13	ELDzt23	0.0454221	0.0211518	0.06969	1.71E-09	**
SDzt09	LDzt15	0.0480194	0.0220733	0.07397	2.79E-09	**
SDzt09	ESDzt17	0.0420215	0.0188147	0.06523	7.15E-09	**
ELDzt15	LDzt19	0.0436128	0.0193425	0.06788	9.87E-09	**
SDzt09	ESDzt25	0.0412027	0.0179958	0.06441	1.63E-08	**
LDzt13	LDzt15	0.0458976	0.0199515	0.07184	1.89E-08	**
ELDzt15	SDzt19	0.0397368	0.0172669	0.06221	1.91E-08	**
ELDzt15	LDzt21	0.0374345	0.0161176	0.05875	2.54E-08	**
ELDzt3	SDzt13	0.0351197	0.015022	0.05522	3.10E-08	**
SDzt09	LDzt5	0.0404188	0.0172119	0.06363	3.54E-08	**
ELDzt3	ESDzt11	0.0347163	0.0146186	0.05481	4.91E-08	**
ELDzt15	ELDzt23	0.0387769	0.016307	0.06125	5.10E-08	**
LDzt13	ESDzt17	0.0398997	0.0166929	0.06311	5.90E-08	**
ELDzt3	SDzt15	0.0364938	0.015177	0.05781	6.97E-08	**
SDzt09	ELDzt7	0.0413794	0.0171091	0.06565	8.18E-08	**
SDzt07	SDzt13	0.0342067	0.0141089	0.0543	8.74E-08	**
LDzt13	ESDzt25	0.0390809	0.015874	0.06229	1.31E-07	**
ELDzt3	SDzt17	0.0358529	0.014536	0.05717	1.37E-07	**
SDzt07	ESDzt11	0.0338033	0.0137055	0.0539	1.37E-07	**
SDzt07	SDzt15	0.0355808	0.0142639	0.0569	1.83E-07	**
ELDzt3	ESDzt9	0.0333053	0.0132076	0.0534	2.39E-07	**
ELDzt3	ESDzt13	0.0352919	0.013975	0.05661	2.48E-07	**
LDzt13	LDzt5	0.038297	0.0150901	0.0615	2.79E-07	**
SDzt07	SDzt17	0.0349398	0.0136229	0.05626	3.57E-07	**
LDzt13	ELDzt7	0.0392576	0.0149873	0.06353	5.75E-07	**
ELDzt15	LDzt15	0.0392524	0.014982	0.06352	5.78E-07	**
SDzt09	ELDzt9	0.0374721	0.0142653	0.06068	6.11E-07	**
SDzt07	ESDzt13	0.0343788	0.0130619	0.0557	6.38E-07	**
SDzt07	ESDzt9	0.0323922	0.0122945	0.05249	6.52E-07	**
ELDzt3	ESDzt15	0.0323517	0.012254	0.05245	6.82E-07	**
LDzt3	SDzt13	0.0317198	0.0116221	0.05182	1.35E-06	**
SDzt09	LDzt9	0.0365452	0.0133384	0.05975	1.46E-06	**
ELDzt3	ELDzt21	0.0316075	0.0115098	0.05171	1.52E-06	**
ESDzt7	SDzt13	0.0315363	0.0114386	0.05163	1.65E-06	**
SDzt07	ESDzt15	0.0314387	0.0113409	0.05154	1.83E-06	**
ELDzt3	ELDzt19	0.033302	0.0119852	0.05462	1.91E-06	**
ELDzt15	ESDzt17	0.0332545	0.0119376	0.05457	2.00E-06	**
LDzt3	ESDzt11	0.0313164	0.0112187	0.05141	2.08E-06	**
LDzt3	SDzt15	0.0330939	0.011777	0.05441	2.35E-06	**
ELDzt5	SDzt13	0.0311417	0.011044	0.05124	2.51E-06	**
ESDzt7	ESDzt11	0.0311329	0.0110352	0.05123	2.53E-06	**
ESDzt7	SDzt15	0.0329104	0.0115936	0.05423	2.83E-06	**
ELDzt3	LDzt17	0.0357922	0.0125853	0.059	2.92E-06	**
SDzt09	LDzt7	0.0373668	0.0130965	0.06164	3.10E-06	**
ELDzt5	ESDzt11	0.0307383	0.0106406	0.05084	3.84E-06	**
SDzt07	ELDzt21	0.0306944	0.0105967	0.05079	4.02E-06	**
ELDzt5	SDzt15	0.0325158	0.011199	0.05383	4.19E-06	**
LDzt13	ELDzt9	0.0353503	0.0121435	0.05856	4.38E-06	**
LDzt3	SDzt17	0.0324529	0.0111361	0.05377	4.46E-06	**
ELDzt15	ESDzt25	0.0324356	0.0111188	0.05375	4.54E-06	**
SDzt07	ELDzt19	0.032389	0.0110721	0.05371	4.75E-06	**
SDzt09	ESDzt1	0.0352556	0.0120488	0.05846	4.77E-06	**
ESDzt7	SDzt17	0.0322694	0.0109526	0.05359	5.35E-06	**
[illegible]	[illegible]	[illegible]	[illegible]	[illegible]	[illegible]	[illegible]

tableS60_ELDvsELD		diff	lwr	upr	p adj	
ELDzt15	ELDzt21	0.04376117	0.022444316	0.065078024	2.13E-11	**
ELDzt15	ELDzt19	0.045455685	0.022985749	0.067925622	4.41E-11	**
ELDzt15	ELDzt17	0.041139121	0.019822267	0.062455974	4.22E-10	**
ELDzt15	ELDzt23	0.038776901	0.016306964	0.061246838	5.10E-08	**
ELDzt3	ELDzt21	0.031607533	0.011509811	0.051705256	1.52E-06	**
ELDzt3	ELDzt19	0.033302049	0.011985195	0.054618902	1.91E-06	**
ELDzt15	ELDzt7	0.032612348	0.010142411	0.055082285	1.95E-05	**
ELDzt3	ELDzt17	0.028985484	0.008887761	0.049083206	2.35E-05	**
ELDzt5	ELDzt19	0.029324016	0.008007162	0.050640869	8.87E-05	**
ELDzt5	ELDzt21	0.0276295	0.007531778	0.047727223	9.02E-05	**
ELDzt15	ELDzt9	0.028705097	0.007388244	0.050021951	0.000155736	**
ELDzt13	ELDzt19	0.027469572	0.006152719	0.048786426	0.000464941	**
ELDzt13	ELDzt21	0.025775057	0.005677334	0.045872779	0.000520783	**
ELDzt3	ELDzt23	0.026623264	0.005306411	0.047940118	0.00095875	**
ELDzt5	ELDzt17	0.025007451	0.004909728	0.045105173	0.001041836	**
ELDzt15	ELDzt25	0.025687549	0.004370695	0.047004403	0.002079196	**
ELDzt15	ELDzt11	0.026260227	0.00379029	0.048730164	0.00386061	**
ELDzt13	ELDzt17	0.023153007	0.003055285	0.04325073	0.005090222	**
ELDzt15	ELDzt1	0.024114083	0.00279723	0.045430937	0.007141341	**
ELDzt5	ELDzt23	0.022645231	0.001328378	0.043962085	0.020724488	*
ELDzt1	ELDzt19	0.021341602	2.47E-05	0.042658456	0.049225161	*
ELDzt1	ELDzt21	0.019647087	-0.000450636	0.039744809	0.067180739	
ELDzt13	ELDzt23	0.020790788	-0.000526066	0.042107642	0.069160714	
ELDzt3	ELDzt7	0.020458711	-0.000858143	0.041775565	0.084227527	
ELDzt25	ELDzt19	0.019768137	-0.001548717	0.04108499	0.12436327	
ELDzt25	ELDzt21	0.018073621	-0.002024101	0.038171343	0.170270756	
ELDzt11	ELDzt19	0.019195459	-0.003274478	0.041665396	0.268365372	
ELDzt1	ELDzt17	0.017025037	-0.003072685	0.03712276	0.286809912	
ELDzt15	ELDzt13	0.017986113	-0.003330741	0.039302967	0.295730921	
ELDzt3	ELDzt9	0.016551461	-0.003546262	0.036649183	0.35267856	
ELDzt11	ELDzt21	0.017500943	-0.003815911	0.038817797	0.360275883	
ELDzt9	ELDzt19	0.016750588	-0.004566266	0.038067442	0.471962986	
ELDzt5	ELDzt7	0.016480678	-0.004836176	0.037797532	0.514505845	
ELDzt25	ELDzt17	0.015451572	-0.004646151	0.035549294	0.529130712	
ELDzt15	ELDzt5	0.01613167	-0.005185184	0.037448523	0.570286222	
ELDzt9	ELDzt21	0.015056073	-0.00504165	0.035153795	0.596244925	
ELDzt11	ELDzt17	0.014878894	-0.00643796	0.036195748	0.761117391	
ELDzt1	ELDzt23	0.014662818	-0.006654036	0.035979671	0.790020563	
ELDzt13	ELDzt7	0.014626235	-0.006690619	0.035943088	0.794743462	
ELDzt3	ELDzt25	0.013533912	-0.00656381	0.033631635	0.828105575	
ELDzt3	ELDzt11	0.01410659	-0.007210264	0.035423444	0.855886143	
ELDzt5	ELDzt9	0.012573428	-0.007524295	0.03267115	0.924121132	
ELDzt9	ELDzt17	0.012434023	-0.007663699	0.032531746	0.93410259	
ELDzt25	ELDzt23	0.013089352	-0.008227502	0.034406206	0.940245029	
ELDzt3	ELDzt1	0.011960447	-0.008137276	0.032058169	0.961108354	
ELDzt7	ELDzt19	0.012843338	-0.009626599	0.035313275	0.978825637	
ELDzt15	ELDzt3	0.012153637	-0.009163217	0.03347049	0.979655007	
ELDzt11	ELDzt23	0.012516674	-0.009953263	0.034986611	0.986093223	
ELDzt13	ELDzt9	0.010718984	-0.009378738	0.030816707	0.993538228	
ELDzt7	ELDzt21	0.011148822	-0.010168032	0.032465676	0.995529736	
ELDzt5	ELDzt25	0.009555879	-0.010541843	0.029653602	0.999393961	
ELDzt5	ELDzt11	0.010128557	-0.011188297	0.031445411	0.999403388	
ELDzt9	ELDzt23	0.010071804	-0.01124505	0.031388657	0.999475302	
ELDzt7	ELDzt17	0.008526773	-0.012790081	0.029843627	0.999992959	
ELDzt1	ELDzt7	0.008498264	-0.012818589	0.029815118	0.999993605	
ELDzt5	ELDzt1	0.007982414	-0.012115309	0.028080136	0.999994258	
ELDzt13	ELDzt11	0.008274114	-0.01304274	0.029590967	0.999997073	
ELDzt13	ELDzt25	0.007701436	-0.012396287	0.027799158	0.999998005	
ELDzt25	ELDzt7	0.006924799	-0.014392055	0.028241653	0.999999991	
ELDzt13	ELDzt1	0.00612797	-0.013969752	0.026225693	0.999999999	
ELDzt21	ELDzt19	0.001694516	-0.019622338	0.023011369	1	
ELDzt17	ELDzt19	0.004316565	-0.017000289	0.025633419	1	
ELDzt23	ELDzt19	0.006678785	-0.015791152	0.029148721	1	
ELDzt17	ELDzt21	0.002622049	-0.017475673	0.022719772	1	
ELDzt23	ELDzt21	0.004984269	-0.016332585	0.026301123	1	
ELDzt23	ELDzt17	0.00236222	-0.018954634	0.023679073	1	
ELDzt7	ELDzt23	0.006164553	-0.016305384	0.02863449	1	
ELDzt9	ELDzt7	0.00390725	-0.017409603	0.025224104	1	
ELDzt11	ELDzt7	0.006352121	-0.016117816	0.028822058	1	
ELDzt11	ELDzt9	0.002444871	-0.018871983	0.023761724	1	
ELDzt25	ELDzt9	0.003017548	-0.017080174	0.023115271	1	
ELDzt1	ELDzt9	0.004591014	-0.015506708	0.024688736	1	
ELDzt25	ELDzt11	0.000572678	-0.020744176	0.021889532	1	
ELDzt1	ELDzt11	0.002146143	-0.01917071	0.023462997	1	
ELDzt1	ELDzt25	0.001573466	-0.018524257	0.021671188	1	
ELDzt5	ELDzt13	0.001854443	-0.018243279	0.021952166	1	
ELDzt3	ELDzt13	0.005832476	-0.014265246	0.025930199	1	
ELDzt3	ELDzt5	0.003978033	-0.016119689	0.024075755	1	

tableS61_LDvsLD		diff	lwr	upr	p adj	
LDzt13	LDzt17	0.05459105	0.028645002	0.080537098	6.29E-12	**
LDzt13	LDzt19	0.050258046	0.024311998	0.076204094	3.55E-10	**
LDzt13	LDzt21	0.04407974	0.020872889	0.067286591	8.77E-10	**
LDzt13	LDzt15	0.045897594	0.019951546	0.071843642	1.89E-08	**
LDzt13	LDzt5	0.038296971	0.01509012	0.061503822	2.79E-07	**
LDzt13	LDzt9	0.03442344	0.011216589	0.057630291	1.01E-05	**
LDzt13	LDzt7	0.03524504	0.010974735	0.059515346	1.92E-05	**
LDzt3	LDzt17	0.032392202	0.009185352	0.055599053	5.96E-05	**
LDzt13	LDzt23	0.031384491	0.008177641	0.054591342	0.0001393	**
LDzt13	LDzt11	0.031285961	0.00807911	0.054492812	0.00015118	**
LDzt1	LDzt17	0.030605055	0.007398204	0.053811906	0.00026455	**
LDzt13	LDzt25	0.03202567	0.006079622	0.057971718	0.001244462	**
LDzt3	LDzt19	0.028059198	0.004852348	0.051266049	0.001937981	**
LDzt1	LDzt19	0.026272051	0.0030652	0.049478902	0.007043091	**
LDzt3	LDzt21	0.021880893	0.00178317	0.041978615	0.013924281	*
LDzt13	LDzt1	0.023985994	0.000779144	0.047192845	0.031437541	*
LDzt3	LDzt15	0.023698747	0.000491896	0.046905598	0.037412705	*
LDzt11	LDzt17	0.023305089	9.82E-05	0.04651194	0.047225094	*
LDzt23	LDzt17	0.023206558	-2.93E-07	0.046413409	0.050008475	
LDzt1	LDzt21	0.020093745	-3.98E-06	0.040191468	0.050133121	
LDzt13	LDzt3	0.022198847	-0.001008004	0.045405698	0.087617934	
LDzt1	LDzt15	0.0219116	-0.001295251	0.04511845	0.101914674	
LDzt25	LDzt17	0.02256538	-0.003380668	0.048511428	0.231246102	
LDzt9	LDzt17	0.02016761	-0.003039241	0.043374461	0.232775306	
LDzt11	LDzt19	0.018972085	-0.004234766	0.042178936	0.37071011	
LDzt23	LDzt19	0.018873554	-0.004333297	0.042080405	0.383671303	
LDzt3	LDzt5	0.016098124	-0.003999599	0.036195846	0.422274543	
LDzt7	LDzt17	0.019346009	-0.004924296	0.043616315	0.434797602	
LDzt1	LDzt5	0.014310977	-0.005786746	0.034408699	0.718397896	
LDzt25	LDzt19	0.018232376	-0.007713672	0.044178424	0.747119929	
LDzt5	LDzt17	0.016294079	-0.006912772	0.039500929	0.748858284	
LDzt9	LDzt19	0.015834606	-0.007372245	0.039041457	0.805042728	
LDzt11	LDzt21	0.012793779	-0.007303943	0.032891502	0.906328308	
LDzt23	LDzt21	0.012695248	-0.007402474	0.032792971	0.914593806	
LDzt11	LDzt15	0.014611633	-0.008595218	0.037818484	0.91790127	
LDzt23	LDzt15	0.014513103	-0.008693748	0.037719953	0.92447445	
LDzt7	LDzt19	0.015013005	-0.0092573	0.039283311	0.934243782	
LDzt3	LDzt7	0.013046193	-0.008270661	0.034363047	0.942793602	
LDzt3	LDzt9	0.012224592	-0.00787313	0.032322315	0.947313973	
LDzt25	LDzt15	0.013871924	-0.012074124	0.039817972	0.993241655	
LDzt1	LDzt7	0.011259046	-0.010057808	0.0325759	0.994610564	
LDzt25	LDzt21	0.01205407	-0.011152781	0.035260921	0.996086259	
LDzt1	LDzt9	0.010437445	-0.009660277	0.030535168	0.996098535	
LDzt5	LDzt19	0.011961075	-0.011245776	0.035167925	0.996637379	
LDzt9	LDzt15	0.011474154	-0.011732697	0.034681005	0.998566788	
LDzt9	LDzt21	0.0096563	-0.010441422	0.029754023	0.999232941	
LDzt3	LDzt23	0.009185644	-0.010912078	0.029283367	0.999760037	
LDzt21	LDzt17	0.01051131	-0.012695541	0.033718161	0.999807351	
LDzt3	LDzt11	0.009087113	-0.011010609	0.029184836	0.999815455	
LDzt7	LDzt15	0.010652554	-0.013617752	0.034922859	0.999912346	
LDzt3	LDzt25	0.009826823	-0.013380028	0.033033674	0.999965763	
LDzt7	LDzt21	0.008834699	-0.012482154	0.030151553	0.999980903	
LDzt1	LDzt23	0.007398497	-0.012699225	0.027496219	0.999999418	
LDzt1	LDzt11	0.007299966	-0.012797756	0.027397689	0.999999618	
LDzt11	LDzt5	0.00701101	-0.013086712	0.027108733	0.999999896	
LDzt1	LDzt25	0.008039676	-0.015167175	0.031246526	0.999999918	
LDzt23	LDzt5	0.00691248	-0.013185243	0.027010202	0.999999935	
LDzt15	LDzt17	0.008693456	-0.017252592	0.034639504	0.999999973	
LDzt5	LDzt15	0.007600623	-0.015606228	0.030807474	0.999999988	
LDzt19	LDzt17	0.004333004	-0.021613044	0.030279052	1	
LDzt15	LDzt19	0.004360452	-0.021585596	0.0303065	1	
LDzt21	LDzt19	0.006178306	-0.017028545	0.029385157	1	
LDzt21	LDzt15	0.001817854	-0.021388997	0.025024705	1	
LDzt5	LDzt21	0.005782769	-0.014314954	0.025880491	1	
LDzt7	LDzt5	0.003051931	-0.018264923	0.024368784	1	
LDzt9	LDzt5	0.003873531	-0.016224191	0.023971254	1	
LDzt25	LDzt5	0.006271301	-0.01693555	0.029478152	1	
LDzt9	LDzt7	0.000821601	-0.020495253	0.022138454	1	
LDzt25	LDzt7	0.00321937	-0.021050935	0.027489676	1	
LDzt23	LDzt7	0.003860549	-0.017456305	0.025177403	1	
LDzt11	LDzt7	0.00395908	-0.017357774	0.025275933	1	
LDzt25	LDzt9	0.00239777	-0.020809081	0.025604621	1	
LDzt23	LDzt9	0.003038948	-0.017058774	0.023136671	1	
LDzt11	LDzt9	0.003137479	-0.016960243	0.023235201	1	
LDzt23	LDzt25	0.000641179	-0.022565672	0.023848029	1	
LDzt11	LDzt25	0.000739709	-0.022467142	0.02394656	1	
LDzt11	LDzt23	9.85E-05	-0.019999192	0.020196253	1	
LDzt3	LDzt1	0.001787147	-0.018310575	0.02188487	1	

tableS62_SDvsSD		diff	lwr	upr	p adj	
SDzt09	SDzt13	0.056040426	0.032833575	0.079247277	4.67E-13	**
SDzt09	SDzt15	0.057414525	0.03314422	0.081684831	4.69E-13	**
SDzt09	SDzt17	0.056773556	0.03250325	0.081043862	4.83E-13	**
SDzt09	SDzt19	0.048503831	0.024233526	0.072774137	8.00E-11	**
SDzt07	SDzt13	0.034206659	0.014108936	0.054304381	8.74E-08	**
SDzt07	SDzt15	0.035580758	0.014263904	0.056897612	1.83E-07	**
SDzt07	SDzt17	0.034939789	0.013622935	0.056256643	3.57E-07	**
SDzt05	SDzt13	0.031713995	0.010397142	0.053030849	9.22E-06	**
SDzt09	SDzt25	0.036101152	0.011830846	0.060371457	9.27E-06	**
SDzt05	SDzt15	0.033088095	0.010618158	0.055558032	1.26E-05	**
SDzt05	SDzt17	0.032447125	0.009977189	0.054917062	2.26E-05	**
SDzt09	SDzt23	0.034042192	0.009771886	0.058312497	5.20E-05	**
SDzt11	SDzt13	0.032258682	0.009051831	0.055465533	6.68E-05	**
SDzt11	SDzt15	0.033632781	0.009362475	0.057903087	7.27E-05	**
SDzt11	SDzt17	0.032991812	0.008721506	0.057262117	0.000121734	**
SDzt09	SDzt01	0.031364191	0.00815734	0.054571042	0.000141671	**
SDzt07	SDzt19	0.026670064	0.00535321	0.047986918	0.000921663	**
SDzt09	SDzt03	0.030139693	0.005869387	0.054409998	0.001088633	**
SDzt01	SDzt13	0.024676235	0.004578513	0.044773958	0.001396304	**
SDzt01	SDzt15	0.026050334	0.004733481	0.047367188	0.001545316	**
SDzt03	SDzt13	0.025900733	0.00458388	0.047217587	0.001747398	**
SDzt03	SDzt15	0.027274833	0.004804896	0.04974477	0.001784339	**
SDzt09	SDzt21	0.031282664	0.005336616	0.057228712	0.002056052	**
SDzt01	SDzt17	0.025409365	0.004092511	0.046726219	0.002602666	**
SDzt03	SDzt17	0.026633863	0.004163927	0.0491038	0.002916392	**
SDzt21	SDzt15	0.026131861	0.001861556	0.050402167	0.016716614	*
SDzt05	SDzt19	0.024177401	0.001707464	0.046647338	0.016897053	*
SDzt21	SDzt13	0.024757762	0.001550911	0.047964613	0.019381569	*
SDzt21	SDzt17	0.025490892	0.001220586	0.049761198	0.024723223	*
SDzt23	SDzt15	0.023372334	0.000902397	0.04584227	0.028618543	*
SDzt23	SDzt13	0.021998234	0.000681381	0.043315088	0.032163043	*
SDzt11	SDzt19	0.024722087	0.000451781	0.048992393	0.038778901	*
SDzt23	SDzt17	0.022731364	0.000261427	0.045201301	0.042695831	*
SDzt09	SDzt05	0.024326431	5.61E-05	0.048596736	0.048467002	*
SDzt25	SDzt15	0.021313374	-0.001156563	0.04378331	0.096696223	
SDzt09	SDzt07	0.021833767	-0.001373084	0.045040618	0.106101435	
SDzt25	SDzt13	0.019939274	-0.001377579	0.041256128	0.113211106	
SDzt25	SDzt17	0.020672404	-0.001797533	0.043142341	0.135314868	
SDzt09	SDzt11	0.023781744	-0.002164304	0.049727792	0.140663068	
SDzt03	SDzt19	0.018364139	-0.004105798	0.040834076	0.371452082	
SDzt01	SDzt19	0.01713964	-0.004177213	0.038456494	0.412547052	
SDzt21	SDzt19	0.017221167	-0.007049138	0.041491473	0.726215559	
SDzt07	SDzt25	0.014267385	-0.007049469	0.035584238	0.838205047	
SDzt23	SDzt19	0.014461639	-0.008008297	0.036931576	0.89360938	
SDzt07	SDzt23	0.012208425	-0.009108429	0.033525278	0.978152626	
SDzt25	SDzt19	0.012402679	-0.010067257	0.034872616	0.988094265	
SDzt05	SDzt25	0.011774721	-0.010695216	0.034244658	0.995360402	
SDzt11	SDzt25	0.012319407	-0.011950898	0.036589713	0.997524321	
SDzt07	SDzt01	0.009530424	-0.010567299	0.029628146	0.99942969	
SDzt05	SDzt23	0.009715761	-0.012754176	0.032185698	0.999940475	
SDzt11	SDzt23	0.010260447	-0.014009858	0.034530753	0.999967225	
SDzt07	SDzt21	0.009448897	-0.013757954	0.032655748	0.999988344	
SDzt19	SDzt15	0.008910694	-0.013559243	0.031380631	0.999994512	
SDzt07	SDzt03	0.008305925	-0.013010928	0.029622779	0.999996721	
SDzt19	SDzt17	0.008269725	-0.014200212	0.030739662	0.999999422	
SDzt19	SDzt13	0.007536595	-0.013780259	0.028853448	0.999999839	
SDzt05	SDzt01	0.00703776	-0.014279093	0.028354614	0.999999984	
SDzt11	SDzt01	0.007582446	-0.015624404	0.030789297	0.999999989	
SDzt17	SDzt15	0.000640969	-0.021828968	0.023110906	1	
SDzt13	SDzt15	0.001374099	-0.019942754	0.022690953	1	
SDzt13	SDzt17	0.00073313	-0.020583724	0.022049984	1	
SDzt23	SDzt25	0.00205896	-0.020410977	0.024528897	1	
SDzt01	SDzt25	0.004736961	-0.016579893	0.026053815	1	
SDzt21	SDzt25	0.004818488	-0.019451818	0.029088793	1	
SDzt03	SDzt25	0.005961459	-0.016508478	0.028431396	1	
SDzt01	SDzt23	0.002678001	-0.018638853	0.023994855	1	
SDzt21	SDzt23	0.002759528	-0.021510778	0.027029833	1	
SDzt03	SDzt23	0.003902499	-0.018567438	0.026372436	1	
SDzt21	SDzt01	8.15E-05	-0.023125324	0.023288378	1	
SDzt03	SDzt01	0.001224498	-0.020092355	0.022541352	1	
SDzt03	SDzt21	0.001142972	-0.023127334	0.025413277	1	
SDzt05	SDzt21	0.006956234	-0.017314072	0.031226539	1	
SDzt11	SDzt21	0.00750092	-0.018445128	0.033446968	1	
SDzt05	SDzt03	0.005813262	-0.016656675	0.028283199	1	
SDzt11	SDzt03	0.006357948	-0.017912357	0.030628254	1	
SDzt11	SDzt05	0.000544686	-0.023725619	0.024814992	1	
SDzt07	SDzt05	0.002492663	-0.01882419	0.023809517	1	
SDzt07	SDzt11	0.001947977	-0.021258874	0.025154828	1	

tableS63_ESDvsESD		diff	lwr	upr	p adj	
ESDzt5	ESDzt13	0.080118571	0.057648634	0.102588508	0	**
ESDzt5	ESDzt11	0.079543038	0.058226184	0.100859891	0	**
ESDzt5	ESDzt9	0.078131985	0.056815131	0.099448838	0	**
ESDzt5	ESDzt15	0.077178435	0.055861581	0.098495289	0	**
ESDzt5	ESDzt17	0.065927538	0.044610684	0.087244391	0	**
ESDzt5	ESDzt25	0.065108693	0.04379184	0.086425547	0	**
ESDzt5	ESDzt1	0.059161615	0.037844761	0.080478468	1.57E-13	**
ESDzt5	ESDzt23	0.058557756	0.037240903	0.07987461	1.94E-13	**
ESDzt5	ESDzt19	0.054295957	0.032979103	0.07561281	4.32E-13	**
ESDzt5	ESDzt7	0.048410117	0.027093263	0.06972697	5.55E-13	**
ESDzt5	ESDzt21	0.04951099	0.027041053	0.071980927	9.40E-13	**
ESDzt5	ESDzt3	0.047697161	0.025227224	0.070167098	4.13E-12	**
ESDzt7	ESDzt11	0.031132921	0.011035198	0.051230643	2.53E-06	**
ESDzt3	ESDzt11	0.031845876	0.010529023	0.05316273	8.10E-06	**
ESDzt7	ESDzt13	0.031708454	0.0103916	0.053025308	9.27E-06	**
ESDzt7	ESDzt9	0.029721868	0.009624145	0.04981959	1.11E-05	**
ESDzt3	ESDzt13	0.032421409	0.009951473	0.054891346	2.32E-05	**
ESDzt7	ESDzt15	0.028768318	0.008670596	0.048866041	2.92E-05	**
ESDzt3	ESDzt9	0.030434823	0.00911797	0.051751677	3.15E-05	**
ESDzt21	ESDzt11	0.030032048	0.008715194	0.051348901	4.60E-05	**
ESDzt3	ESDzt15	0.029481274	0.00816442	0.050798128	7.67E-05	**
ESDzt21	ESDzt13	0.030607581	0.008137644	0.053077518	0.000115279	**
ESDzt21	ESDzt9	0.028620995	0.007304141	0.049937848	0.000167997	**
ESDzt21	ESDzt15	0.027667445	0.006350592	0.048984299	0.000391362	**
ESDzt19	ESDzt11	0.025247081	0.005149358	0.045344803	0.000840879	**
ESDzt19	ESDzt13	0.025822614	0.00450576	0.047139468	0.00186267	**
ESDzt19	ESDzt9	0.023836028	0.003738305	0.04393375	0.002882105	**
ESDzt19	ESDzt15	0.022882478	0.002784756	0.042980201	0.006342582	**
ESDzt23	ESDzt11	0.020985281	0.000887559	0.041083004	0.027018396	*
ESDzt1	ESDzt11	0.020381423	0.0002837	0.040479145	0.041265129	*
ESDzt23	ESDzt13	0.021560814	0.000243961	0.042877668	0.042807177	*
ESDzt1	ESDzt13	0.020956956	-0.000359898	0.04227381	0.062523796	
ESDzt23	ESDzt9	0.019574228	-0.000523494	0.039671951	0.070386187	
ESDzt1	ESDzt9	0.01897037	-0.001127353	0.039068092	0.102259256	
ESDzt23	ESDzt15	0.018620679	-0.001477044	0.038718401	0.125580202	
ESDzt1	ESDzt15	0.01801682	-0.002080902	0.038114543	0.175523449	
ESDzt7	ESDzt17	0.017517421	-0.002580302	0.037615143	0.226908797	
ESDzt3	ESDzt17	0.018230376	-0.003086477	0.03954723	0.266009325	
ESDzt7	ESDzt25	0.016698577	-0.003399146	0.036796299	0.331403109	
ESDzt3	ESDzt25	0.017411532	-0.003905322	0.038728386	0.372906451	
ESDzt21	ESDzt17	0.016416548	-0.004900306	0.037733401	0.524715488	
ESDzt21	ESDzt25	0.015597704	-0.00571915	0.036914557	0.654952044	
ESDzt25	ESDzt11	0.014434344	-0.005663378	0.034532067	0.698982419	
ESDzt25	ESDzt13	0.015009877	-0.006306976	0.036326731	0.74281591	
ESDzt17	ESDzt11	0.0136155	-0.006482222	0.033713222	0.817794894	
ESDzt17	ESDzt13	0.014191033	-0.00712582	0.035507887	0.84674439	
ESDzt25	ESDzt9	0.013023291	-0.007074431	0.033121014	0.885108542	
ESDzt17	ESDzt9	0.012204447	-0.007893275	0.032302169	0.948475702	
ESDzt25	ESDzt15	0.012069742	-0.008027981	0.032167464	0.955771678	
ESDzt19	ESDzt17	0.011631581	-0.008466142	0.031729303	0.97428039	
ESDzt17	ESDzt15	0.011250898	-0.008846825	0.03134862	0.984891821	
ESDzt19	ESDzt25	0.010812737	-0.009284986	0.030910459	0.992422597	
ESDzt3	ESDzt1	0.011464453	-0.0098524	0.032781307	0.992472583	
ESDzt7	ESDzt1	0.010751498	-0.009346224	0.03084922	0.993167964	
ESDzt3	ESDzt23	0.010860595	-0.010456259	0.032177449	0.997330611	
ESDzt7	ESDzt23	0.01014764	-0.009950083	0.030245362	0.997777646	
ESDzt21	ESDzt1	0.009650625	-0.011666229	0.030967479	0.9998096	
ESDzt21	ESDzt23	0.009046767	-0.012270087	0.03036362	0.999963646	
ESDzt23	ESDzt17	0.007369781	-0.012727941	0.027467504	0.999999485	
ESDzt1	ESDzt17	0.006765923	-0.0133318	0.026863645	0.999999968	
ESDzt23	ESDzt25	0.006550937	-0.013546785	0.026648659	0.99999999	
ESDzt3	ESDzt19	0.006598795	-0.014718058	0.027915649	0.999999998	
ESDzt11	ESDzt13	0.000575533	-0.02074132	0.021892387	1	
ESDzt9	ESDzt13	0.001986586	-0.019330267	0.02330344	1	
ESDzt15	ESDzt13	0.002940136	-0.018376718	0.024256989	1	
ESDzt9	ESDzt11	0.001411053	-0.018686669	0.021508775	1	
ESDzt15	ESDzt11	0.002364602	-0.01773312	0.022462325	1	
ESDzt15	ESDzt9	0.000953549	-0.019144173	0.021051272	1	
ESDzt25	ESDzt17	0.000818844	-0.019278878	0.020916567	1	
ESDzt1	ESDzt25	0.005947079	-0.014150644	0.026044801	1	
ESDzt23	ESDzt1	0.000603858	-0.019493864	0.020701581	1	
ESDzt19	ESDzt1	0.004865658	-0.015232064	0.02496338	1	
ESDzt19	ESDzt23	0.0042618	-0.015835923	0.024359522	1	
ESDzt21	ESDzt19	0.004784967	-0.016531887	0.026101821	1	
ESDzt7	ESDzt19	0.00588584	-0.014211882	0.025983562	1	
ESDzt7	ESDzt21	0.001100873	-0.020215981	0.022417727	1	
ESDzt3	ESDzt21	0.001813829	-0.020656108	0.024283765	1	
ESDzt3	ESDzt7	0.000712955	-0.020603898	0.022029809	1	

Table S64 Two-way ANOVA for data shown in fig7a#,$

ZT vs Light condition			Light condition			ZT		
Degree of freedom	F value	*p* value	Degree of freedom	F value	*p* value	Degree of freedom	F value	*p* value
(8, 46)	4.77	0.000261	(1, 46)	28.61	2.71E-06	(8, 46)	16.36	3.88E-11

#, Result of post hoc analysis is in tableS65.

$, Result of post hoc analysis for each light condition is in "Okano_tableS66-S67.

tableS65_fig7a_posthoc

	diff	lwr	upr	p adj	
Light13 vs Light16	0.065261	0.038957964	0.091564036	9.03E-10	**
Light13 vs Dark15	0.0647725	0.038469464	0.091075536	1.13E-09	**
Light13 vs Dark16	0.066766417	0.038355891	0.095176942	4.45E-09	**
Light13 vs Dark11	0.05771375	0.031410714	0.084016786	3.08E-08	**
Light13 vs Dark10	0.0542795	0.027976464	0.080582536	1.56E-07	**
Light13 vs Light15	0.0498145	0.023511464	0.076117536	1.32E-06	**
Light13 vs Light11	0.04719175	0.020888714	0.073494786	4.66E-06	**
Light13 vs Dark12	0.04702225	0.020719214	0.073325286	5.05E-06	**
Light14 vs Light16	0.050710583	0.022300058	0.079121109	5.23E-06	**
Light14 vs Dark15	0.050222083	0.021811558	0.078632609	6.50E-06	**
Light13 vs Light10	0.046418	0.020114964	0.072721036	6.75E-06	**
Light13 vs Light05	0.049137417	0.020726891	0.077547942	1.05E-05	**
Light14 vs Dark16	0.052216	0.02184387	0.08258813	1.20E-05	**
Light13 vs Dark05	0.04793775	0.019527224	0.076348276	1.79E-05	**
Light13 vs Dark13	0.04323825	0.016935214	0.069541286	3.08E-05	**
Light13 vs Light14	0.05057375	0.018359242	0.082788258	7.74E-05	**
Dark01 vs Light16	0.043597917	0.015187391	0.072008442	0.000119846	**
Light14 vs Dark11	0.043163333	0.014752808	0.071573859	0.000144709	**
Dark01 vs Dark15	0.043109417	0.014698891	0.071519942	0.000148128	**
Dark01 vs Dark16	0.045103333	0.014731204	0.075475463	0.000220299	**
Light14 vs Dark10	0.039729083	0.011318558	0.068139609	0.000629336	**
Light01 vs Light16	0.0354875	0.009184464	0.061790536	0.001130656	**
Light01 vs Dark15	0.034999	0.008695964	0.061302036	0.001407161	**
Light01 vs Dark16	0.036992917	0.008582391	0.065403442	0.001963681	**
Light12 vs Light16	0.036432583	0.008022058	0.064843109	0.002467441	**
Dark01 vs Dark11	0.036050667	0.007640141	0.064461192	0.00288001	**
Light12 vs Dark15	0.035944083	0.007533558	0.064354609	0.003006531	**
Light12 vs Dark16	0.037938	0.00756587	0.06831013	0.003611305	**
Light14 vs Light15	0.035264083	0.006853558	0.063674609	0.003948836	**
Light14 vs Light11	0.032641333	0.004230808	0.061051859	0.010964989	*
Dark01 vs Dark10	0.032616417	0.004205891	0.061026942	0.011069048	*
Light14 vs Dark12	0.032471833	0.004061308	0.060882359	0.01169144	*
Light14 vs Light05	0.034587	0.00421487	0.06495913	0.012226644	*
Light13 vs Light01	0.0297735	0.003470464	0.056076536	0.013152081	*
Light14 vs Light10	0.031867583	0.003457058	0.060278109	0.014665591	*
Light14 vs Dark05	0.033387333	0.003015204	0.063759463	0.01855387	*
Light01 vs Dark11	0.02794025	0.001637214	0.054243286	0.027129663	*
Light14 vs Dark14	0.036023333	0.00206626	0.069980406	0.027514638	*
Light12 vs Dark11	0.028885333	0.000474808	0.057295859	0.04258352	*
Light13 vs Light12	0.028828417	0.000417891	0.057238942	0.043417338	*
Light14 vs Dark13	0.028687833	0.000277308	0.057098359	0.04553984	*
Dark01 vs Light15	0.028151417	-0.000259109	0.056561942	0.054513889	
Light01 vs Dark10	0.024506	-0.001797036	0.050809036	0.093469319	
Dark01 vs Light05	0.027474333	-0.002897796	0.057846463	0.117998616	
Dark01 vs Light11	0.025528667	-0.002881859	0.053939192	0.124121334	
Light12 vs Dark10	0.025451083	-0.002959442	0.053861609	0.126979608	
Dark01 vs Dark12	0.025359167	-0.003051359	0.053769692	0.130434759	
Dark01 vs Light10	0.024754917	-0.003655609	0.053165442	0.155069561	
Dark01 vs Dark05	0.026274667	-0.004097463	0.056646796	0.162948873	
Dark01 vs Dark14	0.028910667	-0.005046406	0.06286774	0.181341413	
Dark13 vs Light16	0.02202275	-0.004280286	0.048325786	0.201889644	
Dark13 vs Dark16	0.023528167	-0.004882359	0.051938692	0.216044363	
Dark13 vs Dark15	0.02153425	-0.004768786	0.047837286	0.231449918	
Dark13 vs Dark01	0.021663083	-0.006747442	0.050073609	0.338404087	
Light01 vs Light15	0.020041	-0.006262036	0.046344036	0.339628332	
Dark01 vs Dark13	0.021575167	-0.006835359	0.049985692	0.345024445	
Light12 vs Light15	0.020986083	-0.007424442	0.049396609	0.391141418	
Light10 vs Light16	0.018843	-0.007460036	0.045146036	0.443746177	
Light10 vs Dark16	0.020348417	-0.008062109	0.048758942	0.444116758	
Light10 vs Dark15	0.0183545	-0.007948536	0.044657536	0.489453768	
Light12 vs Dark16	0.019744167	-0.008666359	0.048154692	0.496583174	
Dark12 vs Light16	0.01823875	-0.008064286	0.044541786	0.50047433	
Light11 vs Dark16	0.019574667	-0.008835859	0.047985192	0.511588563	
Light11 vs Light16	0.01806925	-0.008233786	0.044372286	0.516713416	
Light01 vs Light05	0.019363917	-0.009046609	0.047774442	0.530369343	
Dark12 vs Dark15	0.01775025	-0.008552786	0.044053286	0.547518145	
Light12 vs Light05	0.020309	-0.01006313	0.05068113	0.56325024	
Light11 vs Dark15	0.01758075	-0.008722286	0.043883786	0.563964973	
Light01 vs Light11	0.01741825	-0.008884786	0.043721286	0.579750245	
Light01 vs Dark12	0.01724875	-0.009054286	0.043551786	0.596205921	
Light12 vs Light11	0.018363333	-0.010047192	0.046773859	0.620166109	
Light01 vs Dark14	0.02080025	-0.011414258	0.053014758	0.621883832	
Light12 vs Dark12	0.018193833	-0.010216692	0.046604359	0.635265576	
Light12 vs Dark14	0.021745333	-0.01221174	0.055702406	0.635299522	
Light01 vs Dark05	0.01816425	-0.010246276	0.046574776	0.637891828	
Light01 vs Light10	0.0166445	-0.009658536	0.042947536	0.654340988	
Light12 vs Dark05	0.019109333	-0.011262796	0.049481463	0.663377215	
Dark05 vs Light16	0.018828667	-0.011543463	0.049200796	0.686167985	
Light12 vs Light10	0.017589583	-0.010820942	0.046000109	0.688147322	
Dark05 vs Light16	0.01732325	-0.011087276	0.045733776	0.710786838	
Light15 vs Dark16	0.016951917	-0.011458609	0.045362442	0.741431109	
Dark05 vs Dark15	0.01683475	-0.011575776	0.045245276	0.750844374	
Light15 vs Light16	0.0154465	-0.010856536	0.041749536	0.762746208	
Light05 vs Dark16	0.017629	-0.01274313	0.04800113	0.777701272	
Light15 vs Dark15	0.014958	-0.011345036	0.041261036	0.802476451	
Light05 vs Light16	0.016123583	-0.012286942	0.044534109	0.804844563	
Dark13 vs Dark11	0.0144755	-0.011827536	0.040778536	0.838328097	
Light05 vs Dark15	0.015635083	-0.012775442	0.044045609	0.838344002	
Light14 vs Light01	0.015223083	-0.013187442	0.043633609	0.864028205	
Light13 vs Light14	0.014550417	-0.013860109	0.042960942	0.900561558	
Light01 vs Dark13	0.01346475	-0.012838286	0.039767786	0.900897063	
Light12 vs Dark13	0.014409833	-0.014000692	0.042820359	0.907329736	
Dark14 vs Dark16	0.016192667	-0.017764406	0.05014974	0.942341073	
Light14 vs Light12	0.014278	-0.01609413	0.04465013	0.948665915	
Dark14 vs Light16	0.01468725	-0.017527258	0.046901758	0.960319493	
Dark14 vs Dark15	0.01419875	-0.018015758	0.046413258	0.970511474	
Dark10 vs Dark16	0.012486917	-0.015923609	0.040897442	0.971247891	
Light10 vs Dark11	0.01129575	-0.015007286	0.037598786	0.976730776	
Dark13 vs Dark10	0.01104125	-0.015261786	0.037344286	0.981209239	
Dark10 vs Light16	0.0109815	-0.015321536	0.037284536	0.982156584	
Dark12 vs Dark11	0.0106915	-0.015611536	0.036994536	0.986238259	
Light11 vs Dark11	0.010522	-0.015781036	0.036825036	0.988257663	
Dark10 vs Dark15	0.010493	-0.015810036	0.036796036	0.988578178	
Dark05 vs Dark11	0.009776	-0.018634526	0.038186526	0.997675549	
Dark11 vs Dark16	0.009052667	-0.019357859	0.037463192	0.999060571	
Light05 vs Dark11	0.008576333	-0.019834192	0.036986859	0.999517224	
Light15 vs Dark11	0.00789925	-0.018403786	0.034202286	0.999547497	
Light10 vs Dark10	0.0078615	-0.018441536	0.034164536	0.99957396	
Dark11 vs Light16	0.00754725	-0.018755786	0.033850286	0.999746688	
Dark01 vs Light01	0.008110417	-0.020300109	0.036520942	0.999762855	
Dark12 vs Dark10	0.00725725	-0.019045786	0.033560286	0.999847935	
Light11 vs Dark10	0.00708775	-0.019215286	0.033390786	0.999888773	
Dark11 vs Dark15	0.00705875	-0.019244286	0.033361786	0.999894686	
Dark13 vs Light15	0.00657625	-0.019726786	0.032879286	0.999959658	
Dark01 vs Light13	0.007165333	-0.023206796	0.037537463	0.999982021	
Light14 vs Dark01	0.007112667	-0.023259463	0.037484796	0.999983797	
Dark13 vs Dark14	0.0073355	-0.024879008	0.039550008	0.999989116	
Dark05 vs Dark10	0.00634175	-0.022068776	0.034752276	0.999991814	
Dark14 vs Dark11	0.00714	-0.025074508	0.039354508	0.999992608	
Dark13 vs Light05	0.005899167	-0.022511359	0.034309692	0.999997137	
Light05 vs Dark10	0.005142083	-0.023268442	0.033552609	0.999999633	
Light15 vs Dark10	0.004465	-0.021838036	0.030768036	0.999999862	
Dark13 vs Dark05	0.0046995	-0.023711026	0.033110026	0.999999908	
Dark13 vs Light11	0.0039535	-0.022349536	0.030256536	0.999999979	
Dark13 vs Dark12	0.003784	-0.022519036	0.030087036	0.99999999	
Dark10 vs Dark11	0.00343425	-0.022868786	0.029737286	0.999999998	
Light10 vs Light15	0.0033965	-0.022906536	0.029699536	0.999999998	
Light10 vs Dark14	0.00415575	-0.028058758	0.036370258	0.999999998	
Dark13 vs Light10	0.00317975	-0.023123286	0.029482786	0.999999999	
Light14 vs Dark10	0.00370575	-0.028508758	0.035920258	1	
Dark12 vs Dark14	0.0035515	-0.028663008	0.035766008	1	
Dark12 vs Light15	0.00279225	-0.023510786	0.029095286	1	
Light11 vs Dark14	0.003382	-0.028832508	0.035596508	1	
Light11 vs Light15	0.00262275	-0.023680286	0.028925786	1	
Light10 vs Light05	0.002719417	-0.025691109	0.031129942	1	
Light16 vs Dark16	0.001505417	-0.026905109	0.029915942	1	
Dark15 vs Dark16	0.001993917	-0.026416609	0.030404442	1	
Dark15 vs Light16	0.0004885	-0.025814536	0.026791536	1	
Light15 vs Dark14	0.00075925	-0.031455258	0.032973758	1	
Light05 vs Dark14	0.001436333	-0.03252074	0.035393406	1	
Dark05 vs Dark14	0.002636	-0.031321073	0.036593073	1	
Light05 vs Light15	0.000677083	-0.027733442	0.029087609	1	
Dark05 vs Light15	0.00187675	-0.026533776	0.030287276	1	
Dark05 vs Light05	0.001199667	-0.029172463	0.031571796	1	
Light11 vs Light05	0.001945667	-0.026464859	0.030356192	1	
Dark12 vs Light05	0.002115167	-0.026295359	0.030525692	1	
Light11 vs Dark05	0.000746	-0.027664526	0.029156526	1	
Dark12 vs Dark05	0.0009155	-0.027495026	0.029326026	1	
Light10 vs Dark05	0.00151975	-0.026890776	0.029930276	1	
Dark12 vs Light11	0.0001695	-0.026133536	0.026472536	1	
Light10 vs Light11	0.00077375	-0.025529286	0.027076786	1	
Light10 vs Dark12	0.00060425	-0.025698786	0.026907286	1	
Light12 vs Light01	0.000945083	-0.027465442	0.029355609	1	

tableS66_Light_vs_Light

	diff	lwr	upr	p adj	
Light13 vs Light16	0.065261	0.038957964	0.091564036	9.03E-10	**
Light13 vs Light15	0.0498145	0.023511464	0.076117536	0.00000132	**
Light13 vs Light11	0.04719175	0.020888714	0.073494786	0.00000466	**
Light14 vs Light16	0.050710583	0.022300058	0.079121109	0.00000523	**
Light13 vs Light10	0.046418	0.020114964	0.072721036	0.00000675	**
Light13 vs Light05	0.049137417	0.020726891	0.077547942	0.0000105	**
Light01 vs Light16	0.0354875	0.009184464	0.061790536	0.001130656	**
Light12 vs Light16	0.036432583	0.008022058	0.064843109	0.002467441	**
Light14 vs Light15	0.035264083	0.006853558	0.063674609	0.003948836	**
Light14 vs Light11	0.032641333	0.004230808	0.061051859	0.010964989	*
Light14 vs Light05	0.034587	0.00421487	0.06495913	0.012226644	*
Light13 vs Light01	0.0297735	0.003470464	0.056076536	0.013152081	*
Light14 vs Light10	0.031867583	0.003457058	0.060278109	0.014665591	*
Light13 vs Light12	0.028828417	0.000417891	0.057238942	0.043417338	*
Light01 vs Light15	0.020041	-0.006262036	0.046344036	0.339628332	
Light12 vs Light15	0.020986083	-0.007424442	0.049396609	0.391141418	
Light10 vs Light16	0.018843	-0.007460036	0.045146036	0.443746177	
Light11 vs Light16	0.01806925	-0.008233786	0.044372286	0.516713416	
Light01 vs Light05	0.019363917	-0.009046609	0.047774442	0.530369343	
Light12 vs Light05	0.020309	-0.01006313	0.05068113	0.56325024	
Light01 vs Light11	0.01741825	-0.008884786	0.043721286	0.579750245	
Light12 vs Light11	0.018363333	-0.010047192	0.046773859	0.620166109	
Light01 vs Light10	0.0166445	-0.009658536	0.042947536	0.654340988	
Light12 vs Light10	0.017589583	-0.010820942	0.046000109	0.688147322	
Light15 vs Light16	0.0154465	-0.010856536	0.041749536	0.762746208	
Light05 vs Light16	0.016123583	-0.012286942	0.044534109	0.804844563	
Light14 vs Light01	0.015223083	-0.013187442	0.043633609	0.864028205	
Light13 vs Light14	0.014550417	-0.013860109	0.042960942	0.900561558	
Light14 vs Light12	0.014278	-0.01609413	0.04465013	0.948665915	
Light10 vs Light15	0.0033965	-0.022906536	0.029699536	0.999999998	
Light11 vs Light15	0.00262275	-0.023680286	0.028925786	1	
Light10 vs Light05	0.002719417	-0.025691109	0.031129942	1	
Light05 vs Light15	0.000677083	-0.027733442	0.029087609	1	
Light11 vs Light05	0.001945667	-0.026464859	0.030356192	1	
Light10 vs Light11	0.00077375	-0.025529286	0.027076786	1	
Light12 vs Light01	0.000945083	-0.027465442	0.029355609	1	

tableS67_Dark_vs_Dark

	diff	lwr	upr	p adj	
Dark01 vs Dark15	0.043109417	0.014698891	0.071519942	0.000148128	**
Dark01 vs Dark16	0.045103333	0.014731204	0.075475463	0.000220299	**
Dark01 vs Dark11	0.036050667	0.007640141	0.064461192	0.00288001	**
Dark01 vs Dark10	0.032616417	0.004205891	0.061026942	0.011069048	*
Dark01 vs Dark12	0.025359167	-0.003051359	0.053769692	0.130434759	
Dark01 vs Dark05	0.026274667	-0.004097463	0.056646796	0.162948873	
Dark01 vs Dark14	0.028910667	-0.005046406	0.06286774	0.181341413	
Dark13 vs Dark16	0.023528167	-0.004882359	0.051938692	0.216044363	
Dark13 vs Dark15	0.02153425	-0.004768786	0.047837286	0.231449918	
Dark01 vs Dark13	0.021575167	-0.006835359	0.049985692	0.345024445	
Dark12 vs Dark16	0.019744167	-0.008666359	0.048154692	0.496583174	
Dark12 vs Dark15	0.01775025	-0.008552786	0.044053286	0.547518145	
Dark05 vs Dark16	0.018828667	-0.011543463	0.049200796	0.686167985	
Dark05 vs Dark15	0.01683475	-0.011575776	0.045245276	0.750844374	
Dark13 vs Dark11	0.0144755	-0.011827536	0.040778536	0.838328097	
Dark14 vs Dark16	0.016192667	-0.017764406	0.05014974	0.942341073	
Dark14 vs Dark15	0.01419875	-0.018015758	0.046413258	0.970511474	
Dark10 vs Dark16	0.012486917	-0.015923609	0.040897442	0.971247891	
Dark13 vs Dark10	0.01104125	-0.015261786	0.037344286	0.981209239	
Dark12 vs Dark11	0.0106915	-0.015611536	0.036994536	0.986238259	
Dark10 vs Dark15	0.010493	-0.015810036	0.036796036	0.988578178	
Dark05 vs Dark11	0.009776	-0.018634526	0.038186526	0.997675549	
Dark11 vs Dark16	0.009052667	-0.019357859	0.037463192	0.999060571	
Dark12 vs Dark10	0.00725725	-0.019045786	0.033560286	0.999847935	
Dark11 vs Dark15	0.00705875	-0.019244286	0.033361786	0.999894686	
Dark13 vs Dark14	0.0073355	-0.024879008	0.039550008	0.999989116	
Dark05 vs Dark10	0.00634175	-0.022068776	0.034752276	0.999991814	
Dark14 vs Dark11	0.00714	-0.025074508	0.039354508	0.999992608	
Dark13 vs Dark05	0.0046995	-0.023711026	0.033110026	0.999999908	
Dark13 vs Dark12	0.003784	-0.022519036	0.030087036	0.99999999	
Dark10 vs Dark11	0.00343425	-0.022868786	0.029737286	0.999999998	
Dark14 vs Dark10	0.00370575	-0.028508758	0.035920258	1	
Dark12 vs Dark14	0.0035515	-0.028663008	0.035766008	1	
Dark15 vs Dark16	0.001993917	-0.026416609	0.030404442	1	
Dark05 vs Dark14	0.002636	-0.031321073	0.036593073	1	
Dark12 vs Dark05	0.0009155	-0.027495026	0.029326026	1	

Table S68 Two-way ANOVA for data shown in fig7b#,$

ZT vs Light condition			Light condition			ZT		
Degree of freedom	F value	*p* value	Degree of freedom	F value	*p* value	Degree of freedom	F value	*p* value
(9, 54)	2.354	0.0253	(1, 54)	3.276	7.59E-02	(9, 54)	11.61	6.83E-10

#Result of post hoc analysis is in tableS69.
$Result of post hoc analysis for each light conditionis is in tableS70-S71.

tableS69_fig7b_posthoc

	diff	lwr	upr	p adj	
Light10 vs Light16	0.036072896	0.017158283	0.054987508	5.04E-07	**
Light11 vs Light16	0.034648767	0.015734155	0.05356338	1.42E-06	**
Light10 vs Dark16	0.034705376	0.01427526	0.055135493	8.85E-06	**
Light11 vs Dark16	0.033281248	0.012851131	0.053711364	2.28E-05	**
Dark1 vs Light16	0.031276006	0.010845889	0.051706122	8.52E-05	**
Light10 vs Light13	0.027487325	0.008572712	0.046401937	0.000237117	**
Light9 vs Light16	0.026967207	0.008052594	0.045881819	0.000339111	**
Light11 vs Light13	0.026063196	0.007148584	0.044977809	0.000627179	**
Dark1 vs Dark16	0.029908486	0.008067773	0.051749199	0.000699386	**
Light10 vs Dark8	0.025128253	0.00621364	0.044042865	0.001172302	**
Light10 vs Dark9	0.024180416	0.005265804	0.043095029	0.002182902	**
Light11 vs Dark8	0.023704125	0.004789512	0.042618737	0.002967654	**
Light9 vs Dark16	0.025599687	0.005169571	0.046029804	0.002974164	**
Light11 vs Dark9	0.022756288	0.003841676	0.0416709	0.005404338	**
Dark11 vs Light16	0.024286718	0.003856601	0.044716834	0.006395407	**
Dark10 vs Light16	0.022452942	0.003538329	0.041367554	0.006523896	**
Light1 vs Light16	0.022430768	0.003516155	0.04134538	0.006613822	**
Light10 vs Dark13	0.022046888	0.003132276	0.0409615	0.008369251	**
Light10 vs Dark7	0.022967649	0.002537532	0.043397765	0.013393664	*
Light10 vs Dark5	0.021115635	0.002201022	0.040030247	0.014617795	*
Light10 vs Light7	0.022806889	0.002376772	0.043237005	0.014622599	*
Dark1 vs Light13	0.022690435	0.002260318	0.043120551	0.01557749	*
Light11 vs Dark13	0.02062276	0.001708147	0.039537372	0.019473986	*
Light11 vs Dark7	0.02154352	0.001113404	0.041973637	0.028587632	*
Light10 vs Light8	0.019898237	0.000983624	0.038812849	0.029348766	*
Dark11 vs Dark16	0.022919198	0.001078485	0.044759911	0.030169093	*
Light11 vs Light7	0.02138276	0.000952644	0.041812877	0.031051213	*
Light10 vs Light5	0.02127428	0.000844163	0.041704396	0.032820747	*
Light11 vs Dark5	0.019691506	0.000776894	0.038606119	0.032904829	*
Dark10 vs Dark16	0.021085422	0.000655306	0.041515539	0.036119986	*
Light1 vs Dark16	0.021063248	0.000633132	0.041493365	0.036526327	*
Dark1 vs Dark8	0.020331363	-9.88E-05	0.040761479	0.052458513	
Dark12 vs Light16	0.018551732	-0.000362881	0.037466344	0.060395863	
Light11 vs Light8	0.018474108	-0.000440504	0.037388721	0.062850489	
Light9 vs Light13	0.018381636	-0.000532977	0.037296248	0.065887594	
Light11 vs Light5	0.019850151	-0.000579965	0.040280268	0.066020667	
Light10 vs Light12	0.018268245	-0.000646368	0.037182857	0.069784697	
Dark1 vs Dark9	0.019383526	-0.00104659	0.039813643	0.081975482	
Light12 vs Light16	0.017804651	-0.001109962	0.036719263	0.087848986	
Light10 vs Dark12	0.017521164	-0.001393449	0.036435776	0.100735781	
Light11 vs Light12	0.016844117	-0.002070496	0.035758729	0.137931107	
Light8 vs Light16	0.016174659	-0.002739953	0.035089271	0.184702678	
Light11 vs Dark12	0.016097036	-0.002817577	0.035011648	0.190819856	
Light9 vs Dark8	0.016022564	-0.002892049	0.034937176	0.196828457	
Dark1 vs Dark13	0.017249998	-0.003180118	0.037680115	0.201119575	
Dark12 vs Dark16	0.017184212	-0.003245904	0.037614329	0.206215196	
Dark1 vs Dark7	0.018170759	-0.003669954	0.040011472	0.221209463	
Dark1 vs Light7	0.018009999	-0.003830714	0.039850712	0.233812978	
Light12 vs Dark16	0.016437131	-0.003992985	0.036867248	0.270624258	
Dark1 vs Dark5	0.016318745	-0.004111372	0.036748861	0.281930118	
Light9 vs Dark9	0.015074727	-0.003839885	0.03398934	0.285444065	
Dark5 vs Light16	0.014957261	-0.003957351	0.033871873	0.297983351	
Dark11 vs Light13	0.015701147	-0.00472897	0.036131263	0.345573779	
Dark1 vs Light5	0.01647739	-0.005363323	0.038318103	0.377746179	
Dark13 vs Light16	0.014026008	-0.004888605	0.03294062	0.408402083	
Dark1 vs Light8	0.015101347	-0.00532877	0.035531463	0.414161499	
Dark10 vs Light13	0.013867371	-0.005047242	0.032781983	0.428911721	
Light1 vs Light13	0.013845197	-0.005069416	0.032759809	0.431810936	
Light8 vs Dark16	0.01480714	-0.005622977	0.035237256	0.449813469	
Light5 vs Light16	0.014798616	-0.0056315	0.035228732	0.450862754	
Light10 vs Light1	0.013642128	-0.005272485	0.03255674	0.458697876	
Light10 vs Dark10	0.013619954	-0.005294658	0.032534566	0.461667829	
Light9 vs Dark13	0.012941199	-0.005973413	0.031855811	0.554822001	
Light9 vs Dark7	0.01386196	-0.006568157	0.034292076	0.569799583	
Light9 vs Light7	0.0137012	-0.006728917	0.034131316	0.590533134	
Dark5 vs Dark16	0.013589742	-0.006840375	0.034019858	0.6048807	
Dark1 vs Light12	0.013471355	-0.006958762	0.033901471	0.620070205	
Dark11 vs Dark8	0.013342075	-0.007088042	0.033772191	0.636570666	
Light7 vs Light16	0.013266007	-0.007164109	0.033696123	0.646224853	
Light11 vs Light1	0.012218	-0.006696613	0.031132612	0.654947757	
Light11 vs Dark10	0.012195826	-0.006718787	0.031110438	0.657962736	
Dark7 vs Light16	0.013105247	-0.007324869	0.033535363	0.666460549	
Light9 vs Dark5	0.012009946	-0.006904667	0.030924558	0.682998013	
Dark9 vs Light16	0.011892479	-0.007022133	0.030807092	0.698563465	
Dark1 vs Dark12	0.012724274	-0.007705843	0.03315439	0.713200023	
Dark13 vs Dark16	0.012658488	-0.007771628	0.033088605	0.721055291	
Light5 vs Dark16	0.013431097	-0.008409616	0.03527181	0.7322499	
Dark10 vs Dark8	0.011508299	-0.007406314	0.030422911	0.747697015	
Light1 vs Dark8	0.011486125	-0.007428487	0.030400737	0.750436498	
Dark11 vs Dark9	0.012394238	-0.008035878	0.032824355	0.751826854	
Light9 vs Light5	0.012168591	-0.008261526	0.032598707	0.776981078	
Dark8 vs Light16	0.010944643	-0.00796997	0.029859255	0.813325092	
Light10 vs Dark11	0.011786178	-0.008643939	0.032216294	0.816857667	
Light9 vs Light8	0.010792548	-0.008122065	0.02970716	0.829436736	
Dark10 vs Dark9	0.010560462	-0.00835415	0.029475075	0.852558094	
Light1 vs Dark9	0.010538289	-0.008376324	0.029452901	0.85467137	
Light7 vs Dark16	0.011898488	-0.009942225	0.033739201	0.875913687	
Dark7 vs Dark16	0.011737728	-0.010102985	0.033578441	0.887632082	
Dark12 vs Light13	0.009966161	-0.008948452	0.028880773	0.903165793	
Dark9 vs Dark16	0.01052496	-0.009905157	0.030955076	0.918798303	
Dark11 vs Dark7	0.011181471	-0.010659242	0.033022184	0.922770763	
Light11 vs Dark11	0.01036205	-0.010068067	0.030792166	0.928414566	
Dark11 vs Light7	0.011020711	-0.010820002	0.032861424	0.93137649	
Dark11 vs Dark13	0.01026071	-0.010169406	0.030690827	0.93399226	
Light12 vs Light13	0.00921908	-0.009695533	0.028133692	0.948873763	
Light9 vs Light12	0.009162556	-0.009752057	0.028077168	0.951557413	
Light10 vs Light9	0.009105689	-0.009808923	0.028020301	0.954154266	
Dark8 vs Dark16	0.009577123	-0.010852993	0.03000724	0.963978897	
Dark10 vs Dark7	0.009347695	-0.011082422	0.029777811	0.971321521	
Dark11 vs Dark5	0.009329457	-0.01110066	0.029759573	0.971852609	
Light1 vs Dark7	0.009325521	-0.011104596	0.029755637	0.971966242	
Light13 vs Light16	0.008585571	-0.010329041	0.027500183	0.973436409	
Dark10 vs Light7	0.009186934	-0.011243182	0.029617051	0.975750565	
Light1 vs Light7	0.009164761	-0.011265356	0.029594877	0.976317909	
Dark10 vs Dark13	0.008426934	-0.010487678	0.027341546	0.977865577	
Light9 vs Dark12	0.008415475	-0.010499138	0.027330087	0.978161997	
Light1 vs Dark13	0.00840476	-0.010509852	0.027319373	0.978436377	
Dark11 vs Light5	0.009488102	-0.012352611	0.031328815	0.982827469	
Dark1 vs Light1	0.008845238	-0.011584879	0.029275354	0.983413817	
Dark1 vs Dark10	0.008823064	-0.011607052	0.029253181	0.98383606	
Light11 vs Light9	0.007681561	-0.011233052	0.026596173	0.991607802	
Dark12 vs Dark8	0.007607089	-0.011307523	0.026521701	0.992467065	
Light8 vs Light13	0.007589088	-0.011325524	0.0265037	0.992663593	
Dark11 vs Light8	0.008112059	-0.012318058	0.028542175	0.993475016	
Dark10 vs Dark5	0.007495681	-0.011418932	0.026410293	0.993617226	
Light1 vs Dark5	0.007473507	-0.011441106	0.026388119	0.993827949	
Dark10 vs Light5	0.007654326	-0.012775791	0.028084442	0.996677428	
Light1 vs Light5	0.007632152	-0.012797965	0.028062268	0.996790706	
Light12 vs Dark8	0.006860008	-0.012054604	0.02577462	0.997758487	
Light13 vs Dark16	0.007218052	-0.013212065	0.027648168	0.998381208	
Dark12 vs Dark9	0.006659253	-0.01225536	0.025573865	0.99845096	
Dark5 vs Light13	0.00637169	-0.012542922	0.025286302	0.999120102	
Dark10 vs Light8	0.006278282	-0.01263633	0.025192895	0.999274868	
Light1 vs Light8	0.006256109	-0.012658504	0.025170721	0.99930792	
Dark1 vs Dark11	0.006989288	-0.014851425	0.028830001	0.999554726	
Dark11 vs Light12	0.006482067	-0.01394805	0.026912183	0.999603545	
Light12 vs Dark9	0.005912172	-0.013002441	0.024826784	0.99967673	
Light5 vs Light13	0.006213045	-0.014217071	0.026643161	0.999778833	
Dark13 vs Light13	0.005440437	-0.013474176	0.024355049	0.999899632	
Dark11 vs Dark12	0.005734986	-0.014695131	0.026165102	0.999929485	
Light8 vs Dark8	0.005230016	-0.013684596	0.024144629	0.999943473	
Dark12 vs Dark7	0.005446485	-0.014983632	0.025876601	0.999967118	
Dark12 vs Light7	0.005285725	-0.015144392	0.025715841	0.999979065	
Dark10 vs Light12	0.004648291	-0.014266322	0.023562903	0.999990489	
Light1 vs Light12	0.004626117	-0.014288495	0.023540729	0.99999117	
Light9 vs Light1	0.004536439	-0.014378174	0.023451051	0.999993492	
Dark12 vs Dark13	0.004525724	-0.014388888	0.023440337	0.999993729	
Light9 vs Dark10	0.004514265	-0.014400347	0.023428877	0.999993973	
Light10 vs Dark1	0.00479689	-0.015633227	0.025227006	0.99999534	
Light12 vs Dark7	0.004699404	-0.015730713	0.02512952	0.999996633	
Light7 vs Light13	0.004680436	-0.01574968	0.025110552	0.999996843	
Light8 vs Dark9	0.00428218	-0.014632433	0.023196792	0.999997387	
Light12 vs Light7	0.004538644	-0.015891473	0.02496876	0.99999807	
Dark7 vs Light13	0.004519676	-0.01591044	0.024949792	0.999998196	
Dark5 vs Dark8	0.004012618	-0.014901994	0.022927231	0.999999086	
Dark1 vs Light9	0.004308799	-0.016121317	0.024738916	0.999999169	
Dark10 vs Dark12	0.00390121	-0.015013403	0.022815822	0.999999424	
Light1 vs Dark12	0.003879036	-0.015035576	0.022793648	0.999999475	
Light12 vs Dark13	0.003778643	-0.015135969	0.022693256	0.99999966	
Dark12 vs Dark5	0.003594471	-0.015320142	0.022509083	0.999999852	
Light5 vs Dark8	0.003853973	-0.016576143	0.02428409	0.99999987	
Dark12 vs Light5	0.003753116	-0.016677001	0.024183232	0.999999917	
Dark9 vs Light13	0.003306908	-0.015607704	0.022221521	0.999999964	
Light11 vs Dark1	0.003372762	-0.017057355	0.023802878	0.999999987	
Dark13 vs Dark8	0.003081365	-0.015833248	0.021995977	0.999999989	
Dark5 vs Dark9	0.003064782	-0.015849831	0.021979394	0.99999999	
Light12 vs Dark5	0.00284739	-0.016067223	0.021762002	0.999999997	
Light8 vs Dark7	0.003069412	-0.017360704	0.023499528	0.999999997	
Light12 vs Light5	0.003006035	-0.017424082	0.023436151	0.999999998	
Light8 vs Light7	0.002908652	-0.017521464	0.023338768	0.999999999	
Light5 vs Dark9	0.002906137	-0.01752398	0.023336253	0.999999999	
Light9 vs Dark11	0.002680489	-0.017749628	0.023110605	1	
Dark12 vs Light8	0.002377073	-0.01653754	0.021291685	1	
Dark8 vs Light13	0.002359072	-0.016555541	0.021273684	1	
Light7 vs Dark8	0.002321364	-0.018108752	0.022751481	1	
Light8 vs Dark13	0.002148652	-0.016765961	0.021063264	1	
Dark13 vs Dark9	0.002133528	-0.016781084	0.021048141	1	
Dark7 vs Dark8	0.002160604	-0.018269512	0.022590721	1	
Dark16 vs Light16	0.001367519	-0.019062597	0.021797636	1	
Dark9 vs Dark8	0.000947837	-0.017966776	0.019862449	1	
Dark7 vs Dark9	0.001212768	-0.019217349	0.021642884	1	
Light7 vs Dark9	0.001373528	-0.019056589	0.021803644	1	
Light7 vs Dark7	0.00016076	-0.021679953	0.022001473	1	
Dark13 vs Dark7	0.000920761	-0.019509356	0.021350877	1	
Light5 vs Dark7	0.001693369	-0.020147344	0.023534082	1	
Dark5 vs Dark7	0.001852014	-0.018578102	0.02228213	1	
Dark13 vs Light7	0.00076	-0.019670116	0.021190117	1	
Light5 vs Light7	0.001532609	-0.020308104	0.023373322	1	
Dark5 vs Light7	0.001691254	-0.018738862	0.02212137	1	
Light5 vs Dark13	0.000772608	-0.019657508	0.021202725	1	
Dark5 vs Dark13	0.000931253	-0.017983359	0.019845866	1	
Dark5 vs Light5	0.000158645	-0.020271471	0.020588761	1	
Light8 vs Light5	0.001376043	-0.019054073	0.021806159	1	
Light8 vs Dark5	0.001217398	-0.017697214	0.02013201	1	
Light12 vs Light8	0.001629992	-0.017284621	0.020544604	1	
Dark12 vs Light12	0.000747081	-0.018167531	0.019661693	1	
Dark10 vs Light1	2.22E-05	-0.018892439	0.018936786	1	
Dark11 vs Light1	0.00185595	-0.018574167	0.022286066	1	
Dark11 vs Dark10	0.001833776	-0.01859634	0.022263893	1	
Light10 vs Light11	0.001424128	-0.017490484	0.020338741	1	

tableS70_Light_vs_Light

	diff	lwr	upr	p adj	
Light10 vs Light16	0.036072896	0.017158283	0.054987508	0.000000504	**
Light11 vs Light16	0.034648767	0.015734155	0.05356338	0.00000142	**
Light10 vs Light13	0.027487325	0.008572712	0.046401937	0.000237117	**
Light9 vs Light16	0.026967207	0.008052594	0.045881819	0.000339111	**
Light11 vs Light13	0.026063196	0.007148584	0.044977809	0.000627179	**
Light1 vs Light16	0.022430768	0.003516155	0.04134538	0.006613822	**
Light10 vs Light7	0.022806889	0.002376772	0.043237005	0.014622599	*
Light10 vs Light8	0.019898237	0.000983624	0.038812849	0.029348766	*
Light11 vs Light7	0.02138276	0.000952644	0.041812877	0.031051213	*
Light10 vs Light5	0.02127428	0.000844163	0.041704396	0.032820747	*
Light11 vs Light8	0.018474108	-0.000440504	0.037388721	0.062850489	
Light9 vs Light13	0.018381636	-0.000532977	0.037296248	0.065887594	
Light11 vs Light5	0.019850151	-0.000579965	0.040280268	0.066020667	
Light10 vs Light12	0.018268245	-0.000646368	0.037182857	0.069784697	
Light12 vs Light16	0.017804651	-0.001109962	0.036719263	0.087848986	
Light11 vs Light12	0.016844117	-0.002070496	0.035758729	0.137931107	
Light8 vs Light16	0.016174659	-0.002739953	0.035089271	0.184702678	
Light1 vs Light13	0.013845197	-0.005069416	0.032759809	0.431810936	
Light5 vs Light16	0.014798616	-0.0056315	0.035228732	0.450862754	
Light10 vs Light1	0.013642128	-0.005272485	0.03255674	0.458697876	
Light9 vs Light7	0.0137012	-0.006728917	0.034131316	0.590533134	
Light7 vs Light16	0.013266007	-0.007164109	0.033696123	0.646224853	
Light11 vs Light1	0.012218	-0.006696613	0.031132612	0.654947757	
Light9 vs Light5	0.012168591	-0.008261526	0.032598707	0.776981078	
Light9 vs Light8	0.010792548	-0.008122065	0.02970716	0.829436736	
Light12 vs Light13	0.00921908	-0.009695533	0.028133692	0.948873763	
Light9 vs Light12	0.009162556	-0.009752057	0.028077168	0.951557413	
Light10 vs Light9	0.009105689	-0.009808923	0.028020301	0.954154266	
Light13 vs Light16	0.008585571	-0.010329041	0.027500183	0.973436409	
Light1 vs Light7	0.009164761	-0.011265356	0.029594877	0.976317909	
Light11 vs Light9	0.007681561	-0.011233052	0.026596173	0.991607802	
Light8 vs Light13	0.007589088	-0.011325524	0.0265037	0.992663593	
Light1 vs Light5	0.007632152	-0.012797965	0.028062268	0.996790706	
Light1 vs Light8	0.006256109	-0.012658504	0.025170721	0.99930792	
Light5 vs Light13	0.006213045	-0.014217071	0.026643161	0.999778833	
Light1 vs Light12	0.004626117	-0.014288495	0.023540729	0.99999117	
Light9 vs Light1	0.004536439	-0.014378174	0.023451051	0.999993492	
Light7 vs Light13	0.004680436	-0.01574968	0.025110552	0.999996843	
Light12 vs Light7	0.004538644	-0.015891473	0.02496876	0.99999807	
Light12 vs Light5	0.003006035	-0.017424082	0.023436151	0.999999998	
Light8 vs Light7	0.002908652	-0.017521464	0.023338768	0.999999999	
Light5 vs Light7	0.001532609	-0.020308104	0.023373322	1	
Light8 vs Light5	0.001376043	-0.019054073	0.021806159	1	
Light12 vs Light8	0.001629992	-0.017284621	0.020544604	1	
Light10 vs Light11	0.001424128	-0.017490484	0.020338741	1	

tableS71_Dark_vs_Dark

	diff	lwr	upr	p adj	
Dark1 vs Dark16	0.029908486	0.008067773	0.051749199	0.000699386	**
Dark11 vs Dark16	0.022919198	0.001078485	0.044759911	0.030169093	*
Dark10 vs Dark16	0.021085422	0.000655306	0.041515539	0.036119986	*
Dark1 vs Dark8	0.020331363	-9.88E-05	0.040761479	0.052458513	
Dark1 vs Dark9	0.019383526	-0.00104659	0.039813643	0.081975482	
Dark1 vs Dark13	0.017249998	-0.003180118	0.037680115	0.201119575	
Dark12 vs Dark16	0.017184212	-0.003245904	0.037614329	0.206215196	
Dark1 vs Dark7	0.018170759	-0.003669954	0.040011472	0.221209463	
Dark1 vs Dark5	0.016318745	-0.004111372	0.036748861	0.281930118	
Dark5 vs Dark16	0.013589742	-0.006840375	0.034019858	0.6048807	
Dark11 vs Dark8	0.013342075	-0.007088042	0.033772191	0.636570666	
Dark1 vs Dark12	0.012724274	-0.007705843	0.03315439	0.713200023	
Dark13 vs Dark16	0.012658488	-0.007771628	0.033088605	0.721055291	
Dark10 vs Dark8	0.011508299	-0.007406314	0.030422911	0.747697015	
Dark11 vs Dark9	0.012394238	-0.008035878	0.032824355	0.751826854	
Dark10 vs Dark9	0.010560462	-0.00835415	0.029475075	0.852558094	
Dark7 vs Dark16	0.011737728	-0.010102985	0.033578441	0.887632082	
Dark9 vs Dark16	0.01052496	-0.009905157	0.030955076	0.918798303	
Dark11 vs Dark7	0.011181471	-0.010659242	0.033022184	0.922770763	
Dark11 vs Dark13	0.01026071	-0.010169406	0.030690827	0.93399226	
Dark8 vs Dark16	0.009577123	-0.010852993	0.03000724	0.963978897	
Dark10 vs Dark7	0.009347695	-0.011082422	0.029777811	0.971321521	
Dark11 vs Dark5	0.009329457	-0.01110066	0.029759573	0.971852609	
Dark10 vs Dark13	0.008426934	-0.010487678	0.027341546	0.977865577	
Dark1 vs Dark10	0.008823064	-0.011607052	0.029253181	0.98383606	
Dark12 vs Dark8	0.007607089	-0.011307523	0.026521701	0.992467065	
Dark10 vs Dark5	0.007495681	-0.011418932	0.026410293	0.993617226	
Dark12 vs Dark9	0.006659253	-0.01225536	0.025573865	0.99845096	
Dark1 vs Dark11	0.006989288	-0.014851425	0.028830001	0.999554726	
Dark11 vs Dark12	0.005734986	-0.014695131	0.026165102	0.999929485	
Dark12 vs Dark7	0.005446485	-0.014983632	0.025876601	0.999967118	
Dark12 vs Dark13	0.004525724	-0.014388888	0.023440337	0.999993729	
Dark5 vs Dark8	0.004012618	-0.014901994	0.022927231	0.999999086	
Dark10 vs Dark12	0.00390121	-0.015013403	0.022815822	0.999999424	
Dark12 vs Dark5	0.003594471	-0.015320142	0.022509083	0.999999852	
Dark13 vs Dark8	0.003081365	-0.015833248	0.021995977	0.999999989	
Dark5 vs Dark9	0.003064782	-0.015849831	0.021979394	0.99999999	
Dark13 vs Dark9	0.002133528	-0.016781084	0.021048141	1	
Dark7 vs Dark8	0.002160604	-0.018269512	0.022590721	1	
Dark9 vs Dark8	0.000947837	-0.017966776	0.019862449	1	
Dark7 vs Dark9	0.001212768	-0.019217349	0.021642884	1	
Dark13 vs Dark7	0.000920761	-0.019509356	0.021350877	1	
Dark5 vs Dark7	0.001852014	-0.018578102	0.02228213	1	
Dark5 vs Dark13	0.000931253	-0.017983359	0.019845866	1	
Dark11 vs Dark10	0.001833776	-0.01859634	0.022263893	1	

Table S72 Two-way ANOVA for data shown in fig8#

ZT vs Tissue			Tissue			ZT		
Degree of freedom	F value	*p* value	Degree of freedom	F value	*p* value	Degree of freedom	F value	*p* value
(2, 12)	6.168	0.0144	(1, 12)	2.473	1.42E-01	(2, 12)	5.714	1.81E-02

#Result of post hoc analysis is in tableS73.

tableS73_fig8_posthoc

	diff	lwr	upr	p adj	
PRL17-PRL13	-0.000463279	-0.000867145	-5.94E-05	0.021732315	*
PRL13-IRL17	0.000460008	5.61E-05	0.000863874	0.022755067	*
PRL13-IRL13	0.00044768	4.38E-05	0.000851546	0.027061992	*
PRL17-IRL1	-0.000318794	-0.000722659	8.51E-05	0.157672069	
IRL17-IRL1	-0.000315523	-0.000719388	8.83E-05	0.164422369	
IRL13-IRL1	-0.000303194	-0.00070706	0.000100671	0.192143717	
PRL13-PRL1	0.000261377	-0.000142489	0.000665243	0.315775172	
PRL17-PRL1	-0.000201902	-0.000605768	0.000201963	0.567999846	
PRL1-IRL17	0.000198631	-0.000205234	0.000602497	0.583564344	
PRL1-IRL13	0.000186303	-0.000217563	0.000590169	0.642481139	
PRL13-IRL1	0.000144486	-0.00025938	0.000548351	0.828070493	
PRL1-IRL1	-0.000116891	-0.000520757	0.000286974	0.918316647	
PRL17-IRL13	-1.56E-05	-0.000419465	0.000388266	0.999993263	
IRL17-IRL13	-1.23E-05	-0.000416194	0.000391537	0.999997913	
PRL17-IRL17	-3.27E-06	-0.000407137	0.000400595	0.999999997	

tableS74_zCry1aa

Matrix ID	Name	Score	Relative Score	Sequenc ID	Start	End	Strand	Predicted Sequence
MA0018.2	CREB1	9.47088	0.923179756	zCry1a_up_seq	-5643	-5636	+	TGACGTGA
MA0639.1	DBP	10.5696	0.919069375	zCry1a_up_seq	-5047	-5036	-	AATTGTGTAATG
MA0639.1	DBP	10.4502	0.917504333	zCry1a_up_seq	-5047	-5036	+	CATTACACAATT
MA0018.2	CREB1	9.00289	0.906041171	zCry1a_up_seq	-5643	-5636	-	TCACGTCA
MA0004.1	Arnt	8.60921	0.930502841	zCry1a_up_seq	-4040	-4035	+	AACGTG
MA0004.1	Arnt	8.60921	0.930502841	zCry1a_up_seq	-2711	-2706	+	AACGTG
MA0071.1	RORA	13.7006	0.981646591	zCry1a_up_seq	-2093	-2084	+	AACAAGGTCA
MA0004.1	Arnt	8.60921	0.930502841	zCry1a_up_seq	-780	-775	+	AACGTG
MA0004.1	Arnt	8.60921	0.930502841	zCry1a_up_seq	-127	-122	+	AACGTG
MA0639.1	DBP	10.662	0.920281277	zCry1a_up_seq	-1517	-1506	+	GCTTATATAAGG
MA0639.1	DBP	10.4376	0.917339143	zCry1a_up_seq	-1517	-1506	-	CCTTATATAAGC
MA0639.1	DBP	10.0846	0.912709583	zCry1a_up_seq	-329	-318	+	AGTTATACAACA
MA0639.1	DBP	9.76295	0.908492422	zCry1a_up_seq	-491	-480	-	GATTATGTAAAT
MA0639.1	DBP	9.50819	0.905152019	zCry1a_up_seq	-491	-480	+	ATTTACATAATC
MA1150.1	RORB	10.3126	0.90109106	zCry1a_up_seq	-2093	-2083	+	AACAAGGTCAC
MA0639.1	DBP	15.0389	0.977672966	zCry1a_Intron1	1991	2002	-	CATTATGTAATA
MA0639.1	DBP	14.9083	0.975960141	zCry1a_Intron1	1991	2002	+	TATTACATAATG
MA1151.1	RORC	13.8877	0.973330598	zCry1a_Intron1	1757	1768	-	AAAATTGGGTCA
MA1150.1	RORB	12.9103	0.958679639	zCry1a_Intron1	1756	1766	-	AATTGGGTCAG
MA0072.1	RORA(var.2)	15.7465	0.943533136	zCry1a_Intron1	1756	1769	-	TAAAATTGGGTCAG
MA0639.1	DBP	11.0859	0.925839966	zCry1a_Intron1	2085	2096	-	AATGACATAATG
MA0018.2	CREB1	9.00289	0.906041171	zCry1a_Intron1	1355	1362	+	TGACATCA
MA0639.1	DBP	9.33663	0.902902421	zCry1a_Intron1	2085	2096	+	CATTATGTCATT
MA1151.1	RORC	14.6275	0.988198918	zCry1a_Intron1	2724	2735	-	TAAAGTAGGTCA
MA1150.1	RORB	13.7983	0.978364423	zCry1a_Intron1	2723	2733	-	AAGTAGGTCAC
MA0072.1	RORA(var.2)	16.1484	0.951461721	zCry1a_Intron1	2723	2736	-	GTAAAGTAGGTCAC
MA1151.1	RORC	12.5683	0.946812847	zCry1a_Intron1	2649	2660	+	AGAAATGGGTCA
MA1150.1	RORB	12.14	0.941602317	zCry1a_Intron1	2651	2661	+	AAATGGGTCAG
MA0639.1	DBP	11.803	0.935241969	zCry1a_Intron1	2581	2592	+	GATGATGTAACA
MA0004.1	Arnt	8.60921	0.930502841	zCry1a_Intron1	2707	2712	+	AACGTG
MA0071.1	RORA	11.7947	0.929030327	zCry1a_Intron1	2724	2733	-	AAGTAGGTCA
MA0639.1	DBP	10.1861	0.91404142	zCry1a_Intron1	2581	2592	-	TGTTACATCATC
MA0639.1	DBP	15.5684	0.984615125	zCry1a_Intron1	4203	4214	+	GATTACATAACA
MA0639.1	DBP	14.453	0.969990053	zCry1a_Intron1	4203	4214	-	TGTTATGTAATC
MA0071.1	RORA	12.8171	0.957256746	zCry1a_Intron1	4395	4404	+	TTCTAGGTCA
MA1151.1	RORC	12.5217	0.94587642	zCry1a_Intron1	4537	4548	+	AAATTTAGGGCA
MA1151.1	RORC	11.7973	0.931319041	zCry1a_Intron1	4393	4404	+	GTTTCTAGGTCA
MA1150.1	RORB	10.7704	0.911240861	zCry1a_Intron1	4618	4628	-	TATTGGGTCAG
MA1150.1	RORB	10.7041	0.909771497	zCry1a_Intron1	4539	4549	+	ATTTAGGGCAT
MA1150.1	RORB	10.6798	0.909231328	zCry1a_Intron1	5642	5652	-	ATATGGGTTAT
MA0018.2	CREB1	9.00289	0.906041171	zCry1a_Intron1	5470	5477	-	TGACATCA
MA0476.1	FOS	8.83073	0.906714907	zCry1a_up_seq	-4273	-4263	-	TCTTATTCATA
MA0476.1	FOS	15.1627	0.991164914	zCry1a_up_seq	-3272	-3262	-	AGTGACTCATC
MA0476.1	FOS	12.8697	0.960583343	zCry1a_up_seq	-3272	-3262	+	GATGAGTCACT
MA0476.1	FOS	11.7743	0.945973961	zCry1a_up_seq	-2369	-2359	+	GTTGAGTCATT
MA0476.1	FOS	8.44849	0.901616971	zCry1a_up_seq	-2369	-2359	-	AATGACTCAAC
MA0476.1	FOS	11.5462	0.942931816	zCry1a_up_seq	-1168	-1158	-	GTTGAGTCATG
MA0476.1	FOS	9.03122	0.90938888	zCry1a_Intron1	1555	1565	-	AATTAGTCACT
MA0476.1	FOS	14.5686	0.983241138	zCry1a_Intron1	3741	3751	-	AATGACTCATG
MA0476.1	FOS	11.2838	0.939431599	zCry1a_Intron1	3109	3119	+	GGTGAGTCAGG
MA0476.1	FOS	11.2229	0.938619295	zCry1a_Intron1	3339	3349	+	GATTAGTCATT
MA0476.1	FOS	11.077	0.936673119	zCry1a_Intron1	3741	3751	+	CATGAGTCATT
MA0476.1	FOS	9.78508	0.919443274	zCry1a_Intron1	3109	3119	-	CCTGACTCACC
MA0476.1	FOS	10.7552	0.932382031	zCry1a_Intron1	5321	5331	+	TGTTAGTCATA
MA0476.1	FOS	10.7552	0.932382031	zCry1a_Intron1	5321	5331	+	TGTTAGTCATA

tableS75_zCry1ab

Matrix ID	Name	Score	Relative Score	Sequenc ID	Start	End	Strand	Predicted Sequence
MA1151.1	RORC	11.1382	0.91807155	zCry1b_up_seq	-2980	-2969	-	ATATATATGTCA
MA0071.1	RORA	11.1736	0.911882857	zCry1b_up_seq	-4461	-4452	+	GTAAAGGTCA
MA0071.1	RORA	10.7472	0.900113985	zCry1b_up_seq	-3425	-3416	+	AAAAGGGTCA
MA0639.1	DBP	12.356	0.942492962	zCry1b_up_seq	-696	-685	+	GATTATATAAGG
MA0639.1	DBP	11.5632	0.93209768	zCry1b_up_seq	-696	-685	-	CCTTATATAATC
MA0004.1	Arnt	8.60921	0.930502841	zCry1b_up_seq	-647	-642	+	AACGTG
MA0004.1	Arnt	8.60921	0.930502841	zCry1b_up_seq	-276	-271	+	AACGTG
MA1151.1	RORC	10.6775	0.908812415	zCry1b_up_seq	-1224	-1213	+	CTATATGGGTTA
MA0018.2	CREB1	8.89483	0.902083711	zCry1b_up_seq	-587	-580	-	TGACGCAA
MA0004.1	Arnt	10.3511	1.000000008	zCry1b_Intron2	182	187	+	CACGTG
MA0004.1	Arnt	10.3511	1.000000008	zCry1b_Intron2	182	187	-	CACGTG
MA1150.1	RORB	14.4717	0.99329329	zCry1b_Intron2	2014	2024	+	ATTTAGGTCAC
MA1151.1	RORC	14.4487	0.984604783	zCry1b_Intron2	2012	2023	+	AAATTTAGGTCA
MA0071.1	RORA	13.3874	0.97300131	zCry1b_Intron2	2543	2552	+	TTCAAGGTCA
MA0639.1	DBP	13.7444	0.960698744	zCry1b_Intron2	839	850	-	AATTATATAATA
MA0639.1	DBP	12.8729	0.949271369	zCry1b_Intron2	839	850	+	TATTATATAATT
MA0072.1	RORA(var.2)	15.6119	0.940877424	zCry1b_Intron2	2011	2024	+	CAAATTTAGGTCAC
MA0071.1	RORA	12.1546	0.9389677	zCry1b_Intron2	2014	2023	+	ATTTAGGTCA
MA0071.1	RORA	11.747	0.927713565	zCry1b_Intron2	1651	1660	-	TTTAAGGTCA
MA0018.2	CREB1	9.00289	0.906041171	zCry1b_Intron2	2350	2357	+	TGACATCA
MA1150.1	RORB	12.1872	0.942649462	zCry1b_Intron2	4053	4063	+	ATTTAGGTTAT
MA1151.1	RORC	11.8533	0.932444355	zCry1b_Intron2	4051	4062	+	GAATTTAGGTTA
MA0004.1	Arnt	8.60921	0.930502841	zCry1b_Intron2	5937	5942	+	AACGTG
MA0004.1	Arnt	10.3511	1.000000008	zCry1b_Intron2	7751	7756	+	CACGTG
MA0004.1	Arnt	10.3511	1.000000008	zCry1b_Intron2	7751	7756	-	CACGTG
MA1151.1	RORC	13.7053	0.969665584	zCry1b_Intron2	6391	6402	-	AGAAGTAGGTCA
MA1151.1	RORC	13.4767	0.96507112	zCry1b_Intron2	6339	6350	+	GGAAGTAGGTCA
MA1150.1	RORB	12.6715	0.953384865	zCry1b_Intron2	6341	6351	+	AAGTAGGTCAG
MA1150.1	RORB	12.6715	0.953384865	zCry1b_Intron2	6390	6400	-	AAGTAGGTCAG
MA0072.1	RORA(var.2)	15.5765	0.940178938	zCry1b_Intron2	6390	6403	-	GAGAAGTAGGTCAG
MA0639.1	DBP	11.6765	0.933583203	zCry1b_Intron2	6290	6301	+	GGTGATGTAACA
MA0071.1	RORA	11.7947	0.929030327	zCry1b_Intron2	6341	6350	+	AAGTAGGTCA
MA0071.1	RORA	11.7947	0.929030327	zCry1b_Intron2	6391	6400	-	AAGTAGGTCA
MA0072.1	RORA(var.2)	14.8504	0.925854936	zCry1b_Intron2	6338	6351	+	TGGAAGTAGGTCAG
MA0639.1	DBP	10.313	0.91570525	zCry1b_Intron2	6290	6301	-	TGTTACATCACC
MA0639.1	DBP	11.5278	0.931633285	zCry1b_Intron2	11173	11184	-	GATTATGCAATA
MA0639.1	DBP	11.0582	0.925476348	zCry1b_Intron2	11173	11184	+	TATTGCATAATC
MA0004.1	Arnt	8.60921	0.930502841	zCry1b_Intron2	12345	12350	+	AACGTG
MA0018.2	CREB1	9.47088	0.923179756	zCry1b_Intron2	12326	12333	-	TGACGTGA
MA0018.2	CREB1	9.00289	0.906041171	zCry1b_Intron2	12326	12333	+	TCACGTCA
MA0476.1	FOS	9.70866	0.918424028	zCry1b_up_seq	-182	-172	+	TGTGATTCAGC
MA0476.1	FOS	9.70866	0.918424028	zCry1b_up_seq	187	197	+	TGTGATTCAGC
MA0476.1	FOS	8.67907	0.904692275	zCry1b_up_seq	-182	-172	-	GCTGAATCACA
MA0476.1	FOS	8.67907	0.904692275	zCry1b_up_seq	187	197	-	GCTGAATCACA
MA0476.1	FOS	8.35422	0.900359697	zCry1b_up_seq	-1430	-1420	+	TCTGACTCAAA
MA0476.1	FOS	13.5725	0.969956277	zCry1b_Intron2	5003	5013	+	TCTGATTCATT
MA0476.1	FOS	10.0304	0.922715358	zCry1b_Intron2	3532	3542	-	TCTTATTCATT
MA0476.1	FOS	10.0218	0.922600668	zCry1b_Intron2	4364	4374	-	GGTTACTCACC
MA0476.1	FOS	10.7729	0.932617388	zCry1b_Intron2	6992	7002	+	TTTGAGTCATA
MA0476.1	FOS	9.91081	0.921120132	zCry1b_Intron2	6879	6889	-	ATTGACTCACT
MA0476.1	FOS	8.82191	0.906597343	zCry1b_Intron2	6879	6889	+	AGTGAGTCAAT
MA0476.1	FOS	8.34039	0.900175242	zCry1b_Intron2	7275	7285	-	GTTGAATCATT
MA0476.1	FOS	13.6633	0.971167482	zCry1b_Intron2	9163	9173	+	TGTGATTCATT

tableS76_zCry1ba

Matrix ID	Name	Score	Relative Score	Sequenc ID	Start	End	Strand	Predicted Sequence
MA0639.1	DBP	10.7478	0.92140557	zCry2a_up_seq	-9310	-9299	-	AATTGCATAATC
MA0639.1	DBP	10.5993	0.919458891	zCry2a_up_seq	-8062	-8051	+	GATTACACAATT
MA0639.1	DBP	10.4943	0.918081798	zCry2a_up_seq	-8062	-8051	-	AATTGTGTAATC
MA0639.1	DBP	10.3458	0.916135132	zCry2a_up_seq	-9310	-9299	+	GATTATGCAATT
MA1151.1	RORC	12.4151	0.943735196	zCry2a_up_seq	-7233	-7222	-	TAATGTAGGGCA
MA0639.1	DBP	11.1429	0.926586985	zCry2a_up_seq	-5633	-5622	+	TATGATGTAATG
MA0639.1	DBP	10.7721	0.921724345	zCry2a_up_seq	-5633	-5622	-	CATTACATCATA
MA1151.1	RORC	10.9485	0.914260256	zCry2a_up_seq	-7017	-7006	-	TCATGTAGGGCA
MA1151.1	RORC	10.5229	0.905706168	zCry2a_up_seq	-6578	-6567	-	CAAAATAGGACA
MA1151.1	RORC	12.4151	0.943735196	zCry2a_up_seq	-7233	-7222	-	TAATGTAGGGCA
MA0639.1	DBP	11.1429	0.926586985	zCry2a_up_seq	-5633	-5622	+	TATGATGTAATG
MA0639.1	DBP	10.7721	0.921724345	zCry2a_up_seq	-5633	-5622	-	CATTACATCATA
MA1151.1	RORC	10.9485	0.914260256	zCry2a_up_seq	-7017	-7006	-	TCATGTAGGGCA
MA1151.1	RORC	10.5229	0.905706168	zCry2a_up_seq	-6578	-6567	-	CAAAATAGGACA
MA0004.1	Arnt	10.3511	1.000000008	zCry2a_up_seq	-4723	-4718	+	CACGTG
MA0004.1	Arnt	10.3511	1.000000008	zCry2a_up_seq	-4723	-4718	-	CACGTG
MA1151.1	RORC	14.5054	0.985745679	zCry2a_up_seq	-4224	-4213	-	GAAATTAGGTCA
MA1150.1	RORB	14.125	0.985607567	zCry2a_up_seq	-4225	-4215	-	AATTAGGTCAA
MA0072.1	RORA(var.2)	16.5285	0.958961074	zCry2a_up_seq	-4225	-4212	-	AGAAATTAGGTCAA
MA0639.1	DBP	13.3827	0.955955651	zCry2a_up_seq	-4418	-4407	-	GGTTATGTAAGG
MA0639.1	DBP	13.0968	0.952206712	zCry2a_up_seq	-4418	-4407	+	CCTTACATAACC
MA1150.1	RORB	12.196	0.942845191	zCry2a_up_seq	-4399	-4389	-	AATGGGGTCAA
MA0004.1	Arnt	8.60921	0.930502841	zCry2a_up_seq	-4742	-4737	+	AACGTG
MA0071.1	RORA	11.4898	0.920614282	zCry2a_up_seq	-4224	-4215	-	AATTAGGTCA
MA0018.2	CREB1	9.00289	0.906041171	zCry2a_up_seq	-2881	-2874	+	TGACATCA
MA0639.1	DBP	9.51888	0.905292099	zCry2a_up_seq	-4497	-4486	+	ACTGACATAACG
MA0018.2	CREB1	9.92666	0.939870952	zCry2a_up_seq	-1729	-1722	-	TGACGTCT
MA1151.1	RORC	11.2709	0.920739705	zCry2a_up_seq	-1634	-1623	+	AAAAATAGGACA
MA0018.2	CREB1	9.14267	0.911160167	zCry2a_up_seq	-70	-63	-	GGACGTCA
MA1151.1	RORC	10.5041	0.90532799	zCry2a_up_seq	-898	-887	-	TTAATTCGGTCA
MA0004.1	Arnt	10.3511	1.000000008	zCry2a_Intron2	883	888	+	CACGTG
MA0004.1	Arnt	10.3511	1.000000008	zCry2a_Intron2	883	888	-	CACGTG
MA1150.1	RORB	11.8374	0.934895599	zCry2a_Intron2	430	440	+	ATATGGGTCAG
MA1151.1	RORC	11.7657	0.930682132	zCry2a_Intron2	428	439	+	GCATATGGGTCA
MA0004.1	Arnt	8.60921	0.930502841	zCry2a_Intron2	1246	1251	+	AACGTG
MA0639.1	DBP	11.3767	0.929652793	zCry2a_Intron2	397	408	-	AGTTGCATAACA
MA0018.2	CREB1	9.47088	0.923179756	zCry2a_Intron2	1173	1180	-	TGACGTTA
MA0639.1	DBP	10.3663	0.91640365	zCry2a_Intron2	397	408	+	TGTTATGCAACT
MA0071.1	RORA	11.1736	0.911882857	zCry2a_Intron2	2100	2109	+	GTAAAGGTCA
MA0018.2	CREB1	9.00289	0.906041171	zCry2a_Intron2	268	275	+	TGACATCA
MA0071.1	RORA	10.8418	0.90272284	zCry2a_Intron2	430	439	+	ATATGGGTCA
MA0004.1	Arnt	10.3511	1.000000008	zCry2a_Intron2	4507	4512	+	CACGTG
MA0004.1	Arnt	10.3511	1.000000008	zCry2a_Intron2	4507	4512	-	CACGTG
MA1150.1	RORB	14.4717	0.99329329	zCry2a_Intron2	3543	3553	+	ATTTAGGTCAC
MA1151.1	RORC	14.7801	0.991265146	zCry2a_Intron2	3541	3552	+	ATATTTAGGTCA
MA1151.1	RORC	14.6386	0.988421443	zCry2a_Intron2	3391	3402	-	ATATATAGGTCA
MA1150.1	RORB	13.0521	0.961823506	zCry2a_Intron2	3390	3400	-	ATATAGGTCAA
MA0071.1	RORA	12.9252	0.96023922	zCry2a_Intron2	3391	3400	-	ATATAGGTCA
MA1151.1	RORC	12.7348	0.95015954	zCry2a_Intron2	4147	4158	+	ATAAGTAGGCCA
MA0072.1	RORA(var.2)	15.8529	0.945632129	zCry2a_Intron2	3540	3553	+	GATATTTAGGTCAC
MA0071.1	RORA	12.1546	0.9389677	zCry2a_Intron2	3543	3552	+	ATTTAGGTCA
MA0072.1	RORA(var.2)	15.4054	0.936804497	zCry2a_Intron2	3390	3403	-	CATATATAGGTCAA
MA0071.1	RORA	12.0601	0.936358846	zCry2a_Intron2	2914	2923	+	AATAAGGTCA
MA0004.1	Arnt	8.60921	0.930502841	zCry2a_Intron2	4432	4437	-	AACGTG
MA0018.2	CREB1	9.47088	0.923179756	zCry2a_Intron2	2694	2701	+	TGACGTTA
MA0639.1	DBP	10.4634	0.917676851	zCry2a_Intron2	3610	3621	+	TGTTACATAAAC
MA0639.1	DBP	10.3005	0.915541598	zCry2a_Intron2	3610	3621	-	GTTTATGTAACA
MA1150.1	RORB	10.9374	0.914943057	zCry2a_Intron2	2914	2924	+	AATAAGGTCAA
MA1150.1	RORB	10.8004	0.91190604	zCry2a_Intron2	4149	4159	+	AAGTAGGCCAA
MA0639.1	DBP	9.94743	0.910911375	zCry2a_Intron2	4765	4776	-	CATGATATAACG
MA0639.1	DBP	9.81866	0.909222935	zCry2a_Intron2	3056	3067	+	ACTGATGTAACA
MA0071.1	RORA	10.9464	0.905611928	zCry2a_Intron2	3524	3533	+	ATGAGGGTCA
MA0072.1	RORA(var.2)	13.5689	0.900573358	zCry2a_Intron2	4146	4159	+	GATAAGTAGGCCAA
MA0004.1	Arnt	10.3511	1.000000008	zCry2a_Intron2	7454	7459	+	CACGTG
MA0004.1	Arnt	10.3511	1.000000008	zCry2a_Intron2	7454	7459	-	CACGTG
MA1151.1	RORC	13.0036	0.955561209	zCry2a_Intron2	5645	5656	+	CAATGTGGGTCA
MA1150.1	RORB	12.7586	0.955316915	zCry2a_Intron2	5647	5657	+	ATGTGGGTCAC
MA0639.1	DBP	9.68526	0.907473808	zCry2a_Intron2	5433	5444	-	TGTTATATAAAA
MA0639.1	DBP	9.16742	0.900683597	zCry2a_Intron2	5433	5444	+	TTTTATATAACA
MA0639.1	DBP	9.14258	0.900357994	zCry2a_Intron2	7478	7489	-	TGTTATACAAGA
MA1150.1	RORB	10.2669	0.900079242	zCry2a_Intron2	6770	6780	-	ATTGGGGTTAC
MA1150.1	RORB	12.812	0.956501079	zCry2a_Intron2	7692	7702	+	AAAGAGGTCAC
MA0639.1	DBP	13.3458	0.95547206	zCry2a_Intron2	7753	7764	+	AGTTACATAAGG
MA0639.1	DBP	12.2899	0.941626958	zCry2a_Intron2	7753	7764	-	CCTTATGTAACT
MA1150.1	RORB	11.9652	0.93772714	zCry2a_Intron2	8553	8563	-	AATCAGGTCAG
MA0071.1	RORA	12.0601	0.936358846	zCry2a_Intron2	8706	8715	-	AATAAGGTCA
MA0071.1	RORA	11.8598	0.93082823	zCry2a_Intron2	7692	7701	+	AAAGAGGTCA
MA0004.1	Arnt	8.60921	0.930502841	zCry2a_Intron2	9104	9109	+	AACGTG
MA1151.1	RORC	11.7455	0.930276546	zCry2a_Intron2	7690	7701	+	GAAAAGAGGTCA
MA1150.1	RORB	11.578	0.929143341	zCry2a_Intron2	8705	8715	-	AATAAGGTCAT
MA1151.1	RORC	11.4124	0.923583388	zCry2a_Intron2	7737	7748	+	AAAACTAGGACA
MA1150.1	RORB	11.2979	0.922934615	zCry2a_Intron2	8069	8079	-	AATGAGGTTAT
MA1151.1	RORC	10.5764	0.906781591	zCry2a_Intron2	8706	8717	-	TAAATAAGGTCA
MA1151.1	RORC	10.2643	0.900508625	zCry2a_Intron2	8554	8565	-	ACAATCAGGTCA
MA0476.1	FOS	9.64422	0.917564498	zCry2a_up_seq	-6168	-6158	+	ATTGATTCATT
MA0476.1	FOS	9.58744	0.916807256	zCry2a_up_seq	-6176	-6166	+	TATTATTCATT
MA0476.1	FOS	12.8485	0.960300237	zCry2a_up_seq	-15	-5	-	ACTGAGTCACC
MA0476.1	FOS	11.9549	0.948382482	zCry2a_up_seq	-1457	-1447	+	TGTTAGTCATT
MA0476.1	FOS	11.84	0.946850331	zCry2a_up_seq	-15	-5	+	GGTGACTCAGT
MA0476.1	FOS	10.0773	0.923340968	zCry2a_up_seq	-629	-619	-	TTTGAGTCACT
MA0476.1	FOS	9.90955	0.921103305	zCry2a_Intron2	1076	1086	+	TTTGAGTCACC
MA0476.1	FOS	11.2509	0.938993382	zCry2a_Intron2	4509	4519	-	GCTGATTCACG
MA0476.1	FOS	10.5684	0.92989016	zCry2a_Intron2	3696	3706	-	TGTGATTCACA
MA0476.1	FOS	9.9829	0.922081531	zCry2a_Intron2	3976	3986	+	ACTGATTCACA
MA0476.1	FOS	9.16111	0.911121245	zCry2a_Intron2	3157	3167	+	GATTATTCATG
MA0476.1	FOS	8.96812	0.908547373	zCry2a_Intron2	3696	3706	+	TGTGAATCACA
MA0476.1	FOS	8.43049	0.901376921	zCry2a_Intron2	3087	3097	-	TTTTAGTCATT
MA0476.1	FOS	8.35422	0.900359697	zCry2a_Intron2	2996	3006	-	TCTGACTCAAA
MA0476.1	FOS	13.1686	0.964570059	zCry2a_Intron2	6535	6545	-	AGTGATTCATT
MA0476.1	FOS	9.13935	0.910831093	zCry2a_Intron2	6535	6545	+	AATGAATCACT
MA0476.1	FOS	8.76917	0.905893954	zCry2a_Intron2	9355	9365	+	TCTTAGTCACA

tableS77_zCry1bb

Matrix ID	Name	Score	Relative Score	Sequenc ID	Start	End	Strand	Predicted Sequence
MA0071.1	RORA	12.059	0.936326199	zCry2b_up_seq	-5009	-5000	-	ATGGAGGTCA
MA0018.2	CREB1	9.77186	0.934202236	zCry2b_up_seq	-4507	-4500	-	TGAGGTCA
MA0004.1	Arnt	8.60921	0.930502841	zCry2b_up_seq	-4354	-4349	-	AACGTG
MA0004.1	Arnt	8.60921	0.930502841	zCry2b_up_seq	-4345	-4340	+	AACGTG
MA0004.1	Arnt	8.60921	0.930502841	zCry2b_up_seq	-4125	-4120	+	AACGTG
MA1151.1	RORC	11.5628	0.926605667	zCry2b_up_seq	-5009	-4998	-	CTATGGAGGTCA
MA1150.1	RORB	11.1771	0.920256583	zCry2b_up_seq	-5010	-5000	-	ATGGAGGTCAG
MA1150.1	RORB	11.1182	0.918950918	zCry2b_up_seq	-4649	-4639	-	TAATGGGTCAT
MA0018.2	CREB1	9.00289	0.906041171	zCry2b_up_seq	-4932	-4925	-	TGACATCA
MA0018.2	CREB1	9.00289	0.906041171	zCry2b_up_seq	-4913	-4906	-	TGACATCA
MA1151.1	RORC	10.4333	0.903905707	zCry2b_up_seq	-5491	-5480	+	ATATTTGTGTCA
MA0004.1	Arnt	8.60921	0.930502841	zCry2b_UTR	-3650	-3645	+	AACGTG
MA0004.1	Arnt	10.3511	1.000000008	zCry2b_Intron1	-1722	-1717	+	CACGTG
MA0004.1	Arnt	10.3511	1.000000008	zCry2b_Intron1	-1722	-1717	-	CACGTG
MA1150.1	RORB	12.7544	0.955222623	zCry2b_Intron1	-2967	-2957	-	AACTGGGTCAT
MA1151.1	RORC	12.7098	0.949656549	zCry2b_Intron1	-2966	-2955	-	AGAACTGGGTCA
MA0018.2	CREB1	10.1001	0.94622179	zCry2b_Intron1	-2990	-2983	+	TGACGTAA
MA0639.1	DBP	11.092	0.92591901	zCry2b_Intron1	-2792	-2781	+	TGTTACATAAAA
MA0639.1	DBP	10.6689	0.920371313	zCry2b_Intron1	-2992	-2981	+	TGTGACGTAAGT
MA1151.1	RORC	11.1731	0.918774028	zCry2b_Intron1	-2812	-2801	+	TTATGTATGTCA
MA0639.1	DBP	10.3206	0.915805039	zCry2b_Intron1	-2792	-2781	-	TTTTATGTAACA
MA0639.1	DBP	10.2169	0.914445141	zCry2b_Intron1	-2992	-2981	-	ACTTACGTCACA
MA0639.1	DBP	9.76295	0.908492422	zCry2b_Intron1	-3103	-3092	+	GATTATGTAAAT
MA0071.1	RORA	11.0469	0.908385621	zCry2b_Intron1	-2966	-2957	-	AACTGGGTCA
MA0639.1	DBP	9.50819	0.905152019	zCry2b_Intron1	-3103	-3092	-	ATTTACATAATC
MA0004.1	Arnt	8.60921	0.930502841	zCry2b_Intron1	-1418	-1413	+	AACGTG
MA0004.1	Arnt	8.60921	0.930502841	zCry2b_Intron1	-732	-727	+	AACGTG
MA0639.1	DBP	9.14732	0.900420156	zCry2b_Intron1	-539	-528	-	GTTTATATAACA
MA0018.2	CREB1	10.3633	0.955861902	zCry2b_Intron2	4450	4457	+	TGACGCCA
MA0018.2	CREB1	10.1001	0.94622179	zCry2b_Intron2	3015	3022	+	TGACGTAA
MA1151.1	RORC	12.232	0.94005389	zCry2b_Intron2	4123	4134	+	GTTAGTAGGTCA
MA0004.1	Arnt	8.60921	0.930502841	zCry2b_Intron2	4185	4190	-	AACGTG
MA0018.2	CREB1	9.47088	0.923179756	zCry2b_Intron2	4385	4392	+	TGACGTTA
MA0018.2	CREB1	9.47088	0.923179756	zCry2b_Intron2	4462	4469	-	TGACGTGA
MA0018.2	CREB1	9.00289	0.906041171	zCry2b_Intron2	3705	3712	+	TGACATCA
MA0018.2	CREB1	9.00289	0.906041171	zCry2b_Intron2	4462	4469	+	TCACGTCA
MA1150.1	RORB	10.5316	0.905946087	zCry2b_Intron2	4125	4135	+	TAGTAGGTCAG
MA0071.1	RORA	10.8167	0.902031628	zCry2b_Intron2	4125	4134	+	TAGTAGGTCA
MA0071.1	RORA	10.7472	0.900113985	zCry2b_Intron2	3446	3455	-	AAAAGGGTCA
MA0476.1	FOS	10.9028	0.934350097	zCry2b_Intron1	-1232	-1222	+	GTTGACTCATA
MA0476.1	FOS	8.61501	0.903837795	zCry2b_Intron1	-1232	-1222	-	TATGAGTCAAC

tableS78_zCry2

Matrix ID	Name	Score	Relative Score	Sequenc ID	Start	End	Strand	Predicted Sequence
MA0639.1	DBP	14.8976	0.975820186	zCry3_up_seq	-9128	-9117	+	AATTATGTAATA
MA0639.1	DBP	14.2796	0.96771657	zCry3_up_seq	-9128	-9117	-	TATTACATAATT
MA0639.1	DBP	11.8231	0.935505397	zCry3_up_seq	-7728	-7717	+	TATGATGTAACA
MA0639.1	DBP	10.8147	0.922283578	zCry3_up_seq	-7728	-7717	-	TGTTACATCATA
MA1150.1	RORB	10.4741	0.904673107	zCry3_up_seq	-9579	-9569	-	ATGTGGGTTAT
MA0639.1	DBP	12.6893	0.946864421	zCry3_up_seq	-6058	-6047	+	AATTATATAACT
MA0639.1	DBP	12.436	0.943541825	zCry3_up_seq	-6058	-6047	-	AGTTATATAATT
MA1150.1	RORB	11.2337	0.921512481	zCry3_up_seq	-5326	-5316	+	AATGAGGCCAC
MA0004.1	Arnt	10.3511	1.000000008	zCry3_up_seq	-4395	-4390	+	CACGTG
MA0004.1	Arnt	10.3511	1.000000008	zCry3_up_seq	-4395	-4390	-	CACGTG
MA1151.1	RORC	12.5787	0.947023105	zCry3_up_seq	-3621	-3610	-	TTAATTAGGTCG
MA0018.2	CREB1	9.92666	0.939870952	zCry3_up_seq	-4899	-4892	-	TGACGTCT
MA1151.1	RORC	11.8256	0.93188716	zCry3_up_seq	-4809	-4798	+	CGATGTGGGTCA
MA1150.1	RORB	11.6318	0.930337357	zCry3_up_seq	-4807	-4797	+	ATGTGGGTCAG
MA1150.1	RORB	11.0934	0.918400327	zCry3_up_seq	-3622	-3612	-	AATTAGGTCGT
MA0018.2	CREB1	9.29746	0.916828884	zCry3_up_seq	-3782	-3775	+	TGACGTCG
MA0018.2	CREB1	9.14267	0.911160167	zCry3_up_seq	-3782	-3775	-	CGACGTCA
MA0639.1	DBP	9.61388	0.906537865	zCry3_up_seq	-3026	-3015	+	CATTATGTAAAT
MA0639.1	DBP	9.58351	0.906139595	zCry3_up_seq	-3026	-3015	-	ATTTACATAATG
MA0004.1	Arnt	10.3511	1.000000008	zCry3_up_seq	-185	-180	+	CACGTG
MA0004.1	Arnt	10.3511	1.000000008	zCry3_up_seq	-185	-180	-	CACGTG
MA0071.1	RORA	12.5246	0.949181648	zCry3_up_seq	-634	-625	-	ATAGAGGTCA
MA0004.1	Arnt	8.60921	0.930502841	zCry3_up_seq	-267	-262	+	AACGTG
MA1150.1	RORB	11.3827	0.924814825	zCry3_up_seq	-635	-625	-	ATAGAGGTCAG
MA0639.1	DBP	9.64261	0.906914576	zCry3_up_seq	-825	-814	+	CATTATATAAAA
MA0018.2	CREB1	9.00289	0.906041171	zCry3_up_seq	-313	-306	-	TGACATCA
MA1151.1	RORC	10.5108	0.905461582	zCry3_up_seq	-634	-623	-	AGATAGAGGTCA
MA0004.1	Arnt	10.3511	1.000000008	zCry3_Intron1	775	780	+	CACGTG
MA0004.1	Arnt	10.3511	1.000000008	zCry3_Intron1	775	780	-	CACGTG
MA0004.1	Arnt	10.3511	1.000000008	zCry3_Intron1	799	804	+	CACGTG
MA0004.1	Arnt	10.3511	1.000000008	zCry3_Intron1	799	804	-	CACGTG
MA0639.1	DBP	14.336	0.968456186	zCry3_Intron1	931	942	-	TATTACATAAGA
MA0639.1	DBP	13.5141	0.95767928	zCry3_Intron1	931	942	+	TCTTATGTAATA
MA0018.2	CREB1	9.92666	0.939870952	zCry3_Intron1	1376	1383	-	TGACGTCT
MA0639.1	DBP	10.3089	0.915651554	zCry3_Intron1	556	567	-	AATTACACAATT
MA0639.1	DBP	9.9409	0.910825803	zCry3_Intron1	556	567	+	AATTGTGTAATT
MA0639.1	DBP	9.51611	0.905255822	zCry3_Intron1	640	651	-	CGTTATATAAAA
MA0639.1	DBP	9.53091	0.905449924	zCry3_Intron2	3369	3380	+	AATTATACAATG
MA0639.1	DBP	9.90062	0.910297595	zCry3_Intron2	5371	5382	-	GGTTACACAAGC
MA0639.1	DBP	9.21757	0.901341293	zCry3_Intron2	5371	5382	+	GCTTGTGTAACC
MA0018.2	CREB1	11.5686	1.000000016	zCry3_Intron2	7117	7124	+	TGACGTCA
MA0018.2	CREB1	11.5686	1.000000016	zCry3_Intron2	7117	7124	-	TGACGTCA
MA1150.1	RORB	11.9572	0.937550417	zCry3_Intron2	8408	8418	-	AAGGAGGTCAA
MA1151.1	RORC	12.009	0.935573487	zCry3_Intron2	8409	8420	-	TAAAGGAGGTCA
MA0071.1	RORA	11.3941	0.917972781	zCry3_Intron2	8409	8418	-	AAGGAGGTCA
MA0018.2	CREB1	9.00289	0.906041171	zCry3_Intron2	7137	7144	+	TGACATCA
MA0071.1	RORA	10.8084	0.901801075	zCry3_Intron2	7387	7396	-	GACTAGGTCA
MA0476.1	FOS	8.52098	0.902583776	zCry3_up_seq	-8533	-8523	+	TGTTAATCATT
MA0476.1	FOS	9.63457	0.917435918	zCry3_up_seq	-5037	-5027	-	ACTTACTCACC
MA0476.1	FOS	10.2779	0.926015615	zCry3_up_seq	-3559	-3549	+	ACTGAATCATA
MA0476.1	FOS	9.34264	0.913542384	zCry3_up_seq	-3559	-3549	-	TATGATTCAGT
MA0476.1	FOS	10.1389	0.924161921	zCry3_up_seq	-2377	-2367	-	TTTGATTCATT
MA0476.1	FOS	13.1295	0.964048175	zCry3_Intron1	1059	1069	+	TATGATTCATT
MA0476.1	FOS	10.2782	0.926019965	zCry3_Intron1	1007	1017	-	ATTGAGTCATA
MA0476.1	FOS	9.83489	0.920107513	zCry3_Intron1	1059	1069	-	AATGAATCATA
MA0476.1	FOS	9.11093	0.910452058	zCry3_Intron1	1007	1017	+	TATGACTCAAT
MA0476.1	FOS	8.39263	0.900871978	zCry3_Intron1	1163	1173	+	GTTTACTCATC
MA0476.1	FOS	11.6206	0.943923995	zCry3_Intron2	5548	5558	+	AGTTACTCATC

tableS79_zCry4

Matrix ID	Name	Score	Relative Score	Sequenc ID	Start	End	Strand	Predicted Sequence
MA0004.1	Arnt	10.3511	1	zCry4_up_seq	-4133	-4128	+	CACGTG
MA0004.1	Arnt	10.3511	1	zCry4_up_seq	-4133	-4128	-	CACGTG
MA1151.1	RORC	14.6953	0.9895623	zCry4_up_seq	-2814	-2803	-	GTAAATAGGTCA
MA1151.1	RORC	14.2201	0.9800103	zCry4_up_seq	-2833	-2822	-	GAATTTAGGTCA
MA1150.1	RORB	13.8225	0.9789008	zCry4_up_seq	-2834	-2824	-	ATTTAGGTCAA
MA1150.1	RORB	13.3547	0.9685302	zCry4_up_seq	-2815	-2805	-	AAATAGGTCAA
MA0071.1	RORA	12.5175	0.9489851	zCry4_up_seq	-2335	-2326	-	TTAAAGGTCA
MA0072.1	RORA(var.2)	15.7718	0.9440321	zCry4_up_seq	-2834	-2821	-	AGAATTTAGGTCAA
MA0071.1	RORA	12.2604	0.9418858	zCry4_up_seq	-2814	-2805	-	AAATAGGTCA
MA0071.1	RORA	12.1546	0.9389677	zCry4_up_seq	-2833	-2824	-	ATTTAGGTCA
MA0072.1	RORA(var.2)	15.4361	0.9374095	zCry4_up_seq	-2815	-2802	-	TGTAAATAGGTCAA
MA0639.1	DBP	10.6841	0.9205714	zCry4_up_seq	-2270	-2259	+	AATTATGCAATG
MA0639.1	DBP	10.3357	0.9160024	zCry4_up_seq	-2270	-2259	-	CATTGCATAATT
MA0639.1	DBP	10.1368	0.9133942	zCry4_up_seq	-2808	-2797	+	ATTTACATAATA
MA0639.1	DBP	10.0164	0.9118162	zCry4_up_seq	-4060	-4049	+	GATTACATAAAT
MA0639.1	DBP	9.91257	0.9104544	zCry4_up_seq	-3616	-3605	+	GATTGCATAAGC
MA0639.1	DBP	9.78304	0.9087559	zCry4_up_seq	-2808	-2797	-	TATTATGTAAAT
MA0639.1	DBP	9.25471	0.9018283	zCry4_up_seq	-4060	-4049	-	ATTTATGTAATC
MA0639.1	DBP	9.20523	0.9011794	zCry4_up_seq	-3616	-3605	-	GCTTATGCAATC
MA0018.2	CREB1	11.5686	1	zCry4_up_seq	-1952	-1945	+	TGACGTCA
MA0018.2	CREB1	11.5686	1	zCry4_up_seq	-1952	-1945	-	TGACGTCA
MA0018.2	CREB1	9.77186	0.9342022	zCry4_up_seq	-2138	-2131	-	TGAGGTCA
MA0639.1	DBP	11.0493	0.9253598	zCry4_up_seq	-823	-812	-	CATTACATAAAA
MA0639.1	DBP	10.908	0.923507	zCry4_up_seq	-1284	-1273	-	AATTACATAAAA
MA1151.1	RORC	11.2709	0.9207397	zCry4_up_seq	-1769	-1758	-	AAAAATAGGACA
MA0071.1	RORA	11.0822	0.9093602	zCry4_up_seq	-1051	-1042	-	TATAAGGTCA
MA0639.1	DBP	9.64048	0.9068866	zCry4_up_seq	-823	-812	+	TTTTATGTAATG
MA0639.1	DBP	9.18534	0.9009186	zCry4_up_seq	-416	-405	-	ACTTACGTAAAT
MA0004.1	Arnt	10.3511	1	zCry4_Intron1	2109	2114	+	CACGTG
MA0004.1	Arnt	10.3511	1	zCry4_Intron1	2109	2114	-	CACGTG
MA0639.1	DBP	11.6445	0.9331643	zCry4_Intron1	1721	1732	-	CATTGCATAACA
MA0639.1	DBP	10.8681	0.9229834	zCry4_Intron1	1721	1732	+	TGTTATGCAATG
MA1151.1	RORC	10.3873	0.9029807	zCry4_Intron1	803	814	-	AAAACTGTGTCA
MA0476.1	FOS	8.503	0.902344	zCry4_up_seq	-2206	-2196	+	ACTGAGTCAAG
